# RNA 3D Modules in Genome-Wide Predictions of RNA 2D Structure

**DOI:** 10.1371/journal.pone.0139900

**Published:** 2015-10-28

**Authors:** Corinna Theis, Craig L. Zirbel, Christian Höner zu Siederdissen, Christian Anthon, Ivo L. Hofacker, Henrik Nielsen, Jan Gorodkin

**Affiliations:** 1 Center for non-coding RNA in Technology and Health, University of Copenhagen, Frederiksberg, Denmark; 2 Department of Veterinary Clinical and Animal Science, Faculty of Health and Medical Science, University of Copenhagen, Frederiksberg, Denmark; 3 Department of Mathematics and Statistics, Bowling Green State University, Bowling Green, Ohio, United States of America; 4 Institute for Theoretical Chemistry, University of Vienna, Vienna, Austria; 5 Research Group Bioinformatics and Computational Biology, University of Vienna, Vienna, Austria; 6 Department of Cellular and Molecular Medicine, The Panum Institute, University of Copenhagen, Copenhagen, Denmark; 7 Bioinformatics Group, Department of Computer Science, and Interdisciplinary Center for Bioinformatics, University of Leipzig, Leipzig, Germany; 8 Interdisciplinary Center for Bioinformatics, University of Leipzig, Leipzig, Germany; Ben-Gurion University, ISRAEL

## Abstract

Recent experimental and computational progress has revealed a large potential for RNA structure in the genome. This has been driven by computational strategies that exploit multiple genomes of related organisms to identify common sequences and secondary structures. However, these computational approaches have two main challenges: they are computationally expensive and they have a relatively high false discovery rate (FDR). Simultaneously, RNA 3D structure analysis has revealed modules composed of non-canonical base pairs which occur in non-homologous positions, apparently by independent evolution. These modules can, for example, occur inside structural elements which in RNA 2D predictions appear as internal loops. Hence one question is if the use of such RNA 3D information can improve the prediction accuracy of RNA secondary structure at a genome-wide level. Here, we use RNAz in combination with 3D module prediction tools and apply them on a 13-way vertebrate sequence-based alignment. We find that RNA 3D modules predicted by metaRNAmodules and JAR3D are significantly enriched in the screened windows compared to their shuffled counterparts. The initially estimated FDR of 47.0% is lowered to below 25% when certain 3D module predictions are present in the window of the 2D prediction. We discuss the implications and prospects for further development of computational strategies for detection of RNA 2D structure in genomic sequence.

## Introduction

Recent studies have shown a large potential for RNA structure in eukaryotes through experimental strategies [1–4] as well as computational strategies [5]. The computational strategies were developed to search for novel RNA secondary structure by making use of comparative sequence information either from sequence based alignments or by RNA structural alignments directly [6–15]. While further development of these *de novo* methodologies is still needed, many methodologies for class specific searches, e.g. [16–19] and homology based approaches [20, 21] for RNA structure are more specialized and mature. The need for improvements in RNA structure prediction is also supported by the discoveries of new RNA classes [22, 23], and the expectation that many more structured RNAs are hidden in the genomes [24–27]. Of particular interest is the abundance of non-coding (nc)RNAs [28] and regulatory elements embedded in untranslated regions (UTR) of mRNAs, for example riboswitches that control gene expression [29]. In line with the RNA structure potential, further development of computational tools to screen for conserved structured RNAs is indeed desired. While there are many directions which can be pursued, here we consider identifying elements of RNA 3D structure in regions with apparent secondary structure.

A main motivation is that both secondary and tertiary structural elements are key to understand RNA functions. Such structural elements are found in some classes of ncRNAs and the 5’ and 3’ UTRs of mRNA. The first step in searches for such structural elements has traditionally been by establishing a secondary structure diagram based on Watson-Crick (WC) base pairing locally (e.g. hairpins) or more distantly (e.g. pseudoknots). Secondary structure prediction is typically based on energy optimization often involving co-variation analyses as well. However, both co-variation and experimental analyses, including X-ray crystallography, have revealed the existence of non-WC base pairs that provide the RNA structure with architectural features in addition to the regular A-helices [30–33]. Defined sets of non-WC pairs embedded in WC pairs constitute 3D modules such as kink-turns, C-loops, sarcin-ricin motifs or GNRA hairpin loops that are key determinants of RNA structure and function.

Note that *(1)* 3D modules are found in all domains of life (as an example, the kink-turn has been described in Bacteria [34], Archaea [35], and Eukarya [36]); *(2)* 3D modules occur in many different, and unrelated structured RNAs (e.g. riboswitches [37, 38], rRNA [34], and snoRNA [39, 40]); *(3)* The 3D modules function in all aspects of RNA structure and function, including initiation of folding, stabilization of the overall architecture, mediating RNA-protein interactions, binding of small molecule ligands, and catalysis [34, 41–45]. Thus, 3D modules are recurrent building blocks inherent to RNA structure and function, and so we expect to find them in novel RNAs which function by virtue of their secondary and tertiary structure. Incorporating 3D modules into 2D structure prediction tools will therefore push the computational screens for structured RNAs in genomic sequences further towards functional relevancy.

Although more recent development has shown progress with regard to combining 2D and 3D structure prediction, none seem feasible at a genome-wide level. Approaches such as RNA2D3D [46] and ASSEMBLE [47] are semi-automated where the user is in charge of modifying a given secondary structure by adding 3D modules or editing the backbone torsion angles. Combining 3D modules with RNA secondary structures is also used to improve and speed up tertiary structure predictions. Tertiary structure predictors are generally time-consuming and limited to small molecules. Laing and Schlick reviewed these where they explore new ideas towards 3D structure prediction [48]. Reinharz *et al.* [49] developed an automated pipeline, RNA-MoIP, for combining 2D and 3D information to refine tertiary predictions. In a first step, they use a classical secondary structure predictor to get a set of sub-optimal structures. In a second step, a scan for known 3D modules which exactly match portions of the input sequence provides potential insertion sites. Aligning potential 3D modules with the sub-structures by minimizing the number of free nucleotides (nts) results in a set of templates for the MC-Sym pipeline [50]. MC-Sym performs a fragment insertion simulation to build full-atom models of the given secondary structure and the putative module. One advantage of RNA-MoIP is that it uses an integer programming framework which supports the incorporation of more complex modules such as *k*-way junctions. However, with running times around one minute for each window, it is preferable to first develop fast screens for potential secondary and 3D structure before turning to a more detailed tool such as RNA-MoIP.

Hence, including 3D modules in genome-wide screens for RNA 2D structure is a first step towards obtaining a better discrimination between predictions on real and randomized data. Here, we address this challenge by implementing a pipeline consisting of the RNAz tool for genome-wide 2D predictions in sequence based alignments [51] along with the 3D modules programs metaRNAmodules [52] and JAR3D [53], both of which are able to recognize known and novel sequence variants of RNA 3D modules. The pipeline is also run on shuffled alignments (randomized data) to estimate the discriminative power with and without 3D modules.

## Materials and Methods

**Fig 1:**
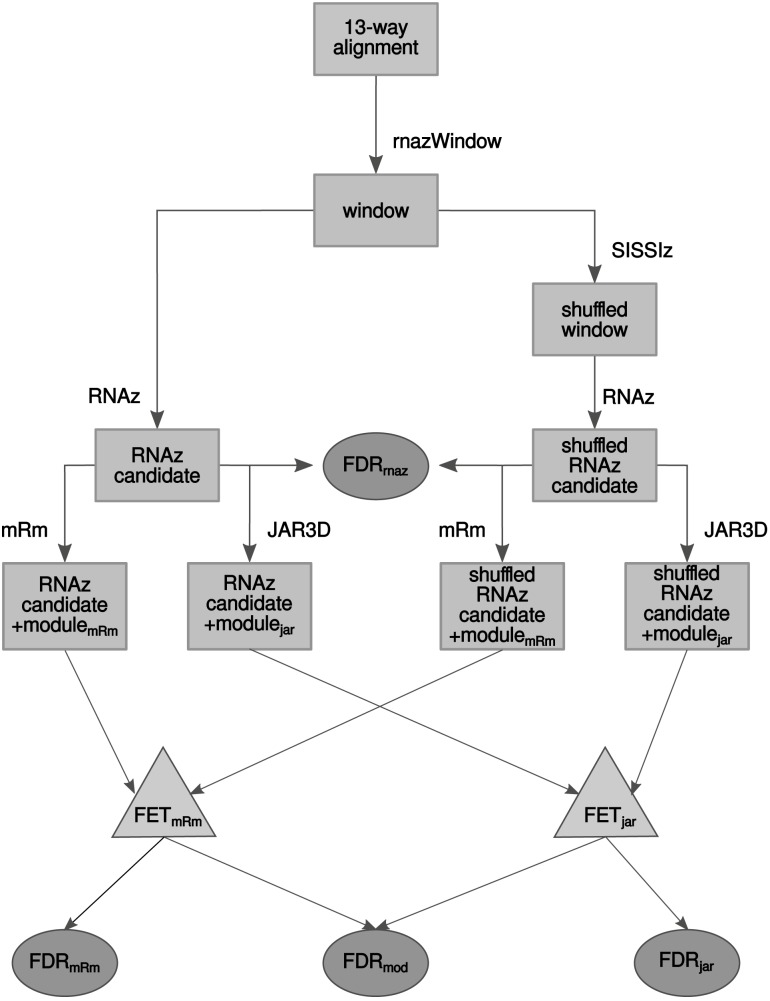
Graphical outline of the approach. The 13-way alignment is sliced into windows of size 40–120 nts. The windows are shuffled by SISSIz and both the original and shuffled data are scanned by RNAz for thermodynamically stable and evolutionarily conserved candidates (p-score > 0.9). For those windows the false discovery rate is estimated (FDR_*rnaz*_). Both the original and shuffled windows are also scanned by metaRNAmodules (mRm) and JAR3D for reliable 3D module predictions. The windows with and without 3D module predictions are counted for a Fisher’s exact test (FET). Windows with 3D module predictions are also used to estimate the FDR.

**Fig 2:**
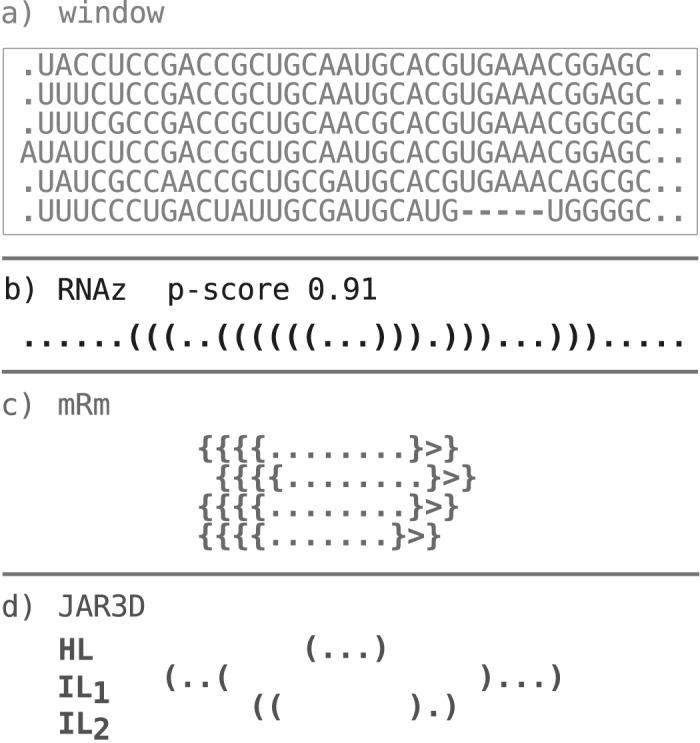
Details of the genome wide scan. (a) Windows (boxed) with the *H. sapiens* sequence as reference are screened with RNAz for (b) structural motifs. The metaRNAmodules models are applied on each sequence of the window (c) whereas the JAR3D models are applied on loop regions of the RNAz consensus structure (d). In both cases, predictions of different models can overlap. The same procedure is used to scan the shuffled data.

The sequences used in this paper are based on a 13-way whole genome vertebrate alignment and a randomized version of the alignment (see below). Both alignments are screened by RNAz for stable and conserved structured regions. We further screen the original and the shuffled alignment with 63 metaRNAmodules models for internal loops, and 276 internal loop (IL) and 253 hairpin loop (HL) models from JAR3D. For further details see Figs 1 and 2. The selected models are shown in Tables C, E, and G in S1 Text.

### Multiple alignments and sliding window construction

For our analysis we use a whole genome alignment computed by MULTIz/Threaded Blockset Aligner [54] containing 13 vertebrate sequences (details in S1 Fig). These sequences are selected as a trade-off between width of the phylogenetic tree and alignment quality (e.g. low gap frequency). The human sequence is the reference sequence. Because the alignment still has low complexity regions, gap-rich regions, or dubious aligned fragments, further pre-processing steps are necessary to filter them out. This is done by using a bundle of auxiliary tools from RNAz [11]. *rnazWindow* is used to perform several filtering steps with default parameters (for details see [11] and the RNAz manual). The alignment is sliced into windows of size ∼ 40–120 nts that overlap to varying degrees. In case the number of sequences in the resulting windows is greater than six, they are reduced to six by optimizing the pairwise sequence identity to 80%. The windows are pre-filtered by pairwise sequence identity greater than or equal to 50% and a GC content in the range of 0.25 to 0.75. Only the forward strand is taken into account. By convention, the reference sequence is always in the selected set otherwise the window is discarded. To provide random background for comparison the windows are shuffled with SISSIz [55], a tool which preserves relevant characteristics of the data, such as gap and local conservation patterns and the dinucleotide content. The *original* and the *shuffled* windows are screened with RNAz for energetically stable and conserved structured regions.

### Predicting secondary structures with RNAz

RNAz version 2.1 predicts structural RNAs in multiple sequence alignments [51]. It is based on two characteristic features of structured RNAs: thermodynamic stability and evolutionarily conserved secondary structure elements of homologous sequences. The thermodynamic stability is measured by a *z*-score and the structural conservation index (SCI) measures the consistency between the average of the individual structures to the predicted consensus structure. Both measures result in a probability, called p-score, used for the classification of a prediction (on an input window of aligned sequences) as “structural RNA” or “other”. A p-score >0.9 indicates high-confidence “structural RNA” candidates although the more relaxed criterion of 0.5 is sometimes used. In [51], the estimated false discovery rate (FDR) of RNAz 2.1 based on ENCODE regions and a dinucleotide background model is found to be 54% for p-score >0.9. In the work reported here, RNAz is applied with default parameters on the pre-processed data. Of the original windows, 161 481 have RNAz p-score >0.9, and of the shuffled windows, 66 891 have RNAz p-score >0.9.

### Eliminating overlapping windows

Counting the number of 3D modules per window is complicated by the fact that the original windows overlap to varying degrees and some 3D modules are found in the overlapping regions. We remove overlapping original windows by listing them from highest RNAz p-score to lowest (all with RNAz p-score >0.9) and report those results in the paper. In the Supplementary Material we show that two other ordering schemes (lowest to highest RNAz p-scores and random ordering) give similar results (see S2 Text, S3 Text, and S4 Text). Once an ordering is chosen, we go through the list and dismiss any original windows that overlap with a window that appears earlier in the list. This reduces the number of original windows to 142 517 but eliminates double-counting difficulties. No such procedure is needed for shuffled windows since they are shuffled independently and thus do not overlap one another.

### Prediction of 3D modules

The 3D module models for our approach are obtained from the metaRNAmodules pipeline as well as JAR3D. The metaRNAmodules pipeline version 1.02 [52] is based on the RMDetect framework [56] and builds probabilistic models for 3D internal loop modules from aligned sequences. 63 non-redundant models, mRm_*IL*_, were selected. New sequences can then be scored against these probabilistic models. Each of these models was used to screen the sequences in original and shuffled windows for matches to 3D modules. By inspection of the RMDetect score distributions we pragmatically find that the top 25% high scoring 3D modules on the original data make up a tail in the score distribution that separates from low-scoring (i.e. less reliable predictions) and from the score distribution over the shuffled windows (see S2 Fig). Thus, we have relative RMDetect score cut-offs for each of the 63 mRm_*IL*_ models. Furthermore, the predictions were filtered for 3D modules that consistently (in location) match at least 50% of the sequences in the alignment. The resulting 3D module predictions themselves (see Results for details) are in the following referred to as M_*mrmIL*_.

JAR3D [53] is based on Stochastic Context-Free Grammars and a Markov Random Field approach to build probabilistic models. We used version 1.13, which contains 276 IL and 253 HL models. JAR3D IL and HL include their flanking Watson-Crick basepairs in addition to the nucleotides interior to the loop. We extract internal loops with ≥ 10 nts and hairpin loops from the consensus structure of the RNAz predictions and submit the corresponding sequences from the six organisms to JAR3D. An input sequence is matched against each probabilistic model and evaluated in terms of the minimum interior edit distance (Levenshtein distance of the interior nts) and alignment score deficit. The alignment score deficit is the difference between the highest alignment score among 3D instances of a particular module and the alignment score of the input sequence. Each module has an acceptance region which favors low minimum interior edit distance and low alignment score deficit. The percentage of accepted sequences in a given position of a multiple alignment is referred to as passed cutoff percentage. We consider JAR3D IL and HL predictions as reliable when their mean interior edit distance is lower than or equal to 4 and at least 50% of the input sequences in the multiple alignment window match the module group (passed cutoff ≥ 50). We refer to those predictions as M_*jarIL*_ and M_*jarHL*_.

Of the original windows with RNAz p-score >0.9 that match a 3D module prediction, 62 overlapped Rfam families with a human sequence, and two were found to overlap human ribosomal RNA sequences, and so these 62 windows were removed, leaving 142 455 original windows with RNAz p-score >0.9.

### Performance evaluation

To evaluate the performance of RNAz we estimate the false discovery rate $\hat{F}$ at a given RNAz p-score larger than a threshold *θ* and a given GC content between *α* and *β* inclusive as

$$\begin{array}{c} {\hat{F}}_{\left[\alpha ,\beta \right]}\left(\theta \right)=\frac{N_{{\text{shuf}}_{\left[\alpha ,\beta \right]}}\left(\theta \right)}{N_{{\text{ori}}_{\left[\alpha ,\beta \right]}}\left(\theta \right)}, \end{array}$$ (1)

where *N*_shuf_[*α*,*β*]__(*θ*) is the number of RNAz predictions with p-score > *θ* and a GC content between *α* and *β* in shuffled windows and *N*_ori_[*α*,*β*]__(*θ*) is the number of RNAz predictions with p-score > *θ* and a GC content between *α* and *β* predicted in original windows, after dismissal of windows to prevent double counting.

When combining RNAz predictions with the 3D module predictions we conduct a one-sided Fisher Exact Test (FET) on individual models to identify significantly enriched 3D modules. Each cell of the FET 2×2 contingency table contains counts of unique windows to preserve the assumption of independence of the observations. As explained above, we use criteria which are specific to mRm_*IL*_ and JAR3D modules; these are listed in Tables A and B in S1 Text. The schemes show tests for the nonrandom association between windows predicted as high-confidence structured RNAs with and without 3D module predictions in original against SISSIz shuffled data. Table 1 shows an example of the counts of unique windows of a contingency table. Note that although the enrichment is modest for each individual module, such enrichment is observed consistently for a number of models (see Results). The FDR for an individual module is computed similarly, using the numbers in columns 2 and 3 of Tables I–K in S1 Text.

**Table 1:** Contingency table for mRm_*IL*_ model RF00177_235_2HHH_1380_1383_1471_1475 showing the number of original (second column) and shuffled windows (third column) with the (“3D module+”) and without the module (“3D module–”). Furthermore, the respective fold change (the ratio of 3D module- and 3D module+ windows and the ratio between original and shuffled windows with and without 3D modules) and the sum (“Total”) of each row and column is shown. The adjusted p-value of the FET equals 9.8 × 10^−4^ with an odds ratio of (227/111 833)/(62/53 326) = 1.746.

FET	original	shuffled	Fold change	Total
**3D module+**	227	62	3.7	289
**3D module–**	111833	53326	2.1	165159
**Fold change**	492.7	860.1		
**Total**	112060	53388		16548

## Results

To investigate the relationship between the 2D and 3D predictions we address the following: (1) whether 3D modules are enriched in the original windows as opposed to the shuffled ones; (2) whether individual 3D modules can be seen to be enriched; (3) even more importantly, we investigate if these models have a collective effect in lowering the FDR compared to RNAz alone.

### Enrichment of 3D modules in RNAz screens

To address the question of whether 3D modules occur more frequently in original windows with RNAz p-score >0.9 than in shuffled windows with RNAz p-score >0.9, we consider if 3D modules occur in the original and shuffled windows at the same nominal rate. A higher rate in the original windows is equivalent to the odds ratio being greater than 1 in Table 1. As a background we assume that there would be a 50% chance of seeing a higher rate in the original windows, and so the number of 3D modules with a higher rate in the original windows would have a binomial distribution with probability 0.5. The 3D modules that were not even found once in the original and shuffled windows were discarded. We find 74.1% (43 out of 58) mRm_*IL*_ models that predict M_*mrmIL*_ which are present at a higher rate in original windows (z-score = 3.6, p-value = 1.0 × 10^−4^). With JAR3D IL models, omitting 3D modules whose 3D sequences have average length < 9 nts, we find 116 out of 173 (67.1%) models that predict M_*jarIL*_ at a higher rate in original windows (z-score = 4.5, p-value = 2.0 × 10^−6^). Thus, the IL modules occur more often in the original windows than in shuffled windows, and so 3D IL modules are enriched in original sequences having apparent secondary structure as opposed to synthetic sequences meeting the same standard for secondary structure. However, the performance of M_*jarHL*_, predicted at a higher rate by 119 out of 246 JAR3D HL models (48.4%), is marginally lower than 50% (z-score = -0.5, p-value = 4.5 × 10^−2^). The number of models decreases when we increase the threshold for the odds ratio (see S4 Fig).

### Enrichment of individual modules

To address the second question, whether there are individual modules which are significantly enriched in original compared to shuffled windows, we conduct a one-sided Fisher’s exact enrichment test and correct for multiple testing. We refer to this test as FET_*fixpscore*_, where we count windows with the 3D module and windows without, cf. Table 1. For M_*mrmIL*_ we increase the list of top 25% highest scoring candidates (see Methods) to top 40% as we otherwise get low counts of many modules. We subject the FET results to a multiple testing correction using the Benjamini-Hochberg procedure [57] at a 5% false discovery rate and we only consider modules with adjusted p-value smaller than or equal to 0.05 to be significantly enriched.

Generally, the results of the enrichment analysis show that a fraction of M_*mrmIL*_, M_*jarIL*_, and M_*jarHL*_ are significantly enriched. The FET_*fixpscore*_ identifies 8 out of 63 (12.7%) distinct mRm_*IL*_, 13 out of 276 (4.7%) JAR3D IL, and 28 out of 253 (11.1%) JAR3D HL models that predict 3D modules that are significantly enriched (adjusted p-value ≤ 0.05, see Tables C–H in S1 Text). Requiring an odds ratio of at least 1.25 results in 6 mRm_*IL*_, 8 JAR3D IL, and 4 JAR3D HL models (see S3 and S4 Figs).

### The impact of 3D modules on the False Discovery Rate

We evaluate the potential of 3D module predictions to lower the FDR in RNAz predictions. Our RNAz screen (p-score >0.9 and a GC content in the range of 0.25 to 0.75) identifies 142 455 candidates in the original windows and 66 891 candidates in the shuffled windows. This corresponds to ${\hat{F}}_{rnaz}$ of 47.0%. For the estimation of combined 2D/3D FDRs we only consider module predictions made by models that are enriched and that have an odds ratio ≥ 1.25, because we want to include only the best modules. We refer to the FDRs of the different classes of 3D module prediction methods as ${\hat{F}}_{mrmIL}$, ${\hat{F}}_{jarIL}$, and ${\hat{F}}_{jarHL}$ and combinations of the methods are abbreviated as ${\hat{F}}_{jarHL\&mrmIL}$, ${\hat{F}}_{jarIL\&mrmIL}$, and ${\hat{F}}_{jarHL\&jarIL}$.

Table 2 shows the results of estimating the FDRs under the different scenarios comparing only windows with the presence of the respective types of 3D modules. M_*jarIL*_ lower the FDR from 47.0% to 36.0%, M_*mrmIL*_ present a reduction to 34.1% whilst M_*jarHL*_ are able to lower the FDR to 37.0%. Windows that contain M_*jarIL*_ and M_*mrmIL*_ lower the FDR to 27.8%. A combination of M_*jarIL*_ and M_*jarHL*_ decrease the FDR to 26.4%. The combination of M_*mrmIL*_ and M_*jarHL*_ shows the best improvement, with a reduction from 47.0% to 24.8%.

**Table 2:** False discovery rates for RNAz candidates (p-score > 0.9, 0.25 ≤ GC content ≤ 0.75) and 3D module predictions. Only 3D modules that are enriched and with an odds ratio ≥ 1.25 are taken into account. Furthermore, JAR3D IL modules have average 3D sequence length ≥ 9. Subscripts denote which method for module prediction is used. “win_*shu*_/win_*ori*_” shows the number of shuffled and original windows that match a 3D module.

	FDR
${\hat{F}}_{rnaz}$	47.0%
${\hat{F}}_{mrmIL}$	34.1%
win_*shu*_/win_*ori*_	815/2390
${\hat{F}}_{jarIL}$	36.0%
win_*shu*_/win_*ori*_	1224/3401
${\hat{F}}_{jarHL}$	37.0%
win_*shu*_/win_*ori*_	2020/5465
${\hat{F}}_{mRmIL\&jarIL}$	27.8%
win_*shu*_/win_*ori*_	25/90
${\hat{F}}_{mRmIL\&jarHL}$	24.8%
win_*shu*_/win_*ori*_	25/101
${\hat{F}}_{jarIL\&jarHL}$	26.4%
win_*shu*_/win_*ori*_	37/140

Some individual modules have strong individual effects on the FDR. Pragmatically, we consider only modules that occur in at least six original windows (see Tables I–K in S1 Text) and models with an adjusted p-value ≤ 0.05 and odds ratio ≥ 1.25. With this we obtain 6 mRm_*IL*_, 8 JAR3D IL, and 4 JAR3D HL models that lower the FDR to 42.3% or lower and of these 3 mRm_*IL*_, 7 JAR3D IL, and 4 JAR3D HL lower the FDR to 37.6% or lower.

## Discussion

The reduction in the FDR shown here comes at the cost of a large reduction in the number of positive hits. This is due to the large number of 3D modules, each of which occurs with a low probability in a given structured RNA.

Large well-structured module groups such as the sarcin-ricin and kink-turn do not occur often enough to achieve a low p-score for the Fisher exact test used for Table E in S1 Text. Nevertheless, they tend to have odds ratios above 1 and so occur more often in original alignments than shuffled alignments.

The way that we restrict attention to internal loops with at least 10 columns in the alignment leads to a few odd-looking results in Table E in S1 Text in which JAR3D module groups with few nucleotides have a low p-value in the Fisher exact test. These occur because one of the six sequences has a long insertion relative to the others, making the internal loop appear to have 10 or more nucleotides, but then the other five sequences match a really small JAR3D module well. It is surprising that such situations appear to occur more often in original windows than in shuffled windows, because SISSIz is designed to preserve the gap distribution.

When the secondary structure is predicted reliably and we can extract the correct loop regions, the use of JAR3D for the 3D module prediction is preferable. In contrast to JAR3D, module prediction with metaRNAmodules is purely based on the sequence and might be more powerful when the secondary structure is less reliably identified. For example, the opening of a few nucleotides of a stem increases the free energy, but the inclusion of a module might result in a more stable conformation.

## Conclusion

There exist different strategies to search for secondary structure in genomic sequences. Their application area is a trade-off between speed and alignment strategy, where some work within the regime of sequence based alignments, whereas others conduct structural RNA alignments at the expense of longer run times. However, these methods do typically not take 3D structure information into account. In contrast the 3D information is often essential and associated with function. Motivated by this we addressed the question of whether RNA 3D module information in combination with 2D genome-wide screens for 2D structure could make an impact.

We employed RNAz for the genome-wide (windows based) screen for structured RNAs which take sequence based alignments as input. It uses RNAalifold [58] for the prediction of the consensus structure of the alignment and minimizes the free energy of a structure without taking either tertiary interactions or any energy contribution of non-canonical base pairs into account.

We showed that the combination of 2D secondary structure predictions and 3D module predictions is useful to improve the structured RNAz predictions by lowering the false discovery rates of RNAz.

We showed that some of the 3D modules are found at higher rates in original (unshuffled windows of) alignments than in shuffled ones. This gave rise to lowering the FDR substantially. Overall, the joint presence of 3D module predictions from mRm and JAR3D lowers the FDR below 28%, using 3D modules which occur at a much higher rate in original windows compared to shuffled ones (odds ratio ≥ 1.25).

Thus, we have shown proof of concept that information of RNA 3D modules can enhance the prediction of RNA 2D structure over the use of RNAz alone. An enrichment test with conservative thresholds for secondary structures and 3D modules reveals individual models that have the potential to distinguish between original and shuffled data. Enriched modules with an odds ratio above a certain threshold are able to lower the FDR from 47.0% to around 25%.

## Supporting Information

S1 Fig Phylogenetic tree. (EPS)

S2 Fig Example of RMDetect score distribution. (EPS)

S3 Fig Odds ratio of Fisher’s exact tests. Number of models for increasing odds ratios for windows with RNAz p-score > 0.9.
(EPS)

S4 Fig Odds ratio of Fisher’s exact tests. Number of models with adjusted p-value ≤ 0.05 for increasing odds ratios for windows with RNAz p-score > 0.9.
(EPS)

S1 Text Fisher’s exact test results and the estimation of the false discovery rates for ordering H. Fisher’s exact test schemes for mRm (Table A) and JAR3D (Table B) modules. Fisher’s exact test results for individual mRm (Table C), all mRm (Table D), individual JAR3D IL (Table E), all JAR3D IL (Table F), individual JAR3D HL (Table G), and all JAR3D HL (Table H) modules. False discovery rates for individual mRm (Table I), JAR3D IL (Table J), and JAR3D HL (Table K) modules.
(PDF)

S2 Text Alternative approaches. The file contains the occurrence rates (Table A), number of significantly enriched modules (Table B), the number of modules that lower the false discovery rate by at least 10% and at least 20% (Table C), and false discovery rates of the different 3D module prediction methods (Table D) for the alternative approaches of ordering L and R.
(PDF)

S3 Text Fisher’s exact test results of alternative approaches. Fisher’s exact test results for individual mRm modules of ordering L (Table A) and of ordering R (Table B), all mRm modules of ordering L and R (Table C), individual JAR3D IL modules of ordering L (Table D) and of ordering R (Table E), all JAR3D IL modules of ordering L and R (Table F), individual JAR3D HL modules of ordering L (Table G) and of ordering R (Table H), and all JAR3D HL modules of ordering L and R (Table I).
(PDF)

S4 Text False discovery rates of alternative approaches. False discovery rates for individual mRm modules of ordering L (Table A) and of ordering R (Table B), individual JAR3D IL modules of ordering L (Table C) and of ordering H (Table D), and individual JAR3D HL modules of ordering L (Table E) and of ordering R (Table F).
(PDF)
