# Supplementary material for: RNA 3D Modules in Genome-Wide Predictions of RNA 2D Structure
Source: PLoS One. 2015 Oct 28;10(10):e0139900. doi: 10.1371/journal.pone.0139900 (PMC4624896; doi:10.1371/journal.pone.0139900)
Supplement: S1 Text — Fisher’s exact test schemes for mRm (Table A) and JAR3D (Table B) modules. Fisher’s exact test results for individual mRm (Table C), all mRm (Table D), individual JAR3D IL (Table E), all JAR3D IL (Table F), individual JAR3D HL (Table G), and all JAR3D HL (Table H) modules. False discovery rates for individual mRm (Table I), JAR3D IL (Table J), and JAR3D HL (Table K) modules. (PDF) [file pone.0139900.s005.pdf]

Supplementary Information 1
Fisher's exact test results and the estimation of the false discovery rates for ordering H.

Table A: **Fisher's exact test scheme (FET$_{fixpscore}$) for mRm$_{IL}$ models.**

# unique windows	**original windows**<br>RNAz	**shuffled windows**<br>RNAz	
3D module+	**A**<br>p-score $>$ 0.9<br>z-score $<$ -2<br>mRm occurrence $\geq$ 50%<br>RMDetect score $\geq$ $Q_{0.6}$	**C**<br>p-score $>$ 0.9<br>z-score $<$ -2<br>mRm occurrence $\geq$ 50%<br>RMDetect score $\geq$ $Q_{0.6}$	A+C
3D module-	**B**<br>p-score $>$ 0.9<br>z-score $<$ -2<br>mRm occurrence $<$ 50%<br>RMDetect score $<$ $Q_{0.6}$	**D**<br>p-score $>$ 0.9<br>z-score $<$ -2<br>mRm occurrence $<$ 50%<br>RMDetect score $<$ $Q_{0.6}$	B+D
	A+B	C+D	n = (A+B+C+D)

Table B: **Fisher's exact test scheme (FET$_{fixpscore}$) for JAR3D IL and JAR3D HL models.** "mied" = mean interior edit distance, "pC" = passed cutoff.

# unique windows	**original windows**<br>RNAz	**shuffled windows**<br>RNAz	
3D module+	**A**<br>p-score $>$ 0.9<br>z-score $<$ -2<br>JAR3D mied $\leq$ 4<br>JAR3D pC $\geq$ 50	**C**<br>p-score $>$ 0.9<br>z-score $<$ -2<br>JAR3D mied $\leq$ 4<br>JAR3D pC $\geq$ 50	A+C
3D module-	**B**<br>p-score $>$ 0.9<br>z-score $<$ -2<br>JAR3D mied $\leq$ 4<br>JAR3D pC $\geq$ 50	**D**<br>p-score $>$ 0.9<br>z-score $<$ -2<br>JAR3D mied $\leq$ 4<br>JAR3D pC $\geq$ 50	B+D
	A+B	C+D	n = (A+B+C+D)

Table C: Results of Fisher's exact test according to scheme shown in Table A for mRm_{IL} module predictions on SISSIz and original data of order H. Yellow highlighted rows have a significant p-value after multiple testing correction using the Benjamini-Hochberg procedure at a 5% false discovery rate (adjusted p-value). "Nuc./Pb" denotes the total strand length and the number of paired bases.

ModelID	A	B	C	D	P-value	Odds ratio	Nuc./Pb	Rank	Adj. p-value
RF00177_206_2Z4M_419_423_456_461	1947	110113	675	52713	1.208166e-13	1.38085	11/10	1	7.6114458e-12
RF00177_235_2HHH_1380_1383_1471_1475	227	111833	62	53326	3.097409e-05	1.745829	9/6	2	0.00098
RF00177_955_2HHH_436_440_463_468	830	111230	310	53078	0.0001	1.277597	11/7	3	0.00229
RF00177_145_2QBF_1222_1228_1259_1265	530	111530	190	53198	0.0003	1.330576	14/10	4	0.00538
RF00177_874_2F4V_1225_1231_1262_1268	521	111539	190	53198	0.0007	1.307883	14/8	5	0.00939
RF00001_196_2QBE_69_77_95_103	2924	109136	1273	52115	0.0033	1.096837	21/16	6	0.03193
RF00001_1334_2GYC_21_28_52_58	1720	110340	728	52660	0.0035	1.127567	15/7	7	0.03193
RF00177_831_2V48_435_440_463_469	344	111716	125	53263	0.0048	1.312123	13/6	8	0.03755
RF00001_449_3CCQ_32_37_43_48	3846	108214	1714	51674	0.0099	1.071486	12/8	9	0.06907
RF00001_5_1N8R_75_81_101_106	1121	110939	471	52917	0.0111	1.135253	15/12	10	0.06971
RF00167_327_1Y26_26_29_49_56	429	111631	168	53220	0.0163	1.217389	12/6	11	0.09331
RF00015_758_2OZB_28_34_42_45	2373	109687	1058	52330	0.0359	1.070057	11/8	12	0.18873
RF00177_35_2B9M_757_761_777_781	682	111378	287	53101	0.0406	1.13293	10/10	13	0.19656
RF00177_32_1VOX_748_752_768_772	615	111445	259	53129	0.0503	1.131993	10/8	14	0.22638
RF00001_1060_3BBO_68_77_96_105	865	111195	377	53011	0.0775	1.093841	23/14	15	0.32559
RF00177_11_2VHP_752_756_772_776	604	111456	260	53128	0.0903	1.107343	10/6	16	0.35558
RF00177_90_1HNW_1281_1285_1308_1312	194	111866	79	53309	0.1323	1.170233	10/10	17	0.49036
RF00162_600_2GIS_17_21_32_39	770	111290	345	53043	0.1792	1.063759	13/11	18	0.57634
RF00177_1114_2VHP_1404_1410_1441_1446	1104	110956	500	52888	0.1796	1.052459	13/6	19	0.57634
RF00177_284_2QBJ_1099_1104_1118_1124	273	111787	117	53271	0.1830	1.111923	13/9	20	0.57634
RF00177_751_1IBL_660_665_676_684	7	112053	1	53387	0.2131	3.334967	15/10	21	0.63928
RF00177_1166_2UXD_1224_1231_1262_1269	286	111774	126	53262	0.2492	1.081612	16/8	22	0.71376
RF00177_192_2QBD_657_661_672_679	53	112007	21	53367	0.2804	1.202478	13/10	23	0.76817
RF00177_1369_2GY9_669_675_686_695	3	112057	0	53388	0.3107	Inf	17/8	24	0.78300
RF00177_524_1VOV_400_408_417_424	3	112057	0	53388	0.3107	Inf	17/6	25	0.78300
RF00177_231_2GYB_766_770_786_790	654	111406	302	53086	0.3401	1.03188	10/8	26	0.79451
RF00177_1595_1N34_1100_1107_1122_1131	10	112050	3	53385	0.3524	1.588126	18/6	27	0.79451
RF00177_418_1S1H_661_665_676_683	57	112003	24	53364	0.3531	1.131566	13/11	28	0.79451
RF00177_857_3BBN_130_136_151_158	12	112048	4	53384	0.3731	1.429327	15/6	29	0.81046
RF00017_631_2J37_181_187_212_216	2995	109065	1415	51973	0.4033	1.008632	12/6	30	0.84691
RF00177_942_1N36_137_143_158_165	11	112049	4	53384	0.4388	1.310245	15/8	31	0.89175
RF00177_1127_2VHO_1404_1411_1440_1446	275	111785	129	53259	0.4657	1.015678	15/6	32	0.91056
RF00177_1303_2HHH_138_143_158_164	63	111997	29	53359	0.4886	1.034983	13/10	33	0.91056
RF00177_1124_2VHP_419_427_452_461	8	112052	3	53385	0.5032	1.270442	19/8	34	0.91056
RF00177_1012_3FIH_120_126_141_148	12	112048	5	53383	0.5155	1.143423	15/10	35	0.91056
RF01857_613_1QZW_192_198_207_213	14	112046	6	53382	0.5203	1.111664	14/6	36	0.91056
RF00177_325_1VS5_121_126_141_147	60	112000	29	53359	0.5753	0.9856957	13/10	37	0.94824
RF00177_324_1VS5_475_484_513_516	3	112057	1	53387	0.6115	1.429293	14/9	38	0.94824
RF00017_1608_1L9A_190_194_205_209	222	111838	109	53279	0.6266	0.9702418	10/8	39	0.94824
RF00177_36_2QB9_862_869_880_885	14	112046	7	53381	0.6411	0.9528416	14/12	40	0.94824
RF00177_244_3DF3_1278_1282_1305_1309	141	111919	70	53318	0.6417	0.9596097	10/8	41	0.94824
RF00177_1_2GYB_490_498_527_529	10	112050	5	53383	0.6523	0.9528433	12/7	42	0.94824
RF00177_171_1HR0_804_807_829_846	1	112059	0	53388	0.6773	Inf	22/9	43	0.94824
RF00177_698_2B64_481_491_518_522	1	112059	0	53388	0.6773	Inf	16/9	44	0.94824
RF00177_351_1I95_804_807_829_846	1	112059	0	53388	0.6773	Inf	22/8	45	0.94824
RF00177_1312_2HGR_59_65_89_93	59	112001	31	53357	0.7134	0.906696	13/11	46	0.97703
RF00177_495_2B64_859_866_877_882	11	112049	7	53381	0.8053	0.7486155	14/8	47	1.00000
RF00177_1592_1N36_436_445_457_468	3	112057	2	53386	0.8056	0.7146099	22/8	48	1.00000
RF00177_432_2QP0_215_220_250_257	32	112028	19	53369	0.8198	0.8023636	14/11	49	1.00000
RF00177_1_1J5E_482_490_519_521	10	112050	7	53381	0.8518	0.6805647	12/7	50	1.00000
RF00177_243_2QBB_382_389_400_406	5	112055	4	53384	0.8710	0.5955032	15/9	51	1.00000
RF00177_1470_1XNQ_1103_1111_1121_1128	1	112059	1	53387	0.8959	0.4764094	17/6	52	1.00000
RF00177_334_1HNW_233_238_268_275	24	112036	17	53371	0.9208	0.6725171	14/10	53	1.00000
RF00169_236_1CQ5_37_41_52_56	99	111961	60	53328	0.9391	0.7859362	10/6	54	1.00000
RF00177_579_2J02_440_446_456_463	847	111213	454	52934	0.9800	0.8879934	15/8	55	1.00000
RF00177_1167_2UXD_399_407_418_425	1	112059	3	53385	0.9892	0.1588046	17/6	56	1.00000
RF00177_87_2QAN_541_548_855_857	10	112050	16	53372	0.9995	0.2977043	11/7	57	1.00000
RF00011_1583_1NBS_177_190_209_219	0	112060	0	53388	1.0000	0	25/7	58	1.00000
RF01118_270_2QBC_13_17_35_55	0	112060	0	53388	1.0000	0	26/10	59	1.00000
RF00177_437_2QBF_488_495_500_509	0	112060	0	53388	1.0000	0	18/11	60	1.00000
RF00177_551_2UXD_494_500_507_515	0	112060	0	53388	1.0000	0	16/8	61	1.00000
RF00177_1133_2VHO_380_389_400_408	0	112060	0	53388	1.0000	0	19/8	62	1.00000
RF00059_318_2GDI_55_59_72_76	5858	106202	3210	50178	1.0000	0.8622435	10/6	63	1.00000

Table D: Results of Fisher's exact test according to scheme shown in Table A for mRm_{IL} module predictions of all mRm_{IL} models together on SISSIz and original data of order H.

Order	Model	A	B	C	D	P-value	Odds Ratio
H	all	24956	87104	11401	41987	1.312117e-05	1.055145

Table E: Results of Fisher's exact test according to scheme shown in Table B for JAR3D IL models for SISSIz and original data of order H. Red highlighted rows have a significant p-value after multiple testing correction using the Benjamini-Hochberg procedure at a 5% false discovery rate (adjusted p-value) and an average 3D sequence length ≥9. "Nuc/Bp" denotes the total strand length and the number of base pairs.

Model	A	B	C	D	P-value	Odds ratio	Nuc/Bp	Rank	Adj. p-value
IL_47875.1	548	111512	149	53239	1.119216e-10	1.755906	4/2	1	3.08903616e-08
IL_96206.3	292	111768	63	53325	5.091232e-10	2.211331	5/4	2	7.02590016e-08
IL_73000.2	250	111810	54	53334	8.917314e-09	2.20835	6/4	3	8.20392888e-07
IL_92109.3	517	111543	150	53238	1.509924e-08	1.645044	4/2	4	1.04184756e-06
IL_28947.2	318	111742	83	53305	1.729345e-07	1.827675	4/2	5	9.5459844e-06
IL_97217.11	336	111724	92	53296	5.133559e-07	1.742201	4/2	6	2.36143714e-05
IL_58586.2	289	111771	77	53311	1.232783e-06	1.790166	6/2	7	4.8606872571e-05
IL_56465.4	471	111589	146	53242	1.599942e-06	1.539217	5/2	8	5.5197999e-05
IL_94430.5	347	111713	102	53286	5.125366e-06	1.622697	5/3	9	0.00016
IL_28644.1	152	111908	33	53355	7.520801e-06	2.196047	5/2	10	0.00021
IL_39199.4	260	111800	72	53316	1.301343e-05	1.722088	5/4	11	0.00030
IL_23448.1	85	111975	13	53375	1.31193e-05	3.116649	6/2	12	0.00030
IL_25300.3	280	111780	81	53307	2.419243e-05	1.648511	4/2	13	0.00051
IL_58291.4	121	111939	25	53363	2.799534e-05	2.30729	6/2	14	0.00055
IL_67887.1	205	111855	55	53333	4.760893e-05	1.777176	5/3	15	0.00088
IL_70173.1	173	111887	44	53344	5.876073e-05	1.874553	6/2	16	0.00095
IL_73276.5	173	111887	44	53344	5.876073e-05	1.874553	7/5	17	0.00095
IL_69799.1	115	111945	25	53363	9.534492e-05	2.192762	5/2	18	0.00145
IL_92027.3	150	111910	37	53351	0.0001	1.932687	6/4	19	0.00145
IL_44540.4	199	111861	55	53333	0.0001	1.725068	5/4	20	0.00167
IL_46648.6	216	111844	62	53326	0.0002	1.661067	5/3	21	0.00210
IL_02809.3	232	111828	69	53319	0.0002	1.603131	8/4	22	0.00287
IL_23262.4	440	111620	154	53234	0.0004	1.362666	10/3	23	0.00529
IL_01080.1	203	111857	60	53328	0.0005	1.613005	7/2	24	0.00544
IL_40845.1	170	111890	48	53340	0.0005	1.688372	7/4	25	0.00607
IL_87904.5	1115	110945	444	52944	0.0006	1.198378	10/5	26	0.00682
IL_63133.1	52	112008	8	53380	0.0007	3.0977	6/3	27	0.00691
IL_79083.3	631	111429	236	53152	0.0007	1.275334	11/4	28	0.00691
IL_34628.2	54	112006	9	53379	0.0010	2.859419	12/6	29	0.00920
IL_86357.3	76	111984	16	53372	0.0010	2.263858	6/3	30	0.00943
IL_74876.2	846	111214	332	53056	0.0013	1.215622	10/4	31	0.01163
IL_21333.2	238	111822	77	53311	0.0015	1.473584	8/4	32	0.01259
IL_90459.3	476	111584	176	53212	0.0020	1.289691	6/3	33	0.01642
IL_24982.5	1319	110741	545	52843	0.0025	1.154844	10/5	34	0.01969
IL_87065.1	133	111927	38	53350	0.0025	1.66827	5/3	35	0.01969
IL_43316.1	807	111253	320	53068	0.0027	1.202921	10/4	36	0.02041
IL_83856.1	529	111531	201	53187	0.0031	1.255039	12/6	37	0.02326
IL_47174.11	273	111787	94	53294	0.0033	1.384618	6/3	38	0.02362
IL_91089.1	42	112018	7	53381	0.0036	2.859219	8/2	39	0.02538

Continued on next page

Table E – continued from previous page

Model	A	B	C	D	P-value	Odds ratio	Nuc/Bp	Rank	Adj. p-value
IL_88119.1	187	111873	60	53328	0.0038	1.48566	6/3	40	0.02606
IL_63952.1	502	111558	191	53197	0.0041	1.25327	13/4	41	0.02731
IL_39980.1	100	111960	27	53361	0.0042	1.765208	6/3	42	0.02731
IL_80348.3	177	111883	57	53331	0.0050	1.480175	5/2	43	0.03172
IL_80494.2	478	111582	182	53206	0.0051	1.252307	12/5	44	0.03172
IL_43877.1	87	111973	23	53365	0.0058	1.80274	6/2	45	0.03553
IL_68767.1	100	111960	28	53360	0.0064	1.702133	6/2	46	0.03832
IL_37406.1	83	111977	22	53366	0.0071	1.798	7/2	47	0.04109
IL_42251.1	199	111861	67	53321	0.0071	1.415802	6/3	48	0.04109
IL_63253.1	91	111969	25	53363	0.0074	1.734772	6/2	49	0.04146
IL_12147.1	196	111864	66	53322	0.0076	1.415575	6/3	50	0.04178
IL_17212.2	210	111850	72	53316	0.0082	1.390324	6/3	51	0.04462
IL_26971.1	856	111204	351	53037	0.0090	1.16311	10/3	52	0.04790
IL_98421.4	100	111960	29	53359	0.0096	1.643409	7/2	53	0.04982
IL_21639.1	43	112017	9	53379	0.0122	2.276724	9/3	54	0.06258
IL_82444.1	171	111889	58	53330	0.0134	1.405265	6/3	55	0.06732
IL_86059.1	107	111953	33	53355	0.0155	1.545283	4/2	56	0.07652
IL_41791.1	70	111990	19	53369	0.0158	1.75571	11/8	57	0.07672
IL_09587.1	2706	109354	1199	52189	0.0176	1.07709	11/4	58	0.08378
IL_37104.3	192	111868	68	53320	0.0191	1.345827	6/3	59	0.08933
IL_37197.1	654	111406	268	53120	0.0195	1.163558	11/4	60	0.08973
IL_55649.1	391	111669	153	53235	0.0205	1.218264	8/3	61	0.09085
IL_70237.3	922	111138	388	53000	0.0206	1.133208	10/4	62	0.09085
IL_46721.1	92	111968	28	53360	0.0207	1.565851	10/3	63	0.09085
IL_39585.1	1003	111057	425	52963	0.0218	1.125476	10/6	64	0.09379
IL_39355.1	346	111714	134	53254	0.0221	1.230853	8/3	65	0.09379
IL_18675.1	88	111972	27	53361	0.0253	1.553218	7/2	66	0.10529
IL_37325.1	48	112012	12	53376	0.0256	1.906074	7/4	67	0.10529
IL_31006.1	179	111881	64	53324	0.0265	1.333072	11/5	68	0.10656
IL_55938.4	290	111770	111	53277	0.0266	1.245313	7/4	69	0.10656
IL_15205.1	120	111940	40	53348	0.0279	1.42974	12/5	70	0.10993
IL_31039.1	61	111999	17	53371	0.0286	1.7099	7/3	71	0.11107
IL_13777.1	114	111946	38	53350	0.0315	1.429718	5/2	72	0.12072
IL_56513.1	98	111962	32	53356	0.0358	1.459447	5/2	73	0.13321
IL_16166.4	130	111930	45	53343	0.0360	1.376801	5/2	74	0.13321
IL_80093.1	633	111427	264	53124	0.0362	1.143133	10/5	75	0.13321
IL_06808.1	80	111980	25	53363	0.0375	1.524926	8/4	76	0.13376
IL_98566.1	80	111980	25	53363	0.0375	1.524926	8/2	77	0.13376
IL_69106.1	187	111873	69	53319	0.0382	1.291615	7/3	78	0.13376
IL_95150.6	67	111993	20	53368	0.0383	1.59637	7/3	79	0.13376
IL_06177.1	40	112020	10	53378	0.0401	1.90601	6/2	80	0.13669
IL_31066.3	40	112020	10	53378	0.0401	1.90601	5/2	81	0.13669
IL_69536.1	1089	110971	471	52917	0.0408	1.102532	8/3	82	0.13716
IL_85510.1	684	111376	289	53099	0.0454	1.128367	6/2	83	0.14875
IL_57364.1	25	112035	5	53383	0.0458	2.382414	8/4	84	0.14875
IL_61730.1	25	112035	5	53383	0.0458	2.382414	11/3	85	0.14875
IL_13959.4	154	111906	56	53332	0.0464	1.31064	8/4	86	0.14878
IL_15840.2	694	111366	294	53094	0.0478	1.125389	11/6	87	0.15080
IL_06471.1	477	111583	197	53191	0.0485	1.15422	10/7	88	0.15080
IL_31663.1	93	111967	31	53357	0.0486	1.429637	7/4	89	0.15080
IL_54450.1	41	112019	11	53377	0.0549	1.776038	8/3	90	0.16840
IL_25271.2	27	112033	6	53382	0.0563	2.144171	14/7	91	0.17079
IL_41766.6	336	111724	136	53252	0.0585	1.177564	8/4	92	0.17545
IL_05723.1	473	111587	197	53191	0.0599	1.144501	10/5	93	0.17771
IL_80714.1	463	111597	193	53195	0.0632	1.143507	12/3	94	0.18556
IL_65553.8	107	111953	38	53350	0.0685	1.341875	12/7	95	0.19891
IL_50911.1	34	112026	9	53379	0.0731	1.800058	7/3	96	0.21009
IL_05684.1	223	111837	88	53300	0.0738	1.207692	8/3	97	0.21009
IL_97296.1	209	111851	82	53306	0.0750	1.214676	7/3	98	0.21123

Continued on next page

Table E – continued from previous page

Model	A	B	C	D	P-value	Odds ratio	Nuc/Bp	Rank	Adj. p-value
IL_12211.1	127	111933	47	53341	0.0788	1.287636	12/6	99	0.21948
IL_90133.3	141	111919	53	53335	0.0795	1.267764	7/3	100	0.21948
IL_34363.2	19	112041	4	53384	0.0920	2.263215	13/9	101	0.25133
IL_06847.1	6	112054	0	53388	0.0965	Inf	11/4	102	0.25869
IL_46435.1	6	112054	0	53388	0.0965	Inf	13/2	103	0.25869
IL_20775.1	62	111998	21	53367	0.1057	1.406828	7/3	104	0.28056
IL_17603.1	338	111722	142	53246	0.1123	1.13442	11/5	105	0.29522
IL_30381.1	66	111994	23	53365	0.1170	1.367377	8/4	106	0.30209
IL_80505.1	9	112051	1	53387	0.1171	4.288512	11/2	107	0.30209
IL_79955.2	357	111703	151	53237	0.1183	1.126776	12/6	108	0.30239
IL_17066.1	65	111995	23	53365	0.1312	1.346655	6/3	109	0.33217
IL_13069.3	79	111981	29	53359	0.1346	1.298003	4/2	110	0.33784
IL_87548.1	407	111653	175	53213	0.1370	1.108414	11/6	111	0.34061
IL_93424.4	606	111454	266	53122	0.1397	1.085847	9/4	112	0.34423
IL_28572.2	5	112055	0	53388	0.1425	Inf	15/5	113	0.34767
IL_00998.1	97	111963	37	53351	0.1439	1.249185	8/4	114	0.34767
IL_97509.1	703	111357	311	53077	0.1449	1.077415	10/5	115	0.34767
IL_77263.2	279	111781	118	53270	0.1508	1.126768	10/5	116	0.35876
IL_41139.1	54	112006	19	53369	0.1547	1.354253	7/2	117	0.36493
IL_50521.1	49	112011	17	53371	0.1586	1.373415	14/7	118	0.37092
IL_22909.1	98	111962	38	53350	0.1616	1.228844	9/3	119	0.37470
IL_11751.1	34	112026	11	53377	0.1680	1.472721	7/2	120	0.38635
IL_06211.3	129	111931	52	53336	0.1740	1.182091	7/2	121	0.39687
IL_57785.5	48	112012	17	53371	0.1789	1.345383	8/4	122	0.40429
IL_53635.3	99	111961	39	53349	0.1802	1.209546	11/5	123	0.40429
IL_09491.1	31	112029	10	53378	0.1818	1.477041	7/2	124	0.40458
IL_43124.2	173	111887	72	53316	0.1853	1.144955	7/4	125	0.40915
IL_64589.1	57	112003	21	53367	0.1878	1.29325	7/2	126	0.41147
IL_41153.1	28	112032	9	53379	0.1971	1.482323	7/3	127	0.42842
IL_08926.3	100	111960	40	53348	0.1997	1.191212	5/3	128	0.43067
IL_93568.2	196	111864	83	53305	0.2020	1.125261	12/5	129	0.43213
IL_77014.1	4	112056	0	53388	0.2104	Inf	11/5	130	0.44657
IL_47758.2	115	111945	47	53341	0.2121	1.165875	8/4	131	0.44657
IL_46306.1	1144	110916	522	52866	0.2136	1.04457	9/3	132	0.44657
IL_85647.3	15	112045	4	53384	0.2154	1.786687	15/8	133	0.44690
IL_88865.1	27	112033	9	53379	0.2283	1.429385	8/3	134	0.47013
IL_24546.4	127	111933	53	53335	0.2337	1.141771	10/3	135	0.47783
IL_87507.1	204	111856	88	53300	0.2378	1.104623	9/4	136	0.48250
IL_23639.1	1153	110907	529	52859	0.2440	1.038788	9/4	137	0.49152
IL_47444.3	718	111342	326	53062	0.2458	1.049618	12/6	138	0.49166
IL_21254.1	38	112022	14	53374	0.2527	1.2932	12/7	139	0.50174
IL_25380.1	165	111895	71	53317	0.2599	1.107335	8/4	140	0.51241
IL_71685.1	74	111986	30	53358	0.2630	1.17528	14/7	141	0.51472
IL_78744.1	21	112039	7	53381	0.2729	1.429362	12/6	142	0.52812
IL_11778.1	35	112025	13	53375	0.2736	1.282722	7/2	143	0.52812
IL_11302.1	842	111218	386	53002	0.2757	1.039527	10/3	144	0.52842
IL_70299.1	82	111978	34	53354	0.2829	1.149121	10/4	145	0.53222
IL_39526.4	6	112054	1	53387	0.2835	2.858622	9/2	146	0.53222
IL_98591.3	6	112054	1	53387	0.2835	2.858622	13/8	147	0.53222
IL_07300.2	125	111935	54	53334	0.3034	1.102943	9/4	148	0.56309
IL_09333.1	307	111753	138	53250	0.3040	1.060033	12/6	149	0.56309
IL_43946.1	13	112047	4	53384	0.3137	1.548436	12/6	150	0.57372
IL_31555.5	593	111467	272	53116	0.3157	1.038862	9/4	151	0.57372
IL_75328.1	20	112040	7	53381	0.3160	1.361309	14/4	152	0.57372
IL_09530.1	656	111404	302	53086	0.3240	1.03506	8/3	153	0.57844
IL_91379.1	8	112052	2	53386	0.3246	1.905751	12/2	154	0.57844
IL_06421.1	102	111958	44	53344	0.3249	1.104527	7/4	155	0.57844
IL_99397.1	15	112045	5	53383	0.3332	1.429339	9/4	156	0.58949
IL_06468.1	244	111816	110	53278	0.3376	1.056917	10/4	157	0.59271

Continued on next page

Table E – continued from previous page

Model	A	B	C	D	P-value	Odds ratio	Nuc/Bp	Rank	Adj. p-value
IL_06306.1	33	112027	13	53375	0.3418	1.209423	13/2	158	0.59271
IL_91044.1	338	111722	154	53234	0.3421	1.045794	8/3	159	0.59271
IL_22732.1	166	111894	74	53314	0.3446	1.068834	9/5	160	0.59271
IL_31224.1	282	111778	128	53260	0.3457	1.049745	7/2	161	0.59271
IL_59529.1	491	111569	226	53162	0.3499	1.035191	10/5	162	0.59612
IL_37347.1	14	112046	5	53383	0.3893	1.334071	15/7	163	0.65923
IL_40892.1	98	111962	44	53344	0.4102	1.061177	8/3	164	0.69026
IL_47732.1	18	112042	7	53381	0.4140	1.225099	6/2	165	0.69089
IL_83920.1	57	112003	25	53363	0.4155	1.086287	7/3	166	0.69089
IL_54966.1	282	111778	131	53257	0.4284	1.025669	9/5	167	0.70799
IL_52958.1	2612	109448	1237	52151	0.4381	1.00614	9/2	168	0.71966
IL_30067.1	268	111792	125	53263	0.4459	1.021518	10/5	169	0.72829
IL_25082.1	2	112058	0	53388	0.4588	Inf	17/6	170	0.73188
IL_82601.1	2	112058	0	53388	0.4588	Inf	8/4	171	0.73188
IL_94403.5	2	112058	0	53388	0.4588	Inf	11/3	172	0.73188
IL_94744.1	2	112058	0	53388	0.4588	Inf	17/8	173	0.73188
IL_45262.4	140	111920	65	53323	0.4647	1.026198	9/3	174	0.73383
IL_58454.1	570	111490	269	53119	0.4653	1.009573	9/3	175	0.73383
IL_86336.1	283	111777	133	53255	0.4716	1.013784	9/2	176	0.73921
IL_06180.1	899	111161	426	52962	0.4764	1.005455	9/4	177	0.73921
IL_38807.3	57	112003	26	53362	0.4791	1.044486	11/6	178	0.73921
IL_09882.1	97	111963	45	53343	0.4812	1.027004	12/6	179	0.73921
IL_02835.1	4	112056	1	53387	0.4821	1.905719	13/4	180	0.73921
IL_28003.1	61	111999	28	53360	0.4856	1.037924	8/4	181	0.74040
IL_35043.1	6	112054	2	53386	0.4946	1.429304	10/3	182	0.74844
IL_52509.1	497	111563	236	53152	0.5009	1.00333	10/4	183	0.74844
IL_21077.1	159	111901	75	53313	0.5028	1.010035	8/2	184	0.74844
IL_71942.1	8	112052	3	53385	0.5032	1.270442	10/2	185	0.74844
IL_97057.3	675	111385	321	53067	0.5044	1.001836	11/5	186	0.74844
IL_70401.1	10	112050	4	53384	0.5100	1.191058	11/3	187	0.75267
IL_97191.1	14	112046	6	53382	0.5203	1.111664	13/7	188	0.76387
IL_33842.1	26	112034	12	53376	0.5410	1.032227	7/4	189	0.78381
IL_87394.1	26	112034	12	53376	0.5410	1.032227	10/2	190	0.78381
IL_83250.1	111	111949	53	53335	0.5424	0.9977901	11/5	191	0.78381
IL_03110.1	361	111699	173	53215	0.5459	0.9941362	13/5	192	0.78468
IL_52173.1	163	111897	79	53309	0.5799	0.9829654	9/2	193	0.82890
IL_80652.1	15	112045	7	53381	0.5826	1.020924	13/6	194	0.82890
IL_54954.1	3	112057	1	53387	0.6115	1.429293	16/7	195	0.86111
IL_86981.1	3	112057	1	53387	0.6115	1.429293	15/8	196	0.86111
IL_46034.3	81	111979	40	53348	0.6154	0.9647518	8/3	197	0.86217
IL_45794.1	40	112020	20	53368	0.6289	0.9528306	11/5	198	0.87662
IL_57977.1	60	112000	30	53358	0.6333	0.9528222	11/4	199	0.87828
IL_91078.1	12	112048	6	53382	0.6458	0.9528424	12/4	200	0.89116
IL_49527.1	742	111318	362	53026	0.6580	0.9763611	10/4	201	0.89224
IL_82563.1	43	112017	22	53366	0.6618	0.9311666	10/3	202	0.89224
IL_48918.3	73	111987	37	53351	0.6623	0.9399326	12/6	203	0.89224
IL_82188.2	221	111839	110	53278	0.6700	0.9570945	14/3	204	0.89224
IL_97833.1	25	112035	13	53375	0.6721	0.9161831	14/8	205	0.89224
IL_05513.1	1	112059	0	53388	0.6773	Inf	12/3	206	0.89224
IL_27668.1	1	112059	0	53388	0.6773	Inf	11/3	207	0.89224
IL_42891.1	1	112059	0	53388	0.6773	Inf	14/3	208	0.89224
IL_46301.1	245	111815	122	53266	0.6774	0.9566577	9/3	209	0.89224
IL_26868.1	388	111672	192	53196	0.6841	0.9626581	10/5	210	0.89224
IL_12507.1	78	111982	40	53348	0.6865	0.9289781	11/4	211	0.89224
IL_24293.1	70	111990	36	53352	0.6866	0.9263349	8/3	212	0.89224
IL_49493.4	104	111956	53	53335	0.6887	0.9348096	13/7	213	0.89224
IL_87523.1	124	111936	63	53325	0.6918	0.9376546	10/3	214	0.89224
IL_75415.1	4	112056	2	53386	0.7012	0.9528458	10/2	215	0.90019
IL_65137.1	89	111971	46	53342	0.7083	0.921716	12/6	216	0.90505

Continued on next page

Table E – continued from previous page

Model	A	B	C	D	P-value	Odds ratio	Nuc/Bp	Rank	Adj. p-value
IL_92321.4	469	111591	233	53155	0.7148	0.9588144	12/6	217	0.90910
IL_16415.2	14	112046	8	53380	0.7434	0.8337358	14/7	218	0.93792
IL_03282.1	105	111955	55	53333	0.7455	0.9094543	10/5	219	0.93792
IL_89794.1	25	112035	14	53374	0.7476	0.850735	11/7	220	0.93792
IL_27640.1	2	112058	1	53387	0.7548	0.9528466	15/4	221	0.94267
IL_54470.1	321	111739	163	53225	0.7628	0.9380565	8/4	222	0.94518
IL_53988.1	94	111966	50	53338	0.7655	0.8955935	12/5	223	0.94518
IL_82650.1	10	112050	6	53382	0.7671	0.7940454	13/5	224	0.94518
IL_95652.3	26	112034	15	53373	0.7783	0.8257775	14/7	225	0.95468
IL_37053.1	228	111832	118	53270	0.7855	0.9203875	12/6	226	0.95934
IL_44067.4	13	112047	8	53380	0.7921	0.7741915	14/6	227	0.96127
IL_85805.1	919	111141	458	52930	0.7941	0.955604	8/2	228	0.96127
IL_78513.1	18	112042	11	53377	0.8043	0.7795948	8/2	229	0.96393
IL_76486.1	3	112057	2	53386	0.8056	0.7146099	15/8	230	0.96393
IL_31754.1	6	112054	4	53384	0.8082	0.7146022	13/7	231	0.96393
IL_66744.1	74	111986	41	53347	0.8103	0.8598041	13/3	232	0.96393
IL_96446.1	112	111948	61	53327	0.8225	0.8746273	10/3	233	0.97156
IL_81398.1	3139	108921	1538	51850	0.8237	0.971539	10/4	234	0.97156
IL_92114.3	69	111991	39	53349	0.8311	0.8428199	9/4	235	0.97610
IL_59934.1	82	111978	46	53342	0.8373	0.8491786	12/5	236	0.97646
IL_60649.1	46	112014	27	53361	0.8385	0.8116276	11/5	237	0.97646
IL_72158.3	118	111942	65	53323	0.8461	0.8647589	12/6	238	0.98122
IL_31707.1	8	112052	6	53382	0.8703	0.6351977	11/4	239	1.00000
IL_62499.7	462	111598	242	53146	0.8918	0.9091647	7/4	240	1.00000
IL_05221.1	3	112057	3	53385	0.9100	0.4764001	15/11	241	1.00000
IL_25307.1	55	112005	35	53353	0.9256	0.7485168	9/3	242	1.00000
IL_46990.1	1716	110344	870	52518	0.9363	0.9387677	10/2	243	1.00000
IL_98924.1	398	111662	215	53173	0.9364	0.8815234	9/3	244	1.00000
IL_90735.1	19	112041	15	53373	0.9488	0.6033948	16/3	245	1.00000
IL_52940.1	226	111834	129	53259	0.9542	0.8343456	14/6	246	1.00000
IL_94973.1	12	112048	11	53377	0.9623	0.5196644	14/6	247	1.00000
IL_27243.1	1	112059	2	53386	0.9664	0.2382086	14/7	248	1.00000
IL_47687.1	226	111834	133	53255	0.9760	0.8091979	13/6	249	1.00000
IL_30840.2	2289	109771	1171	52217	0.9781	0.9298526	11/5	250	1.00000
IL_53323.1	989	111071	528	52860	0.9838	0.891439	10/3	251	1.00000
IL_25181.1	1011	111049	540	52848	0.9851	0.8909926	13/5	252	1.00000
IL_68827.1	26	112034	23	53365	0.9890	0.5384348	11/4	253	1.00000
IL_49976.1	1036	111024	589	52799	0.9997	0.8364907	8/5	254	1.00000
IL_02359.3	0	112060	0	53388	1.0000	0	16/9	255	1.00000
IL_16330.1	0	112060	0	53388	1.0000	0	25/5	256	1.00000
IL_17682.1	0	112060	1	53387	1.0000	0	17/8	257	1.00000
IL_21421.1	0	112060	0	53388	1.0000	0	20/7	258	1.00000
IL_21495.1	0	112060	0	53388	1.0000	0	14/5	259	1.00000
IL_23414.1	0	112060	0	53388	1.0000	0	21/5	260	1.00000
IL_25230.3	0	112060	0	53388	1.0000	0	18/8	261	1.00000
IL_33964.1	0	112060	0	53388	1.0000	0	20/6	262	1.00000
IL_39324.1	0	112060	0	53388	1.0000	0	18/10	263	1.00000
IL_40527.1	0	112060	0	53388	1.0000	0	15/5	264	1.00000
IL_52610.1	0	112060	0	53388	1.0000	0	36/9	265	1.00000
IL_57285.1	0	112060	0	53388	1.0000	0	13/4	266	1.00000
IL_76095.3	0	112060	0	53388	1.0000	0	18/8	267	1.00000
IL_76263.1	0	112060	0	53388	1.0000	0	17/3	268	1.00000
IL_77076.1	0	112060	0	53388	1.0000	0	18/8	269	1.00000
IL_77296.1	0	112060	0	53388	1.0000	0	14/2	270	1.00000
IL_88367.1	0	112060	0	53388	1.0000	0	15/7	271	1.00000
IL_89028.6	0	112060	0	53388	1.0000	0	13/5	272	1.00000
IL_90057.1	0	112060	0	53388	1.0000	0	23/17	273	1.00000
IL_91904.1	0	112060	0	53388	1.0000	0	13/4	274	1.00000
IL_97842.1	0	112060	0	53388	1.0000	0	16/5	275	1.00000

Continued on next page

Table E – continued from previous page

Model	A	B	C	D	P-value	Odds ratio	Nuc/Bp	Rank	Adj. p-value
IL_98655.1	0	112060	0	53388	1.0000	0	13/5	276	1.00000

Table F: Results of Fisher's exact test according to scheme shown in Table B for JAR3D IL module predictions from all JAR3D IL models with mean interior edit distance ≤ 4, passed cutoff ≥ 50, and mean sequence length ≥ 9.

Order	Model	A	B	C	D	P-value	Odds ratio
H	all	20309	88776	9324	43117	2.311515e-05	1.057894

Table G: Results of Fisher's exact test according to scheme shown in Table B for JAR3D HL models for SISSIz and original data of order H. Brown highlighted rows have a significant p-value after multiple testing correction using the Benjamini-Hochberg procedure at a 5% false discovery rate (adjusted p-value). "Nuc/Bp" denotes the total strand length and the number of base pairs.

Model	A	B	C	D	P-value	Odds ratio	Nuc/Bp	Rank	Adj. p-value
HL_75759.4	5748	106312	2287	51101	2.237763e-14	1.208056	3/1	1	5.66154039e-12
HL_35865.1	5930	106130	2436	50952	9.595822e-11	1.168675	5/1	2	1.213871483e-08
HL_06643.3	5389	106671	2233	51155	5.498795e-09	1.157326	5/2	3	4.046182195e-07
HL_87554.1	1892	110168	705	52683	6.397126e-09	1.283306	5/1	4	4.046182195e-07
HL_90542.1	1019	111041	358	53030	2.095358e-07	1.359375	7/2	5	1.060251148e-05
HL_74559.1	2813	109247	1138	52250	1.050563e-06	1.182218	4/1	6	4.42987398333333e-05
HL_10378.1	1741	110319	674	52714	1.656569e-06	1.234251	4/1	7	5.98731367142857e-05
HL_42677.2	4580	107480	1935	51453	2.808353e-06	1.133089	3/1	8	8.57361902222222e-05
HL_97499.1	2075	109985	823	52565	3.049904e-06	1.204962	4/1	9	8.57361902222222e-05
HL_30008.1	2025	110035	807	52581	6.625207e-06	1.199062	5/1	10	0.00017
HL_63941.1	4447	107613	1903	51485	3.062551e-05	1.118	4/1	11	0.00070
HL_74505.1	4165	107895	1779	51609	4.100912e-05	1.119852	4/1	12	0.00084
HL_05361.1	557	111503	192	53196	4.302211e-05	1.384057	6/2	13	0.00084
HL_59604.1	2278	109782	940	52448	8.562144e-05	1.157762	3/1	14	0.00155
HL_65071.1	961	111099	366	53022	0.0001	1.253071	4/1	15	0.00193
HL_75850.1	3955	108105	1705	51683	0.0002	1.108976	5/1	16	0.00345
HL_99207.1	1858	110202	764	52624	0.0003	1.161294	7/3	17	0.00397
HL_34027.2	7166	104894	3180	50208	0.0003	1.078635	6/1	18	0.00402
HL_57514.2	9336	102724	4207	49181	0.0009	1.062467	4/1	19	0.01162
HL_86115.1	4107	107953	1795	51593	0.0010	1.09349	4/2	20	0.01162
HL_59225.1	1996	110064	838	52550	0.0010	1.13721	5/1	21	0.01162
HL_17790.1	2159	109901	916	52472	0.0015	1.125332	3/1	22	0.01723
HL_17723.4	1774	110286	745	52643	0.0018	1.136618	7/1	23	0.01977
HL_03785.1	3813	108247	1675	51713	0.0025	1.087514	5/1	24	0.02585
HL_35619.2	1466	110594	615	52773	0.0039	1.13746	5/2	25	0.03638
HL_75579.2	16159	95901	7437	45951	0.0039	1.041093	3/1	26	0.03638
HL_00721.1	1325	110735	552	52836	0.0039	1.145297	2/1	27	0.03638
HL_26579.1	667	111393	262	53126	0.0040	1.21413	5/2	28	0.03638
HL_45018.3	876	111184	358	53030	0.0072	1.167067	10/4	29	0.06227
HL_73465.1	29	112031	4	53384	0.0074	3.454503	11/2	30	0.06227
HL_48116.2	8835	103225	4028	49360	0.0081	1.048838	6/1	31	0.06538
HL_42077.2	15066	96994	6949	46439	0.0083	1.038041	5/2	32	0.06538

Continued on next page

Table G – continued from previous page

Model	A	B	C	D	P-value	Odds ratio	Nuc/Bp	Rank	Adj. p-value
HL_68435.1	839	111221	343	53045	0.0085	1.166597	7/2	33	0.06538
HL_99584.1	1330	110730	563	52825	0.0093	1.126977	4/1	34	0.06897
HL_18587.1	842	111218	348	53040	0.0131	1.153871	8/2	35	0.09448
HL_00090.1	757	111303	311	53077	0.0142	1.160729	11/1	36	0.10002
HL_01418.1	1551	110509	671	52717	0.0184	1.102658	5/1	37	0.12570
HL_72273.1	752	111308	312	53076	0.0206	1.149295	6/2	38	0.13603
HL_46489.2	955	111105	403	52985	0.0210	1.130095	9/2	39	0.13603
HL_24707.1	1715	110345	748	52640	0.0218	1.093766	4/2	40	0.13639
HL_19905.3	8008	104052	3670	49718	0.0221	1.04261	4/1	41	0.13639
HL_98523.2	4979	107081	2257	51131	0.0230	1.053376	6/1	42	0.13828
HL_33277.1	1603	110457	701	52687	0.0294	1.090748	7/1	43	0.17270
HL_41827.1	1157	110903	501	52887	0.0378	1.101285	7/2	44	0.21584
HL_98833.3	750	111310	317	53071	0.0384	1.128035	8/2	45	0.21584
HL_34108.2	9755	102305	4515	48873	0.0471	1.032148	4/1	46	0.25911
HL_78420.1	560	111500	234	53154	0.0484	1.140854	9/1	47	0.26057
HL_36842.1	2027	110033	904	52484	0.0494	1.069519	8/2	48	0.26059
HL_44467.1	41	112019	11	53377	0.0549	1.776038	8/1	49	0.28353
HL_24711.1	724	111336	311	53077	0.0662	1.109806	7/2	50	0.33506
HL_56809.1	92	111968	32	53356	0.0724	1.370053	4/1	51	0.35409
HL_95716.1	5882	106178	2711	50677	0.0728	1.035553	5/1	52	0.35409
HL_21675.3	7496	104564	3470	49918	0.0749	1.031277	5/2	53	0.35733
HL_30128.1	939	111121	411	52977	0.0787	1.089216	9/2	54	0.36868
HL_78361.1	648	111412	279	53109	0.0827	1.107147	8/2	55	0.37443
HL_48039.2	18489	93571	8664	44724	0.0832	1.019987	4/1	56	0.37443
HL_50312.1	4336	107724	1991	51397	0.0844	1.039068	6/1	57	0.37443
HL_66877.1	168	111892	65	53323	0.0860	1.231691	11/4	58	0.37496
HL_42345.1	1101	110959	487	52901	0.0890	1.077851	7/1	59	0.38150
HL_38138.1	838	111222	367	53021	0.0930	1.088514	6/1	60	0.39216
HL_48254.1	1398	110662	625	52763	0.0954	1.066492	9/2	61	0.39569
HL_05113.1	89	111971	32	53356	0.1000	1.325357	7/1	62	0.40813
HL_58601.1	3786	108274	1739	51649	0.1021	1.038531	5/2	63	0.40992
HL_91872.1	477	111583	205	53183	0.1154	1.109016	9/3	64	0.45619
HL_73972.1	948	111112	421	52967	0.1195	1.073421	6/3	65	0.46073
HL_27397.1	370	111690	157	53231	0.1202	1.123182	7/2	66	0.46073
HL_44522.1	172	111888	69	53319	0.1266	1.187878	5/2	67	0.47796
HL_23182.1	1189	110871	534	52854	0.1326	1.061451	8/2	68	0.49350
HL_30731.1	1609	110451	730	52658	0.1398	1.050818	4/1	69	0.51248
HL_84888.1	461	111599	200	53188	0.1429	1.098558	9/3	70	0.51637
HL_78507.1	2115	109945	968	52420	0.1528	1.041727	5/1	71	0.54096
HL_97270.3	2762	109298	1271	52117	0.1540	1.036184	7/2	72	0.54096
HL_90579.3	456	111604	199	53189	0.1603	1.092076	10/2	73	0.55558
HL_52116.1	769	111291	344	53044	0.1731	1.065476	7/2	74	0.59125
HL_33875.1	695	111365	310	53078	0.1753	1.068534	11/3	75	0.59125
HL_93530.1	1039	111021	470	52918	0.1818	1.053697	7/1	76	0.60513
HL_55202.1	13	112047	3	53385	0.1891	2.064616	13/2	77	0.62142
HL_66880.1	66	111994	25	53363	0.1940	1.257874	11/1	78	0.62916
HL_18156.2	264	111796	114	53274	0.2057	1.103536	7/2	79	0.65888
HL_24473.3	4	112056	0	53388	0.2104	Inf	13/4	80	0.66555
HL_33451.1	7	112053	1	53387	0.2131	3.334967	11/1	81	0.66559
HL_93567.1	1805	110255	833	52555	0.2284	1.032847	7/1	82	0.70473
HL_53015.1	552	111508	248	53140	0.2329	1.060724	8/3	83	0.70980
HL_17008.1	29	112031	10	53378	0.2410	1.381755	10/2	84	0.71472
HL_66174.1	995	111065	455	52933	0.2427	1.042216	9/4	85	0.71472
HL_53789.1	2093	109967	970	52418	0.2429	1.028555	6/1	86	0.71472
HL_08203.1	54	112006	21	53367	0.2550	1.225172	9/4	87	0.74158
HL_63304.1	14	112046	4	53384	0.2611	1.66756	15/1	88	0.75077
HL_20028.1	169	111891	73	53315	0.2655	1.103104	8/1	89	0.75461
HL_57923.1	247	111813	109	53279	0.2723	1.079775	7/2	90	0.76543
HL_65347.1	539	111521	245	53143	0.2843	1.048363	10/3	91	0.79028

Continued on next page

Table G – continued from previous page

Model	A	B	C	D	P-value	Odds ratio	Nuc/Bp	Rank	Adj. p-value
HL_82294.3	3034	109026	1420	51968	0.2936	1.018435	7/2	92	0.80736
HL_62970.1	248	111812	111	53277	0.3135	1.064583	13/4	93	0.85293
HL_74686.1	254	111806	114	53274	0.3195	1.061643	10/2	94	0.85566
HL_25175.1	8	112052	2	53386	0.3246	1.905751	16/4	95	0.85566
HL_68081.3	1578	110482	736	52652	0.3247	1.021785	8/2	96	0.85566
HL_87223.1	143	111917	63	53325	0.3313	1.081507	7/1	97	0.85770
HL_44390.1	15	112045	5	53383	0.3332	1.429339	11/3	98	0.85770
HL_15603.2	84	111976	36	53352	0.3356	1.111734	12/1	99	0.85770
HL_96915.1	625	111435	289	53099	0.3512	1.030528	9/2	100	0.88174
HL_13707.2	68	111992	29	53359	0.3520	1.117197	8/3	101	0.88174
HL_23195.1	5	112055	1	53387	0.3725	2.382167	17/3	102	0.91943
HL_12706.1	1649	110411	774	52614	0.3743	1.015248	4/2	103	0.91943
HL_42094.1	634	111426	296	53092	0.4016	1.020578	5/2	104	0.97403
HL_82288.1	585	111475	273	53115	0.4042	1.021032	6/1	105	0.97403
HL_33640.1	609	111451	287	53101	0.4552	1.011011	8/1	106	1.00000
HL_42436.1	2	112058	0	53388	0.4588	Inf	15/3	107	1.00000
HL_11547.2	311	111749	146	53242	0.4637	1.014896	8/1	108	1.00000
HL_93263.1	342	111718	161	53227	0.4713	1.012071	2/1	109	1.00000
HL_78731.1	855	111205	405	52983	0.4752	1.005828	9/1	110	1.00000
HL_62967.1	9566	102494	4554	48834	0.4865	1.000836	5/3	111	1.00000
HL_83865.1	5409	106651	2576	50812	0.4990	1.000373	6/1	112	1.00000
HL_20920.1	8	112052	3	53385	0.5032	1.270442	11/3	113	1.00000
HL_79902.1	209	111851	99	53289	0.5083	1.005795	8/3	114	1.00000
HL_93771.1	2827	109233	1347	52041	0.5088	0.9998779	6/1	115	1.00000
HL_74465.5	863	111197	411	52977	0.5109	1.000376	9/1	116	1.00000
HL_66948.1	489	111571	233	53155	0.5184	0.9998779	7/2	117	1.00000
HL_72543.2	313	111747	149	53239	0.5190	1.000811	10/3	118	1.00000
HL_57217.1	434	111626	207	53181	0.5242	0.9988561	8/2	119	1.00000
HL_27429.3	216	111844	103	53285	0.5297	0.9990735	9/1	120	1.00000
HL_11376.1	24425	87635	11647	41741	0.5383	0.9988626	5/1	121	1.00000
HL_65249.1	8829	103231	4214	49174	0.5446	0.9980271	6/2	122	1.00000
HL_28676.1	1344	110716	643	52745	0.5457	0.9957704	6/1	123	1.00000
HL_94578.1	367	111693	176	53212	0.5490	0.9934294	6/3	124	1.00000
HL_86398.1	87	111973	42	53346	0.5696	0.9868666	11/3	125	1.00000
HL_11509.2	423	111637	204	53184	0.5757	0.9878318	10/3	126	1.00000
HL_24108.1	72	111988	35	53353	0.5842	0.9800446	8/2	127	1.00000
HL_42969.1	23	112037	11	53377	0.5849	0.9961571	10/1	128	1.00000
HL_24544.2	2164	109896	1039	52349	0.5904	0.9921291	8/3	129	1.00000
HL_01926.3	1082	110978	521	52867	0.5910	0.9893211	8/3	130	1.00000
HL_55718.1	8303	103757	3973	49415	0.5970	0.9953081	3/1	131	1.00000
HL_21545.1	2198	109862	1056	52332	0.5976	0.9914792	6/1	132	1.00000
HL_62880.1	3043	109017	1461	51927	0.6041	0.9920892	6/1	133	1.00000
HL_50059.1	642	111418	312	53076	0.6282	0.9802061	7/4	134	1.00000
HL_06997.2	16	112044	8	53380	0.6376	0.9528407	12/2	135	1.00000
HL_68733.1	202	111858	100	53288	0.6487	0.9623217	8/1	136	1.00000
HL_38897.1	118	111942	59	53329	0.6523	0.9527978	7/1	137	1.00000
HL_32644.2	359	111701	177	53211	0.6644	0.96622	10/3	138	1.00000
HL_20806.1	1	112059	0	53388	0.6773	Inf	11/2	139	1.00000
HL_45358.1	1	112059	0	53388	0.6773	Inf	13/2	140	1.00000
HL_98577.1	1	112059	0	53388	0.6773	Inf	13/2	141	1.00000
HL_85044.1	21	112039	11	53377	0.6777	0.9095222	11/2	142	1.00000
HL_94697.1	143	111917	72	53316	0.6781	0.9461621	11/2	143	1.00000
HL_46175.2	490	111570	242	53146	0.6922	0.9645232	10/2	144	1.00000
HL_85534.1	340	111720	169	53219	0.6925	0.9583632	7/3	145	1.00000
HL_76585.1	469	111591	232	53156	0.6961	0.9629755	9/2	146	1.00000
HL_13786.1	2736	109324	1325	52063	0.6962	0.9833538	5/1	147	1.00000
HL_84289.1	32	112028	17	53371	0.7015	0.8967725	11/2	148	1.00000
HL_88960.1	405	111655	202	53186	0.7190	0.9550434	8/2	149	1.00000
HL_29196.1	591	111469	293	53095	0.7249	0.9607807	6/1	150	1.00000

Continued on next page

Table G – continued from previous page

Model	A	B	C	D	P-value	Odds ratio	Nuc/Bp	Rank	Adj. p-value
HL_19226.1	515	111545	256	53132	0.7251	0.9582423	5/1	151	1.00000
HL_82538.1	21764	90296	10437	42951	0.7323	0.9918979	5/1	152	1.00000
HL_46570.1	958	111102	473	52915	0.7480	0.9646512	8/1	153	1.00000
HL_59182.1	2	112058	1	53387	0.7548	0.9528466	15/2	154	1.00000
HL_54751.2	61	111999	33	53355	0.7597	0.8806036	11/2	155	1.00000
HL_87844.2	2401	109659	1172	52216	0.7605	0.9754724	6/2	156	1.00000
HL_17537.1	177	111883	92	53296	0.7725	0.9164682	8/1	157	1.00000
HL_60200.1	1102	110958	546	52842	0.7824	0.9612027	8/2	158	1.00000
HL_04194.1	331	111729	170	53218	0.8015	0.927412	7/3	159	1.00000
HL_58223.1	168	111892	89	53299	0.8103	0.8991696	5/1	160	1.00000
HL_23129.1	51	112009	29	53359	0.8118	0.837789	9/2	161	1.00000
HL_19528.1	512	111548	261	53127	0.8240	0.9342944	9/3	162	1.00000
HL_26495.1	368	111692	190	53198	0.8283	0.9225048	8/1	163	1.00000
HL_07915.1	564	111496	287	53101	0.8285	0.9359257	6/1	164	1.00000
HL_76679.1	3591	108469	1758	51630	0.8327	0.9722585	5/2	165	1.00000
HL_96994.1	10	112050	7	53381	0.8518	0.6805647	11/1	166	1.00000
HL_58083.2	254	111806	135	53253	0.8604	0.8961514	9/2	167	1.00000
HL_88311.2	558	111502	287	53101	0.8628	0.9259199	7/3	168	1.00000
HL_85018.1	82	111978	47	53341	0.8653	0.8310994	10/1	169	1.00000
HL_62881.1	503	111557	260	53128	0.8661	0.9213455	10/4	170	1.00000
HL_95049.1	680	111380	348	53040	0.8690	0.9305225	8/1	171	1.00000
HL_64371.2	28	112032	18	53370	0.8746	0.741015	12/2	172	1.00000
HL_47784.2	636	111424	327	53061	0.8763	0.9262049	11/2	173	1.00000
HL_39895.6	5528	106532	2704	50684	0.8777	0.9726158	5/2	174	1.00000
HL_39486.1	4233	107827	2079	51309	0.8793	0.9688868	7/1	175	1.00000
HL_23290.1	1177	110883	594	52794	0.8801	0.943431	7/1	176	1.00000
HL_91613.1	14	112046	10	53378	0.8840	0.6669413	10/3	177	1.00000
HL_45411.1	688	111372	354	53034	0.8873	0.9254748	8/2	178	1.00000
HL_67761.1	4145	107915	2039	51349	0.8886	0.9673181	6/1	179	1.00000
HL_72498.12	801	111259	411	52977	0.8955	0.9279913	7/2	180	1.00000
HL_77692.1	603	111457	314	53074	0.9056	0.9144578	10/4	181	1.00000
HL_25195.1	3	112057	3	53385	0.9100	0.4764001	12/2	182	1.00000
HL_42553.1	16	112044	12	53376	0.9168	0.6351717	10/3	183	1.00000
HL_91226.3	1422	110638	722	52666	0.9226	0.9375385	8/1	184	1.00000
HL_82243.1	1205	110855	616	52772	0.9269	0.9312268	9/1	185	1.00000
HL_70912.1	599	111461	316	53072	0.9334	0.9025772	7/1	186	1.00000
HL_84768.1	514	111546	274	53114	0.9381	0.8932443	4/1	187	1.00000
HL_80492.2	66	111994	42	53346	0.9406	0.7484921	8/4	188	1.00000
HL_69403.1	176	111884	102	53286	0.9486	0.8218022	9/3	189	1.00000
HL_68697.2	12287	99773	5997	47391	0.9488	0.973161	6/1	190	1.00000
HL_18781.4	1906	110154	969	52419	0.9532	0.9360269	8/2	191	1.00000
HL_30366.1	255	111805	144	53244	0.9533	0.8433235	11/5	192	1.00000
HL_38898.1	332	111728	184	53204	0.9542	0.859227	8/1	193	1.00000
HL_33402.3	1623	110437	830	52558	0.9545	0.9306054	7/2	194	1.00000
HL_65924.1	2	112058	3	53385	0.9598	0.3176058	14/4	195	1.00000
HL_49492.1	4	112056	5	53383	0.9638	0.3811177	14/4	196	1.00000
HL_35200.1	1569	110491	808	52580	0.9661	0.9240732	6/1	197	1.00000
HL_39872.2	643	111417	347	53041	0.9714	0.8821556	8/2	198	1.00000
HL_80459.3	15085	96975	7373	46015	0.9739	0.9707987	5/1	199	1.00000
HL_19132.1	318	111742	181	53207	0.9744	0.8365824	8/3	200	1.00000
HL_56775.1	605	111455	329	53059	0.9751	0.8754331	7/2	201	1.00000
HL_16770.1	166	111894	101	53287	0.9767	0.7827382	10/2	202	1.00000
HL_66467.1	2367	109693	1215	52173	0.9841	0.9265949	5/1	203	1.00000
HL_38130.2	24	112036	21	53367	0.9851	0.5443609	11/1	204	1.00000
HL_65802.1	241	111819	144	53244	0.9857	0.7969348	11/3	205	1.00000
HL_78228.1	677	111383	372	53016	0.9874	0.8662406	12/2	206	1.00000
HL_39942.2	879	111181	478	52910	0.9907	0.8751291	7/1	207	1.00000
HL_60203.1	161	111899	103	53285	0.9913	0.7443092	5/2	208	1.00000
HL_86123.2	958	111102	519	52869	0.9914	0.8783773	10/2	209	1.00000

Continued on next page

Table G – continued from previous page

Model	A	B	C	D	P-value	Odds ratio	Nuc/Bp	Rank	Adj. p-value
HL_41833.2	51	112009	40	53348	0.9927	0.6072537	11/4	210	1.00000
HL_64543.2	1488	110572	793	52595	0.9950	0.8925483	9/2	211	1.00000
HL_39422.1	537	111523	308	53080	0.9956	0.8298483	7/1	212	1.00000
HL_08382.2	15585	96475	7681	45707	0.9957	0.9613123	6/1	213	1.00000
HL_52574.3	4511	107549	2298	51090	0.9962	0.9325092	7/1	214	1.00000
HL_61547.5	16828	95232	8292	45096	0.9968	0.9610274	6/1	215	1.00000
HL_49036.3	24351	87709	11922	41466	0.9971	0.9656693	6/2	216	1.00000
HL_06122.1	1678	110382	897	52491	0.9975	0.8895882	7/3	217	1.00000
HL_47337.1	4700	107360	2402	50986	0.9979	0.9292535	5/1	218	1.00000
HL_48480.1	635	111425	365	53023	0.9980	0.8278877	7/1	219	1.00000
HL_68579.1	363	111697	221	53167	0.9981	0.7818626	6/2	220	1.00000
HL_62228.3	6782	105278	3427	49961	0.9981	0.9391561	6/2	221	1.00000
HL_34440.1	2133	109927	1130	52258	0.9982	0.8973528	6/1	222	1.00000
HL_99633.1	530	111530	314	53074	0.9990	0.803246	8/3	223	1.00000
HL_59610.1	1688	110372	916	52472	0.9993	0.8760917	5/1	224	1.00000
HL_98233.1	85	111975	69	53319	0.9996	0.5865761	8/1	225	1.00000
HL_37962.1	237	111823	159	53229	0.9996	0.7095096	8/2	226	1.00000
HL_49210.1	716	111344	420	52968	0.9997	0.8110014	7/2	227	1.00000
HL_99779.1	4649	107411	2444	50944	1.0000	0.9022039	5/1	228	1.00000
HL_27271.2	22948	89112	11421	41967	1.0000	0.9462652	6/1	229	1.00000
HL_55272.1	2318	109742	1283	52105	1.0000	0.8578252	10/4	230	1.00000
HL_67000.1	6568	105492	3426	49962	1.0000	0.9079623	5/2	231	1.00000
HL_52540.1	4859	107201	2575	50813	1.0000	0.8944338	4/2	232	1.00000
HL_56824.2	11825	100235	6029	47359	1.0000	0.9267017	5/1	233	1.00000
HL_91693.1	11389	100671	5826	47562	1.0000	0.9235744	5/1	234	1.00000
HL_95720.1	1057	111003	639	52749	1.0000	0.786083	8/3	235	1.00000
HL_10116.1	0	112060	0	53388	1.0000	0	18/5	236	1.00000
HL_19221.2	6475	105585	3607	49781	1.0000	0.8463725	6/2	237	1.00000
HL_19399.1	0	112060	0	53388	1.0000	0	16/2	238	1.00000
HL_19452.1	0	112060	2	53386	1.0000	0	11/3	239	1.00000
HL_25124.2	4485	107575	2493	50895	1.0000	0.8511577	5/2	240	1.00000
HL_25197.1	0	112060	1	53387	1.0000	0	15/4	241	1.00000
HL_29831.1	7144	104916	3899	49489	1.0000	0.8642908	6/2	242	1.00000
HL_57843.1	3532	108528	1966	51422	1.0000	0.8512369	5/2	243	1.00000
HL_67042.12	14869	97191	7801	45587	1.0000	0.8940232	6/2	244	1.00000
HL_70420.2	0	112060	0	53388	1.0000	0	14/8	245	1.00000
HL_76036.3	12018	100042	6499	46889	1.0000	0.8667196	5/1	246	1.00000
HL_76766.4	8830	103230	4796	48592	1.0000	0.8666522	5/2	247	1.00000
HL_79038.1	10571	101489	5684	47704	1.0000	0.8741815	5/1	248	1.00000
HL_84353.1	9347	102713	5037	48351	1.0000	0.8735429	6/2	249	1.00000
HL_87136.2	1276	110784	821	52567	1.0000	0.7374463	8/1	250	1.00000
HL_90102.1	0	112060	0	53388	1.0000	0	19/3	251	1.00000
HL_97784.1	0	112060	2	53386	1.0000	0	15/1	252	1.00000
HL_97971.1	6645	105415	3721	49667	1.0000	0.8414104	6/1	253	1.00000

Table H: Results of Fisher's exact test according to scheme shown in Table B for JAR3D HL module predictions from all JAR3D HL models with mean interior edit distance ≤ 4 and passed cutoff ≥ 50.

order	model	A	B	C	D	p.value	odds.ratio
H	all	94208	17852	44755	8633	0.108449	1.017947

Table I: False discovery rates (FDR) for individual mRm_{IL} models with mean RMDetect score $\geq Q_{0.75}$ in windows with p-score > 0.9 and $0.25 \leq$ GC content ≤ 0.75 of order H. Status "+" denotes models with adjusted p-value ≤ 0.05 and odds ratio ≥ 1.0 whereas "*" denotes models with odds ratio ≥ 1.0. Models assigned with "-" are neither enriched nor occur at higher rates in the original data. Colored rows have an FDR $\leq \hat{F}_{rnaz}$ and original window count ≥ 6. The table is sorted in ascending order according to the FDR.

**p-score > 0.9, 0.25 ≤ GC content ≤ 0.75**					
ModelID	# original	# shuffled	FDR (%)	Odds ratio	Stat.
$\hat{F}_{rnaz}$	142 455	66 891	47.0%		
RF00011_1583_1NBS_177_190_209_219	0	0	0.000	0.000	-
RF00177_1133_2VHO_380_389_400_408	0	0	0.000	0.000	-
RF00177_698_2B64_481_491_518_522	0	0	0.000	Inf	*
RF01118_270_2QBC_13_17_35_55	0	0	0.000	0.000	-
RF00177_351_1I95_804_807_829_846	1	0	0.000	Inf	*
RF00177_437_2QBF_488_495_500_509	0	0	0.000	0.000	-
RF00177_551_2UXD_494_500_507_515	0	0	0.000	0.000	-
RF00177_171_1HR0_804_807_829_846	1	0	0.000	Inf	*
RF00177_1470_1XNQ_1103_1111_1121_1128	1	0	0.000	0.476	-
RF00177_1369_2GY9_669_675_686_695	2	0	0.000	Inf	*
RF00177_524_1VOV_400_408_417_424	3	0	0.000	Inf	*
RF00177_857_3BBN_130_136_151_158	12	2	16.667	1.429	*
RF00177_1012_3FIH_120_126_141_148	12	2	16.667	1.143	*
RF00177_942_1N36_137_143_158_165	11	2	18.182	1.310	*
RF00177_243_2QBB_382_389_400_406	5	1	20.000	0.596	-
RF00177_751_1IBL_660_665_676_684	4	1	25.000	3.335	*
RF00177_235_2HHH_1380_1383_1471_1475	172	47	27.326	1.746	+
RF01857_613_1QZW_192_198_207_213	14	4	28.571	1.112	*
RF00177_831_2V48_435_440_463_469	269	86	31.970	1.312	+
RF00177_1312_2HGR_59_65_89_93	55	18	32.727	0.907	-
RF00177_206_2Z4M_419_423_456_461	1507	497	32.979	1.381	+
RF00177_1124_2VHP_419_427_452_461	6	2	33.333	1.270	*
RF00177_1_1J5E_482_490_519_521	9	3	33.333	0.681	-
RF00177_325_1VS5_121_126_141_147	52	18	34.615	0.986	-
RF00177_1595_1N34_1100_1107_1122_1131	8	3	37.500	1.588	*
RF00177_1_2GYB_490_498_527_529	8	3	37.500	0.953	-
RF00177_955_2HHH_436_440_463_468	644	243	37.733	1.278	+
RF00177_874_2F4V_1225_1231_1262_1268	392	149	38.010	1.308	+
RF00167_327_1Y26_26_29_49_56	324	124	38.272	1.217	*
RF00177_145_2QBF_1222_1228_1259_1265	398	153	38.442	1.331	+
RF00001_5_1N8R_75_81_101_106	876	353	40.297	1.135	*
RF00177_32_1VOX_748_752_768_772	487	198	40.657	1.132	*
RF00162_600_2GIS_17_21_32_39	637	259	40.659	1.064	*
RF00177_11_2VHP_752_756_772_776	481	196	40.748	1.107	*
RF00177_35_2B9M_757_761_777_781	516	211	40.891	1.133	*
RF00001_1334_2GYC_21_28_52_58	1327	547	41.221	1.128	*
RF00001_196_2QBE_69_77_95_103	2335	965	41.328	1.097	*
RF00015_758_2OZB_28_34_42_45	1892	791	41.808	1.070	*
RF00177_418_1S1H_661_665_676_683	42	18	42.857	1.132	*
RF00177_231_2GYB_766_770_786_790	519	224	43.160	1.032	*

Continued on next page

Table I – continued from previous page

p-score > 0.9, 0.25 ≤ GC content ≤ 0.75					
ModelID	# original	# shuffled	FDR (%)	Odds ratio	Stat.
$\hat{F}_{rnaz}$	142 455	66 891	47.0%		
RF00177_1166_2UXD_1224_1231_1262_1269	221	96	43.439	1.082	*
RF00177_90_1HNW_1281_1285_1308_1312	154	67	43.506	1.170	*
RF00001_449_3CCQ_32_37_43_48	3099	1371	44.240	1.071	*
RF00177_1114_2VHP_1404_1410_1441_1446	857	385	44.924	1.052	*
RF00177_284_2QBJ_1099_1104_1118_1124	225	102	45.333	1.112	*
RF00001_1060_3BBO_68_77_96_105	693	318	45.887	1.094	*
RF00177_1303_2HHH_138_143_158_164	53	25	47.170	1.035	*
RF00177_192_2QBD_657_661_672_679	38	18	47.368	1.202	*
RF00017_631_2J37_181_187_212_216	2396	1163	48.539	1.009	*
RF00177_1127_2VHO_1404_1411_1440_1446	205	102	49.756	1.016	*
RF00177_324_1VS5_475_484_513_516	2	1	50.000	1.429	*
RF00177_1592_1N36_436_445_457_468	2	1	50.000	0.715	-
RF00017_1608_1L9A_190_194_205_209	193	97	50.259	0.970	-
RF00177_579_2J02_440_446_456_463	664	343	51.657	0.888	-
RF00059_318_2GDI_55_59_72_76	4740	2558	53.966	0.862	-
RF00177_244_3DF3_1278_1282_1305_1309	112	61	54.464	0.960	-
RF00177_432_2QP0_215_220_250_257	29	18	62.069	0.802	-
RF00177_36_2QB9_862_869_880_885	12	8	66.667	0.953	-
RF00177_334_1HNW_233_238_268_275	23	16	69.565	0.673	-
RF00169_236_1CQ5_37_41_52_56	75	57	76.000	0.786	-
RF00177_87_2QAN_541_548_855_857	7	6	85.714	0.298	-
RF00177_495_2B64_859_866_877_882	10	9	90.000	0.749	-
RF00177_1167_2UXD_399_407_418_425	1	3	300.000	0.159	-

Table J: False discovery rates (FDR) for individual JAR3D IL modules with mean interior edit distance ≤ 4 and passed cutoff ≥ 50 in windows with p-score > 0.9 and 0.25 ≤ GC content ≤ 0.75 of order H. Status "+" denotes models with adjusted p-value ≤ 0.05 and odds ratio ≥ 1.0 whereas "*" denotes models with odds ratio ≥ 1.0. Models assigned with "-" are neither enriched nor occur at higher rates in the original data. "Msl" = average 3D sequence length. Colored rows have an FDR ≤ $\hat{F}_{rnaz}$, original window count ≥ 6, and Msl ≥9. The table is sorted in ascending order according to FDR.

p-score > 0.9, 0.25 ≤ GC content ≤ 0.75						
ModelID	# original	# shuffled	FDR (%)	Odds ratio	Stat.	Msl
$\hat{F}_{rnaz}$	142 455	66 891	47.0%			
IL_05513.1	2	0	0.000	Inf	*	12
IL_39324.1	0	0	0.000	0.000	-	20
IL_40527.1	0	0	0.000	0.000	-	16
IL_42891.1	1	0	0.000	Inf	*	16
IL_46435.1	7	0	0.000	Inf	*	13
IL_52610.1	0	0	0.000	0.000	-	37
IL_76095.3	0	0	0.000	0.000	-	20

Continued on next page

Table J – continued from previous page

**p-score > 0.9, 0.25 ≤ GC content ≤ 0.75**						
ModelID	# original	# shuffled	FDR (%)	Odds ratio	Stat.	Msl
$\hat{F}_{rnaz}$	142 455	66 891	47.0%			
IL_76263.1	0	0	0.000	0.000	-	19
IL_77076.1	0	0	0.000	0.000	-	18
IL_77296.1	0	0	0.000	0.000	-	15
IL_06847.1	6	0	0.000	Inf	*	13
IL_88367.1	0	0	0.000	0.000	-	15
IL_89028.6	0	0	0.000	0.000	-	16
IL_90057.1	0	0	0.000	0.000	-	25
IL_94744.1	2	0	0.000	Inf	*	17
IL_97842.1	0	0	0.000	0.000	-	16
IL_98655.1	0	0	0.000	0.000	-	16
IL_02359.3	0	0	0.000	0.000	-	17.6
IL_16330.1	0	0	0.000	0.000	-	28
IL_21421.1	0	0	0.000	0.000	-	20
IL_21495.1	1	0	0.000	0.000	-	15
IL_23414.1	0	0	0.000	0.000	-	21
IL_25082.1	2	0	0.000	Inf	*	18
IL_25230.3	0	0	0.000	0.000	-	18
IL_27668.1	2	0	0.000	Inf	*	12
IL_28572.2	7	0	0.000	Inf	*	16
IL_33964.1	0	0	0.000	0.000	-	23
IL_80505.1	10	1	10.000	4.289	*	11
IL_34628.2	84	11	13.095	2.859	+	13
IL_39526.4	7	1	14.286	2.859	*	9
IL_98591.3	7	1	14.286	2.859	*	16
IL_63133.1	57	9	15.789	3.098	+	6
IL_23448.1	91	16	17.582	3.117	+	6
IL_61730.1	28	5	17.857	2.382	*	11
IL_21639.1	50	10	20.000	2.277	*	9
IL_41791.1	99	20	20.202	1.756	*	12
IL_86357.3	82	17	20.732	2.264	+	6
IL_34363.2	23	5	21.739	2.263	*	14
IL_96206.3	320	72	22.500	2.211	+	6
IL_73000.2	277	63	22.744	2.208	+	6.57
IL_54450.1	48	11	22.917	1.776	*	8
IL_57364.1	26	6	23.077	2.382	*	8
IL_28644.1	164	38	23.171	2.196	+	6
IL_06177.1	43	10	23.256	1.906	*	6
IL_58291.4	131	31	23.664	2.307	+	6
IL_91089.1	50	12	24.000	2.859	+	8

Continued on next page

Table J – continued from previous page

p-score > 0.9, 0.25 ≤ GC content ≤ 0.75						
ModelID	# original	# shuffled	FDR (%)	Odds ratio	Stat.	Msl
$\hat{F}_{rnaz}$	142 455	66 891	47.0%			
IL_69799.1	124	30	24.194	2.193	+	6
IL_43877.1	95	23	24.211	1.803	+	7.5
IL_71942.1	12	3	25.000	1.270	*	10
IL_75415.1	8	2	25.000	0.953	-	10
IL_77014.1	4	1	25.000	Inf	*	11
IL_85647.3	16	4	25.000	1.787	*	15.06
IL_31066.3	44	11	25.000	1.906	*	7
IL_73276.5	212	54	25.472	1.875	+	8.18
IL_70173.1	186	48	25.806	1.875	+	7.5
IL_92027.3	170	44	25.882	1.933	+	6.24
IL_92027.3	170	44	25.882	1.933	+	6.24
IL_37406.1	99	26	26.263	1.798	+	8.5
IL_87065.1	148	39	26.351	1.668	+	7
IL_11751.1	45	12	26.667	1.473	*	7
IL_98421.4	123	33	26.829	1.643	+	7
IL_67887.1	231	62	26.840	1.777	+	6.5
IL_58586.2	334	90	26.946	1.790	+	7
IL_98566.1	100	27	27.000	1.525	*	9
IL_28947.2	344	93	27.035	1.828	+	5.45
IL_41153.1	33	9	27.273	1.482	*	8
IL_39980.1	120	33	27.500	1.765	+	8
IL_40845.1	207	57	27.536	1.688	+	8
IL_97217.11	373	103	27.614	1.742	+	5.14
IL_06808.1	101	28	27.723	1.525	*	9
IL_43946.1	18	5	27.778	1.548	*	12
IL_99397.1	18	5	27.778	1.429	*	9
IL_31039.1	68	19	27.941	1.710	*	7
IL_37325.1	50	14	28.000	1.906	*	7
IL_44540.4	221	62	28.054	1.725	+	5.3
IL_39199.4	284	80	28.169	1.722	+	5.28
IL_47875.1	567	160	28.219	1.756	+	7.67
IL_92109.3	576	166	28.819	1.645	+	6.07
IL_46648.6	239	69	28.870	1.661	+	5.62
IL_25300.3	307	89	28.990	1.649	+	5.29
IL_94430.5	386	113	29.275	1.623	+	5.94
IL_56513.1	116	34	29.310	1.459	*	9
IL_50911.1	37	11	29.730	1.800	*	8
IL_56465.4	514	153	29.767	1.539	+	5.74
IL_50521.1	60	18	30.000	1.373	*	15

Continued on next page

Table J – continued from previous page

p-score > 0.9, 0.25 ≤ GC content ≤ 0.75						
ModelID	# original	# shuffled	FDR (%)	Odds ratio	Stat.	Msl
$\hat{F}_{rnaz}$	142 455	66 891	47.0%			
IL_91379.1	10	3	30.000	1.906	*	12
IL_25271.2	30	9	30.000	2.144	*	15
IL_31663.1	113	34	30.088	1.430	*	7.5
IL_15205.1	149	45	30.201	1.430	*	13
IL_68767.1	109	33	30.275	1.702	+	6
IL_63253.1	102	31	30.392	1.735	+	7
IL_09491.1	36	11	30.556	1.477	*	8
IL_82444.1	193	59	30.570	1.405	*	6.59
IL_01080.1	221	68	30.769	1.613	+	8
IL_13069.3	94	29	30.851	1.298	*	8
IL_17212.2	238	74	31.092	1.390	+	6.73
IL_20775.1	67	21	31.343	1.407	*	8
IL_86059.1	118	37	31.356	1.545	*	7
IL_21254.1	51	16	31.373	1.293	*	14
IL_88119.1	206	65	31.553	1.486	+	8
IL_47732.1	22	7	31.818	1.225	*	9
IL_02809.3	287	92	32.056	1.603	+	9.11
IL_41139.1	62	20	32.258	1.354	*	8
IL_18675.1	102	33	32.353	1.553	*	7
IL_17066.1	70	23	32.857	1.347	*	6
IL_31006.1	219	72	32.877	1.333	*	12
IL_80348.3	191	63	32.984	1.480	+	6.33
IL_47174.11	300	99	33.000	1.385	+	6.2
IL_37104.3	229	76	33.188	1.346	*	7.36
IL_37347.1	18	6	33.333	1.334	*	16
IL_86981.1	3	1	33.333	1.429	*	15
IL_95150.6	78	26	33.333	1.596	*	8.33
IL_35043.1	6	2	33.333	1.429	*	11
IL_13777.1	119	40	33.613	1.430	*	5
IL_12147.1	217	73	33.641	1.416	+	6.5
IL_21333.2	287	97	33.798	1.474	+	9.5
IL_42251.1	213	72	33.803	1.416	+	7
IL_16166.4	138	47	34.058	1.377	*	5
IL_71685.1	93	32	34.409	1.175	*	14
IL_57785.5	55	19	34.545	1.345	*	9
IL_11778.1	40	14	35.000	1.283	*	8
IL_69106.1	207	73	35.266	1.292	*	8.5
IL_87394.1	34	12	35.294	1.032	*	10
IL_90459.3	544	192	35.294	1.290	+	7.56

Continued on next page

Table J – continued from previous page

p-score > 0.9, 0.25 ≤ GC content ≤ 0.75						
ModelID	# original	# shuffled	FDR (%)	Odds ratio	Stat.	Msl
$\hat{F}_{rnaz}$	142 455	66 891	47.0%			
IL_90133.3	161	57	35.404	1.268	*	8
IL_88865.1	31	11	35.484	1.429	*	9
IL_80494.2	596	213	35.738	1.252	+	12.67
IL_13959.4	176	63	35.795	1.311	*	8.23
IL_23262.4	528	189	35.795	1.363	+	11
IL_46721.1	103	37	35.922	1.566	*	11
IL_30381.1	72	26	36.111	1.367	*	8.5
IL_39355.1	412	150	36.408	1.231	*	9
IL_79083.3	793	291	36.696	1.275	+	11
IL_97296.1	241	89	36.929	1.215	*	8.5
IL_63952.1	615	228	37.073	1.253	+	13
IL_06421.1	121	45	37.190	1.105	*	8
IL_65553.8	137	51	37.226	1.342	*	13.3
IL_22909.1	115	43	37.391	1.229	*	9
IL_55649.1	457	171	37.418	1.218	*	9
IL_55938.4	334	126	37.725	1.245	*	7.56
IL_87507.1	254	96	37.795	1.105	*	9.5
IL_43316.1	1002	380	37.924	1.203	*	11
IL_43124.2	195	74	37.949	1.145	*	7.09
IL_47758.2	126	48	38.095	1.166	*	8.5
IL_70401.1	13	5	38.462	1.191	*	11
IL_08926.3	111	43	38.739	1.191	*	5.5
IL_12211.1	152	59	38.816	1.288	*	13.5
IL_83856.1	649	252	38.829	1.255	+	12
IL_64589.1	64	25	39.062	1.293	*	8
IL_87904.5	1373	538	39.184	1.198	*	10
IL_77263.2	362	142	39.227	1.127	*	11
IL_06471.1	598	235	39.298	1.154	*	10
IL_74876.2	1017	401	39.430	1.216	*	10.67
IL_05684.1	256	101	39.453	1.208	*	9
IL_41766.6	375	148	39.467	1.178	*	8.26
IL_00998.1	111	44	39.640	1.249	*	8
IL_85510.1	873	348	39.863	1.128	*	11
IL_82650.1	15	6	40.000	0.794	-	13
IL_37197.1	808	324	40.099	1.164	*	11
IL_26971.1	1019	409	40.137	1.163	*	11
IL_15840.2	868	350	40.323	1.125	*	11
IL_24982.5	1602	650	40.574	1.155	*	10
IL_83920.1	64	26	40.625	1.086	*	7

Continued on next page

Table J – continued from previous page

p-score > 0.9, 0.25 ≤ GC content ≤ 0.75						
ModelID	# original	# shuffled	FDR (%)	Odds ratio	Stat.	Msl
$\hat{F}_{rnaz}$	142 455	66 891	47.0%			
IL_93568.2	243	99	40.741	1.125	*	12.5
IL_06211.3	153	63	41.176	1.182	*	9.33
IL_97191.1	17	7	41.176	1.112	*	13
IL_57977.1	82	34	41.463	0.953	-	11
IL_80093.1	750	311	41.467	1.143	*	11
IL_70237.3	1101	459	41.689	1.133	*	10
IL_80714.1	561	234	41.711	1.144	*	12
IL_31224.1	366	153	41.803	1.050	*	10
IL_39585.1	1219	511	41.920	1.125	*	12
IL_97509.1	876	369	42.123	1.077	*	10.5
IL_93424.4	747	315	42.169	1.086	*	10.19
IL_22732.1	201	85	42.289	1.069	*	9
IL_87548.1	513	217	42.300	1.108	*	11.5
IL_45794.1	52	22	42.308	0.953	-	14
IL_24546.4	163	69	42.331	1.142	*	10
IL_11302.1	1075	457	42.512	1.040	*	10
IL_09587.1	3336	1419	42.536	1.077	*	11.5
IL_07300.2	148	63	42.568	1.103	*	9.33
IL_05723.1	570	243	42.632	1.145	*	10
IL_79955.2	438	187	42.694	1.127	*	12.67
IL_70299.1	96	41	42.708	1.149	*	14
IL_47444.3	934	399	42.719	1.050	*	12
IL_25380.1	194	83	42.784	1.107	*	8.33
IL_53635.3	119	51	42.857	1.210	*	13
IL_78513.1	28	12	42.857	0.780	-	10
IL_52509.1	629	270	42.925	1.003	*	10
IL_21077.1	204	88	43.137	1.010	*	10
IL_86336.1	361	156	43.213	1.014	*	10
IL_46306.1	1422	616	43.319	1.045	*	10
IL_69536.1	1313	569	43.336	1.103	*	10
IL_75328.1	23	10	43.478	1.361	*	14
IL_09333.1	374	164	43.850	1.060	*	13
IL_06468.1	319	140	43.887	1.057	*	11
IL_45262.4	170	75	44.118	1.026	*	9.25
IL_16415.2	18	8	44.444	0.834	-	15
IL_23639.1	1407	626	44.492	1.039	*	10
IL_91044.1	409	182	44.499	1.046	*	11
IL_54966.1	336	150	44.643	1.026	*	9
IL_09530.1	792	354	44.697	1.035	*	11.5

Continued on next page

Table J – continued from previous page

p-score > 0.9, 0.25 ≤ GC content ≤ 0.75						
ModelID	# original	# shuffled	FDR (%)	Odds ratio	Stat.	Msl
$\hat{F}_{rnaz}$	142 455	66 891	47.0%			
IL_46034.3	96	43	44.792	0.965	-	9
IL_31555.5	732	328	44.809	1.039	*	9.23
IL_33842.1	29	13	44.828	1.032	*	9
IL_78744.1	24	11	45.833	1.429	*	12
IL_17603.1	408	187	45.833	1.134	*	12
IL_06180.1	1093	507	46.386	1.005	*	10.67
IL_24293.1	84	39	46.429	0.926	-	8.5
IL_52958.1	3150	1470	46.667	1.006	*	10
IL_82188.2	272	127	46.691	0.957	-	14
IL_54470.1	429	201	46.853	0.938	-	9
IL_58454.1	678	318	46.903	1.010	*	10
IL_59529.1	586	275	46.928	1.035	*	11
IL_49493.4	136	64	47.059	0.935	-	13
IL_03110.1	446	211	47.309	0.994	-	13
IL_82563.1	57	27	47.368	0.931	-	10
IL_52173.1	189	90	47.619	0.983	-	9
IL_30067.1	328	157	47.866	1.022	*	10
IL_26868.1	465	224	48.172	0.963	-	11
IL_92321.4	576	278	48.264	0.959	-	12.14
IL_97057.3	826	399	48.305	1.002	*	11.83
IL_48918.3	93	45	48.387	0.940	-	13
IL_46301.1	287	139	48.432	0.957	-	9.43
IL_81398.1	3827	1861	48.628	0.972	-	10
IL_12507.1	92	45	48.913	0.929	-	13
IL_96446.1	145	71	48.966	0.875	-	11
IL_53988.1	118	58	49.153	0.896	-	13
IL_28003.1	65	32	49.231	1.038	*	8.67
IL_62499.7	562	278	49.466	0.909	-	11
IL_98924.1	491	243	49.491	0.882	-	10
IL_49527.1	897	445	49.610	0.976	-	10
IL_03282.1	141	70	49.645	0.909	-	10
IL_87523.1	161	80	49.689	0.938	-	11
IL_38807.3	70	35	50.000	1.044	*	12.75
IL_06306.1	36	18	50.000	1.209	*	14
IL_65137.1	108	54	50.000	0.922	-	13
IL_80652.1	18	9	50.000	1.021	*	14
IL_82601.1	2	1	50.000	Inf	*	8
IL_94403.5	2	1	50.000	Inf	*	12.75
IL_02835.1	4	2	50.000	1.906	*	13

Continued on next page

Table J – continued from previous page

**p-score > 0.9, 0.25 ≤ GC content ≤ 0.75**						
ModelID	# original	# shuffled	FDR (%)	Odds ratio	Stat.	Msl
$\hat{F}_{rnaz}$	142 455	66 891	47.0%			
IL_27640.1	2	1	50.000	0.953	-	15
IL_85805.1	1103	555	50.317	0.956	-	9
IL_66744.1	93	47	50.538	0.860	-	14
IL_30840.2	2790	1411	50.573	0.930	-	11.33
IL_53323.1	1176	597	50.765	0.891	-	10
IL_46990.1	2070	1051	50.773	0.939	-	12.5
IL_40892.1	121	62	51.240	1.061	*	11
IL_83250.1	147	76	51.701	0.998	-	11
IL_72158.3	160	83	51.875	0.865	-	13
IL_37053.1	290	151	52.069	0.920	-	13.67
IL_09882.1	124	65	52.419	1.027	*	12
IL_95652.3	38	20	52.632	0.826	-	14
IL_25181.1	1239	673	54.318	0.891	-	13
IL_49976.1	1249	693	55.484	0.836	-	13
IL_25307.1	66	37	56.061	0.749	-	9
IL_91078.1	14	8	57.143	0.953	-	14
IL_31754.1	7	4	57.143	0.715	-	13
IL_59934.1	94	55	58.511	0.849	-	13
IL_89794.1	29	17	58.621	0.851	-	14
IL_92114.3	75	44	58.667	0.843	-	9
IL_60649.1	51	30	58.824	0.812	-	13
IL_47687.1	283	168	59.364	0.809	-	14
IL_52940.1	268	167	62.313	0.834	-	15.5
IL_44067.4	16	10	62.500	0.774	-	14
IL_97833.1	30	19	63.333	0.916	-	14
IL_54954.1	3	2	66.667	1.429	*	17
IL_76486.1	3	2	66.667	0.715	-	15
IL_31707.1	12	8	66.667	0.635	-	11
IL_94973.1	17	12	70.588	0.520	-	14
IL_68827.1	36	27	75.000	0.538	-	11
IL_05221.1	4	3	75.000	0.476	-	17
IL_17682.1	1	1	100.000	0.000	-	17
IL_90735.1	20	21	105.000	0.603	-	17
IL_27243.1	2	3	150.000	0.238	-	15
IL_57285.1	0	1	Inf	0.000	-	13
IL_91904.1	0	1	Inf	0.000	-	17

Table K: False discovery rates (FDR) for individual JAR3D HL models with mean interior edit distance ≤ 4 and passed cutoff ≥ 50 in windows with p-score > 0.9 and $0.25 \leq$ GC content ≤ 0.75 of order H. Status "+" denotes models with adjusted p-value ≤ 0.05 and odds ratio ≥ 1.0 whereas "*" denotes models with odds ratio ≥ 1.0. Models assigned with "-" are neither enriched nor occur at higher rates in the original data. Colored rows have an FDR $\leq \hat{F}_{rnaz}$ and original window count ≥ 6. The table is sorted in ascending order according to the FDR.

**p-score > 0.9, 0.25 ≤ GC content ≤ 0.75**					
ModelID	# original	# shuffled	FDR (%)	Odds ratio	Stat.
$\hat{F}_{rnaz}$	142 455	66 891	47.0%		
HL_45358.1	1	0	0.000	Inf	*
HL_10116.1	0	0	0.000	0.000	-
HL_70420.2	1	0	0.000	0.000	-
HL_90102.1	0	0	0.000	0.000	-
HL_19399.1	0	0	0.000	0.000	-
HL_24473.3	5	0	0.000	Inf	*
HL_42436.1	2	0	0.000	Inf	*
HL_33451.1	10	1	10.000	3.335	*
HL_73465.1	36	5	13.889	3.455	*
HL_44467.1	51	11	21.569	1.776	*
HL_44390.1	26	6	23.077	1.429	*
HL_20920.1	12	3	25.000	1.270	*
HL_25175.1	8	2	25.000	1.906	*
HL_55202.1	15	4	26.667	2.065	*
HL_56809.1	120	36	30.000	1.370	*
HL_87223.1	215	73	33.953	1.082	*
HL_05361.1	732	253	34.563	1.384	+
HL_65071.1	1258	463	36.804	1.253	+
HL_87554.1	2394	888	37.093	1.283	+
HL_90542.1	1225	455	37.143	1.359	+
HL_26579.1	859	320	37.253	1.214	*
HL_66877.1	206	78	37.864	1.232	*
HL_98833.3	1024	388	37.891	1.128	*
HL_68435.1	1136	437	38.468	1.167	*
HL_13707.2	96	37	38.542	1.117	*
HL_75759.4	7308	2838	38.834	1.208	*
HL_05113.1	102	40	39.216	1.325	*
HL_97499.1	2589	1018	39.320	1.205	*
HL_30008.1	2606	1025	39.332	1.199	*
HL_08203.1	61	24	39.344	1.225	*
HL_45018.3	1113	438	39.353	1.167	*

Continued on next page

Table K – continued from previous page

**p-score > 0.9, 0.25 ≤ GC content ≤ 0.75**					
ModelID	# original	# shuffled	FDR (%)	Odds ratio	S.tat
$\hat{F}_{rnaz}$	142 455	66 891	47.0%		
HL_10378.1	2082	821	39.433	1.234	*
HL_00721.1	1804	713	39.523	1.145	*
HL_74559.1	3507	1392	39.692	1.182	*
HL_35865.1	7475	2972	39.759	1.169	*
HL_23195.1	5	2	40.000	2.382	*
HL_17723.4	2326	937	40.284	1.137	*
HL_06643.3	6967	2807	40.290	1.157	*
HL_44522.1	218	88	40.367	1.188	*
HL_17790.1	2837	1150	40.536	1.125	*
HL_59604.1	2832	1149	40.572	1.158	*
HL_78420.1	693	282	40.693	1.141	*
HL_59225.1	2659	1084	40.767	1.137	*
HL_24711.1	934	381	40.792	1.110	*
HL_15603.2	110	45	40.909	1.112	*
HL_42677.2	5809	2390	41.143	1.133	*
HL_63304.1	17	7	41.176	1.668	*
HL_17008.1	34	14	41.176	1.382	*
HL_46489.2	1197	493	41.186	1.130	*
HL_35619.2	1911	788	41.235	1.137	*
HL_66880.1	80	33	41.250	1.258	*
HL_27429.3	295	122	41.356	0.999	-
HL_01418.1	1951	808	41.415	1.103	*
HL_06997.2	24	10	41.667	0.953	-
HL_74505.1	5475	2283	41.699	1.120	*
HL_72273.1	972	406	41.770	1.149	*
HL_63941.1	5691	2391	42.014	1.118	*
HL_41827.1	1548	657	42.442	1.101	*
HL_24707.1	2190	930	42.466	1.094	*
HL_20028.1	209	89	42.584	1.103	*
HL_33640.1	836	356	42.584	1.011	*
HL_18587.1	1032	440	42.636	1.154	*
HL_33277.1	2043	872	42.682	1.091	*
HL_99584.1	1668	712	42.686	1.127	*
HL_27397.1	459	196	42.702	1.123	*
HL_75850.1	4867	2083	42.798	1.109	*
HL_78361.1	800	343	42.875	1.107	*
HL_99207.1	2250	968	43.022	1.161	*
HL_00090.1	920	397	43.152	1.161	*
HL_65347.1	672	290	43.155	1.048	*

Continued on next page

Table K – continued from previous page

p-score > 0.9, 0.25 ≤ GC content ≤ 0.75					
ModelID	# original	# shuffled	FDR (%)	Odds ratio	S.tat
$\hat{F}_{rnaz}$	142 455	66 891	47.0%		
HL_86115.1	5087	2202	43.287	1.093	*
HL_52116.1	1002	434	43.313	1.065	*
HL_34027.2	9093	3939	43.319	1.079	*
HL_03785.1	4699	2036	43.328	1.088	*
HL_42969.1	30	13	43.333	0.996	-
HL_57923.1	323	140	43.344	1.080	*
HL_73972.1	1164	505	43.385	1.073	*
HL_90579.3	578	252	43.599	1.092	*
HL_91872.1	588	257	43.707	1.109	*
HL_48254.1	1727	755	43.717	1.066	*
HL_53789.1	2725	1195	43.853	1.029	*
HL_53015.1	724	318	43.923	1.061	*
HL_94578.1	505	222	43.960	0.993	-
HL_30731.1	2002	885	44.206	1.051	*
HL_30128.1	1100	490	44.545	1.089	*
HL_50312.1	5418	2414	44.555	1.039	*
HL_94697.1	193	86	44.560	0.946	-
HL_82288.1	726	324	44.628	1.021	*
HL_36842.1	2508	1123	44.777	1.070	*
HL_12706.1	2056	923	44.893	1.015	*
HL_21675.3	9386	4214	44.897	1.031	*
HL_93567.1	2345	1053	44.904	1.033	*
HL_33875.1	844	379	44.905	1.069	*
HL_96915.1	786	353	44.911	1.031	*
HL_68081.3	1985	892	44.937	1.022	*
HL_38138.1	1099	494	44.950	1.089	*
HL_42345.1	1335	601	45.019	1.078	*
HL_97270.3	3457	1558	45.068	1.036	*
HL_74686.1	335	151	45.075	1.062	*
HL_66174.1	1231	556	45.167	1.042	*
HL_42077.2	18422	8321	45.169	1.038	*
HL_75579.2	19690	8899	45.196	1.041	*
HL_34108.2	12294	5559	45.217	1.032	*
HL_23182.1	1476	668	45.257	1.061	*
HL_58601.1	4700	2132	45.362	1.039	*
HL_57514.2	11499	5219	45.387	1.062	*
HL_62970.1	304	138	45.395	1.065	*
HL_18156.2	337	153	45.401	1.104	*
HL_48116.2	11115	5047	45.407	1.049	*

Continued on next page

Table K – continued from previous page

p-score > 0.9, 0.25 ≤ GC content ≤ 0.75					
ModelID	# original	# shuffled	FDR (%)	Odds ratio	S.tat
$\hat{F}_{rnaz}$	142 455	66 891	47.0%		
HL_74465.5	1109	504	45.446	1.000	*
HL_93530.1	1234	561	45.462	1.054	*
HL_29196.1	782	356	45.524	0.961	-
HL_78507.1	2552	1163	45.572	1.042	*
HL_57217.1	555	253	45.586	0.999	-
HL_84888.1	565	258	45.664	1.099	*
HL_98523.2	5979	2731	45.677	1.053	*
HL_42094.1	817	376	46.022	1.021	*
HL_85044.1	26	12	46.154	0.910	-
HL_19905.3	9824	4537	46.183	1.043	*
HL_13786.1	3577	1652	46.184	0.983	-
HL_65249.1	11035	5100	46.217	0.998	-
HL_48039.2	22713	10524	46.335	1.020	*
HL_95716.1	7014	3269	46.607	1.036	*
HL_11509.2	541	253	46.765	0.988	-
HL_82538.1	26668	12472	46.768	0.992	-
HL_11376.1	29990	14026	46.769	0.999	-
HL_62967.1	11945	5598	46.865	1.001	*
HL_86398.1	98	46	46.939	0.987	-
HL_62880.1	3920	1849	47.168	0.992	-
HL_87844.2	2954	1399	47.360	0.975	-
HL_68697.2	15236	7222	47.401	0.973	-
HL_76679.1	4542	2163	47.622	0.972	-
HL_78731.1	1098	523	47.632	1.006	*
HL_72543.2	384	183	47.656	1.001	*
HL_93771.1	3562	1699	47.698	1.000	-
HL_82294.3	3650	1741	47.699	1.018	*
HL_58083.2	354	169	47.740	0.896	-
HL_28676.1	1732	828	47.806	0.996	-
HL_21545.1	2785	1334	47.899	0.991	-
HL_80459.3	18599	8913	47.922	0.971	-
HL_50059.1	824	395	47.937	0.980	-
HL_79902.1	256	123	48.047	1.006	*
HL_49036.3	29777	14309	48.054	0.966	-
HL_08382.2	19060	9160	48.059	0.961	-
HL_55718.1	10309	4960	48.113	0.995	-
HL_39895.6	7029	3382	48.115	0.973	-
HL_01926.3	1321	636	48.145	0.989	-
HL_34440.1	2884	1389	48.162	0.897	-

Continued on next page

Table K – continued from previous page

**p-score > 0.9, 0.25 ≤ GC content ≤ 0.75**					
ModelID	# original	# shuffled	FDR (%)	Odds ratio	S.tat
$\hat{F}_{rnaz}$	142 455	66 891	47.0%		
HL_23290.1	1474	711	48.236	0.943	-
HL_83865.1	6524	3147	48.237	1.000	*
HL_66467.1	3055	1480	48.445	0.927	-
HL_39486.1	5420	2627	48.469	0.969	-
HL_45411.1	885	429	48.475	0.925	-
HL_19226.1	649	315	48.536	0.958	-
HL_61547.5	20371	9907	48.633	0.961	-
HL_77692.1	777	378	48.649	0.914	-
HL_66948.1	600	292	48.667	1.000	-
HL_27271.2	28295	13779	48.698	0.946	-
HL_67761.1	5165	2523	48.848	0.967	-
HL_95049.1	863	422	48.899	0.931	-
HL_32644.2	433	212	48.961	0.966	-
HL_56824.2	14649	7184	49.041	0.927	-
HL_46570.1	1204	591	49.086	0.965	-
HL_62228.3	8208	4033	49.135	0.939	-
HL_91693.1	14009	6889	49.176	0.924	-
HL_07915.1	679	334	49.190	0.936	-
HL_58223.1	241	119	49.378	0.899	-
HL_19528.1	654	325	49.694	0.934	-
HL_35200.1	1974	981	49.696	0.924	-
HL_24544.2	2593	1289	49.711	0.992	-
HL_46175.2	599	298	49.750	0.965	-
HL_30366.1	337	168	49.852	0.843	-
HL_11547.2	399	199	49.875	1.015	*
HL_18781.4	2361	1179	49.936	0.936	-
HL_47337.1	5527	2763	49.991	0.929	-
HL_62881.1	626	313	50.000	0.921	-
HL_64371.2	36	18	50.000	0.741	-
HL_98577.1	2	1	50.000	Inf	*
HL_91226.3	1757	881	50.142	0.938	-
HL_68733.1	251	126	50.199	0.962	-
HL_88311.2	699	352	50.358	0.926	-
HL_59610.1	2179	1098	50.390	0.876	-
HL_60200.1	1344	681	50.670	0.961	-
HL_47784.2	792	402	50.758	0.926	-
HL_52574.3	5577	2834	50.816	0.933	-
HL_72498.12	972	494	50.823	0.928	-
HL_76585.1	598	304	50.836	0.963	-

Continued on next page

Table K – continued from previous page

**p-score > 0.9, 0.25 ≤ GC content ≤ 0.75**					
ModelID	# original	# shuffled	FDR (%)	Odds ratio	S.tat
$\hat{F}_{rnaz}$	142 455	66 891	47.0%		
HL_70912.1	777	395	50.837	0.903	-
HL_67042.12	18345	9327	50.842	0.894	-
HL_67000.1	7829	3988	50.939	0.908	-
HL_82243.1	1436	732	50.975	0.931	-
HL_33402.3	2019	1033	51.164	0.931	-
HL_84289.1	37	19	51.351	0.897	-
HL_93263.1	406	209	51.478	1.012	*
HL_64543.2	1886	971	51.485	0.893	-
HL_85534.1	402	207	51.493	0.958	-
HL_84353.1	11504	5938	51.617	0.874	-
HL_26495.1	455	235	51.648	0.923	-
HL_04194.1	385	199	51.688	0.927	-
HL_84768.1	729	377	51.715	0.893	-
HL_39872.2	810	419	51.728	0.882	-
HL_88960.1	491	254	51.731	0.955	-
HL_06122.1	2125	1103	51.906	0.890	-
HL_52540.1	5754	2989	51.946	0.894	-
HL_17537.1	221	115	52.036	0.916	-
HL_23129.1	69	36	52.174	0.838	-
HL_39942.2	1105	577	52.217	0.875	-
HL_86123.2	1247	654	52.446	0.878	-
HL_76766.4	10801	5666	52.458	0.867	-
HL_38898.1	436	229	52.523	0.859	-
HL_76036.3	14634	7690	52.549	0.867	-
HL_79038.1	12725	6687	52.550	0.874	-
HL_25124.2	5637	2977	52.812	0.851	-
HL_56775.1	744	393	52.823	0.875	-
HL_29831.1	8663	4586	52.938	0.864	-
HL_38897.1	166	88	53.012	0.953	-
HL_99779.1	5553	2949	53.106	0.902	-
HL_96994.1	13	7	53.846	0.681	-
HL_19221.2	8063	4344	53.876	0.846	-
HL_97971.1	8064	4363	54.105	0.841	-
HL_39422.1	667	366	54.873	0.830	-
HL_60203.1	225	124	55.111	0.744	-
HL_48480.1	788	435	55.203	0.828	-
HL_24108.1	81	45	55.556	0.980	-
HL_49210.1	938	522	55.650	0.811	-
HL_65802.1	312	174	55.769	0.797	-

Continued on next page

Table K – continued from previous page

p-score > 0.9, 0.25 ≤ GC content ≤ 0.75					
ModelID	# original	# shuffled	FDR (%)	Odds ratio	S.tat
$\hat{F}_{rnaz}$	142 455	66 891	47.0%		
HL_57843.1	4213	2356	55.922	0.851	-
HL_55272.1	2800	1579	56.393	0.858	-
HL_78228.1	820	465	56.707	0.866	-
HL_95720.1	1316	764	58.055	0.786	-
HL_80492.2	93	54	58.065	0.748	-
HL_68579.1	467	275	58.887	0.782	-
HL_54751.2	77	46	59.740	0.881	-
HL_85018.1	97	58	59.794	0.831	-
HL_16770.1	214	128	59.813	0.783	-
HL_69403.1	207	125	60.386	0.822	-
HL_37962.1	298	180	60.403	0.710	-
HL_99633.1	646	391	60.526	0.803	-
HL_19132.1	367	229	62.398	0.837	-
HL_87136.2	1574	990	62.897	0.737	-
HL_38130.2	36	23	63.889	0.544	-
HL_91613.1	15	10	66.667	0.667	-
HL_41833.2	66	45	68.182	0.607	-
HL_42553.1	19	14	73.684	0.635	-
HL_98233.1	122	91	74.590	0.587	-
HL_65924.1	4	3	75.000	0.318	-
HL_59182.1	3	3	100.000	0.953	-
HL_20806.1	1	1	100.000	Inf	*
HL_25195.1	3	3	100.000	0.476	-
HL_49492.1	4	5	125.000	0.381	-
HL_97784.1	0	3	Inf	0.000	-
HL_25197.1	0	1	Inf	0.000	-
HL_19452.1	0	2	Inf	0.000	-
