# Supplementary material for: RNA 3D Modules in Genome-Wide Predictions of RNA 2D Structure
Source: PLoS One. 2015 Oct 28;10(10):e0139900. doi: 10.1371/journal.pone.0139900 (PMC4624896; doi:10.1371/journal.pone.0139900)
Supplement: S2 Text — The file contains the occurrence rates (Table A), number of significantly enriched modules (Table B), the number of modules that lower the false discovery rate by at least 10% and at least 20% (Table C), and false discovery rates of the different 3D module prediction methods (Table D) for the alternative approaches of ordering L and R. (PDF) [file pone.0139900.s006.pdf]

Supplementary Information 2
Alternative approaches L and R.

Counting the number of 3D module predictions per window is complicated by the fact that the original windows overlap to varying degrees and some 3D modules are found in the overlapping regions. We remove overlapping original windows ($0.9 <$ RNAz p-score ≤ 1.0) by listing them from lowest p-score to highest or in random order. We call these orderings L and R. After obtaining a list of sorted windows, we then go through the list and dismiss any original windows that overlap with a window that appears earlier in the list. This reduces the number of original windows by ∼11.7% but eliminates double-counting difficulties. No such procedure is needed for shuffled windows since they are shuffled independently and thus do not overlap one another.

To address whether 3D modules (M_{mrmIL}, M_{jarIL} and M_{jarHL}) occur more frequently in windows with RNAz p-score>0.9 we consider if 3D modules occur in the original and shuffled windows at the same nominal rate. So as a background we assume that there would be a 50% chance of seeing a higher rate in the original windows, and the number of modules with a higher rate in the original windows would have a binomial distribution with probability 0.5. The 3D modules that were not even found once in either the original or shuffled windows were discarded. Table A shows that M_{mrmIL} and M_{jarIL} occur at higher rates in original than in shuffled windows whereas M_{jarHL} do not occur at a higher rate in original windows (ordering L) and only at a slightly higher rate in original windows of order R.

Table A: Occurrence rate of M_{mrmIL}, M_{jarIL}, and M_{jarHL}. First column shows the ordering of the windows with H=high to low , L=low to high and R=random order of RNAz p-scores. "Succ./Size" denotes the success rate, i.e. the number of 3D module predictions with odds ratio > 1.0, and the total sample size. Furthermore, the z-score ("z") and the p-values are presented.

	M_{mrmIL}			M_{jarIL}			M_{jarHL}		
**Order**	**Succ./Size**	**z**	**p-value**	**Succ./Size**	**z**	**p-value**	**Succ./Size**	**z**	**p-value**
H	43/58	3.6	$1.0*10^{-4}$	116/173	4.5	$2.0*10^{-6}$	119/246	-0.5	$4.5*10^{-2}$
L	40/58	2.9	$1.6*10^{-3}$	116/173	4.5	$2.0*10^{-6}$	119/245	-0.4	$4.6*10^{-2}$
R	40/58	2.9	$1.6*10^{-3}$	124/173	5.7	$3.8*10^{-2}$	130/245	1.0	$3.2*10^{-2}$

To address whether there are 3D modules which more often generated in RNAz

predictions on original than shuffled windows, we simply conduct a (one-sided Fisher's exact) enrichment test (FET) and correct for multiple testing using the Benjamini-Hochberg procedure at a 5% false discovery rate. We refer to this test as $\mathrm{FET}_{fixpscore_{al}}$ in what follows, where we count windows matching at least one 3D module and windows without 3D module predictions. For mRm_{IL} predictions we increase the list of top 25% highest scoring candidates (see main paper) to the top 40% as we otherwise get low counts of modules for many models.

Table B: Number and percentage (in parenthesis) of 3D models that are significantly enriched in Fisher's exact test $\mathrm{FET}_{fixpscore}$ with an adjusted p-value ≤ 0.05 after multiple testing correction at 5% false discovery rate. I_S shows the number (%) of distinct models that are enriched in all orderings (intersection of FET results of ordering H, L, and R).

	Order	# mRm_{IL} mod.	# JAR3D IL mod.	# JAR3D HL mod.
$\mathrm{FET}_{fixpscore}$	H	8 (12.7)	13 (4.7)	28 (11.1)
	L	12 (19.0)	12 (4.3)	29 (11.5)
	R	14 (22.2)	22 (8.0)	41 (16.2)
I_S	{H,L,R}	8 (12.7)	12 (19.0)	27 (10.7)

We evaluate the potential of 3D module predictions to lower the FDR in RNAz predictions (p-score>0.9) in the GC content range of 0.25 to 0.75. We get 142 490 RNAz candidates in the original windows with order L, and 142 475 candidates in the original windows with order R. For the shuffled windows we get 66 891 candidates. This corresponds to $\hat{F}_{rnaz}$ of 46.9%. Since not all models perform equally well we estimate the FDR for each model individually. We accept windows with at least one M_{mrmIL}, M_{jarIL}, or M_{jarHL}. Table C summarizes the results.

Table C: Number of modules that lower the false discovery rate by at least 10% and at least 20%.

**Order**	**H** 10%/20%	**L** 10%/20%	**R** 10%/20%
	# modules	# modules	# modules
mRm_{IL}	6/3	6/6	7/7
JAR3D IL	8/7	7/6	15/13
JAR3D HL	4/4	3/3	5/5

For the estimation of the 2D/3D FDRs for all models of one type together we accept windows that match at least one M_{mrmIL}, M_{jarIL}, or M_{jarHL} made by a

model with adjusted p-value ≤ 0.05 and an odds ratio ≥ 1.25, because we want to include only the best performing modules. Furthermore, M_{jarIL} have a sequence length $\geq$9. We refer to the FDRs of the different methods as $\hat{F}_{mrmIL}$, $\hat{F}_{jarIL}$, $\hat{F}_{jarHL}$ and for a combination of the methods as $\hat{F}_{mrmIL\&jarHL}$, $\hat{F}_{mrmIL\&jarIL}$, $\hat{F}_{jarIL\&jarHL}$. For the combined methods we require that a window matches at least one module of each method.

Table D: False discovery rates for RNAz (p-score > 0.9, $0.25 \leq$ GC content ≤ 0.75) and 3D module predictions of order L and R. Only predictions of modules that are enriched and have an odds ratio ≥ 1.25 are taken into account. Furthermore, JAR3D IL modules have a sequence length ≥ 9. Subscripts denote which method for module prediction is used. "$\text{win}_{shu}/\text{win}_{ori}$" shows the number of shuffled and original windows that match a 3D module.

	FDR	FDR	FDR
$\hat{F}_{rnaz}$	47.0%	46.9%	46.9%
Order	**H**	**L**	**R**
$\hat{F}_{mrmIL}$<br>$\text{win}_{shu}/\text{win}_{ori}$	34.1%<br>815/2390	33.3%<br>815/2448	33.2%<br>937/2820
$\hat{F}_{jarIL}$<br>$\text{win}_{shu}/\text{win}_{ori}$	36.0%<br>1224/3401	35.7%<br>1093/3059	36.3%<br>2091/5754
$\hat{F}_{jarHL}$<br>$\text{win}_{shu}/\text{win}_{ori}$	37.0%<br>2020/5465	36.5%<br>1588/4345	36.8%<br>2220/6040
$\hat{F}_{mRmIL\&jarIL}$<br>$\text{win}_{shu}/\text{win}_{ori}$	27.8%<br>25/90	27.6%<br>21/76	26.8%<br>40/149
$\hat{F}_{mRmIL\&jarHL}$<br>$\text{win}_{shu}/\text{win}_{ori}$	24.8%<br>25/101	23.0%<br>20/87	27.8%<br>32/115
$\hat{F}_{jarIL\&jarHL}$<br>$\text{win}_{shu}/\text{win}_{ori}$	26.4%<br>37/140	25.0%<br>27/108	28.7%<br>62/216
