# Supplementary material for: RNA 3D Modules in Genome-Wide Predictions of RNA 2D Structure
Source: PLoS One. 2015 Oct 28;10(10):e0139900. doi: 10.1371/journal.pone.0139900 (PMC4624896; doi:10.1371/journal.pone.0139900)
Supplement: S3 Text — Fisher’s exact test results for individual mRm modules of ordering L (Table A) and of ordering R (Table B), all mRm modules of ordering L and R (Table C), individual JAR3D IL modules of ordering L (Table D) and of ordering R (Table E), all JAR3D IL modules of ordering L and R (Table F), individual JAR3D HL modules of ordering L (Table G) and of ordering R (Table H), and all JAR3D HL modules of ordering L and R (Table I). (PDF) [file pone.0139900.s007.pdf]

Supplementary Information 3
Fisher's exact test results of alternative approaches L and R.

Table A: Fisher's exact test according to the FET scheme (see Supplementary Material S6) for mRm_{IL} module predictions for SISSIz and original data of order L. Yellow highlighted rows have a significant p-value after multiple testing correction using the Benjamini-Hochberg procedure at a 5% false discovery rate (adjusted p-value). "Nuc./Pb" denotes the total strand length and the number of paired bases.

ModelID	A	B	C	D	P-value	Odds ratio	Nuc./Pb	Rank	Adj. p-value
RF00177_206_2Z4M_419_423_456_461	1943	107620	675	52713	2.936243e-15	1.409915	11/10	1	1.84983309e-13
RF00177_235_2HHH_1380_1383_1471_1475	230	109333	62	53326	9.001215e-06	1.809349	9/6	2	0.00028
RF00177_145_2QBF_1222_1228_1259_1265	540	109023	190	53198	4.452943e-05	1.386827	14/10	3	0.00094
RF00177_955_2HHH_436_440_463_468	817	108746	310	53078	7.424164e-05	1.286305	11/7	4	0.00102
RF00177_874_2F4V_1225_1231_1262_1268	534	109029	190	53198	8.068326e-05	1.371349	14/8	5	0.00102
RF00001_1334_2GYC_21_28_52_58	1749	107814	728	52660	0.0002	1.173433	15/7	6	0.00152
RF00001_196_2QBE_69_77_95_103	2941	106622	1273	52115	0.0002	1.129222	21/16	7	0.00152
RF00001_5_1N8R_75_81_101_106	1160	108403	471	52917	0.0004	1.202217	15/12	8	0.00300
RF00177_831_2V48_435_440_463_469	357	109206	125	53263	0.0007	1.39297	13/6	9	0.00467
RF00001_449_3CCQ_32_37_43_48	3830	105733	1714	51674	0.0014	1.092075	12/8	10	0.00910
RF00015_758_2OZB_28_34_42_45	2412	107151	1058	52330	0.0020	1.113381	11/8	11	0.01135
RF00167_327_1Y26_26_29_49_56	428	109135	168	53220	0.0090	1.24232	12/6	12	0.04731
RF00177_35_2B9M_757_761_777_781	692	108871	287	53101	0.0111	1.176004	10/10	13	0.05357
RF00001_1060_3BBO_68_77_96_105	874	108689	377	53011	0.0246	1.130701	23/14	14	0.11051
RF00177_32_1VOX_748_752_768_772	603	108960	259	53129	0.0469	1.135217	10/8	15	0.19707
RF00177_1114_2VHP_1404_1410_1441_1446	1121	108442	500	52888	0.0513	1.093437	13/6	16	0.20208
RF00177_90_1HNW_1281_1285_1308_1312	200	109363	79	53309	0.0629	1.23402	10/10	17	0.23302
RF00177_11_2VHP_752_756_772_776	595	108968	260	53128	0.0752	1.115748	10/6	18	0.25353
RF00017_631_2J37_181_187_212_216	3040	106523	1415	51973	0.0765	1.04822	12/6	19	0.25353
RF00162_600_2GIS_17_21_32_39	772	108791	345	53043	0.0948	1.091019	13/11	20	0.29857
RF00177_284_2QBJ_1099_1104_1118_1124	268	109295	117	53271	0.1740	1.116445	13/9	21	0.52206
RF00177_1166_2UXD_1224_1231_1262_1269	285	109278	126	53262	0.1958	1.102446	16/8	22	0.55946
RF00177_192_2QBD_657_661_672_679	55	109508	21	53367	0.2042	1.276306	13/10	23	0.55946
RF00177_418_1S1H_661_665_676_683	59	109504	24	53364	0.2671	1.197986	13/11	24	0.63832
RF00177_751_1IBL_660_665_676_684	6	109557	1	53387	0.2740	2.923774	15/10	25	0.63832
RF01857_613_1QZW_192_198_207_213	18	109545	6	53382	0.2828	1.461916	14/6	26	0.63832
RF00177_1127_2VHO_1404_1411_1440_1446	283	109280	129	53259	0.2836	1.069174	15/6	27	0.63832
RF00177_942_1N36_137_143_158_165	13	109550	4	53384	0.2985	1.583729	15/8	28	0.63832
RF00177_857_3BBN_130_136_151_158	13	109550	4	53384	0.2985	1.583729	15/6	29	0.63832
RF00177_524_1VOV_400_408_417_424	3	109560	0	53388	0.3040	Inf	17/6	30	0.63832
RF00177_231_2GYB_766_770_786_790	640	108923	302	53086	0.3360	1.032813	10/8	31	0.68279
RF00177_244_3DF3_1278_1282_1305_1309	150	109413	70	53318	0.4133	1.044234	10/8	32	0.79271
RF00177_1012_3FIH_120_126_141_148	13	109550	5	53383	0.4325	1.266921	15/10	33	0.79271
RF00177_1369_2GY9_669_675_686_695	2	109561	0	53388	0.4521	Inf	17/8	34	0.79271
RF00177_698_2B64_481_491_518_522	2	109561	0	53388	0.4521	Inf	16/9	35	0.79271
RF00177_324_1VS5_475_484_513_516	4	109559	1	53387	0.4722	1.949152	14/9	36	0.79271
RF00177_1124_2VHP_419_427_452_461	8	109555	3	53385	0.4889	1.299487	19/8	37	0.79271
RF00177_1595_1N34_1100_1107_1122_1131	8	109555	3	53385	0.4889	1.299487	18/6	38	0.79271
RF00017_1608_1L9A_190_194_205_209	226	109337	109	53279	0.4907	1.010351	10/8	39	0.79271
RF00177_1303_2HHH_138_143_158_164	60	109503	29	53359	0.5358	1.008175	13/10	40	0.84388
RF00177_325_1VS5_121_126_141_147	59	109504	29	53359	0.5653	0.9913607	13/10	41	0.86867
RF00177_36_2QB9_862_869_880_885	14	109549	7	53381	0.6225	0.9745384	14/12	42	0.91260
RF00177_1312_2HGR_59_65_89_93	61	109502	31	53357	0.6229	0.9588287	13/11	43	0.91260
RF00177_171_1HR0_804_807_829_846	1	109562	0	53388	0.6724	Inf	22/9	44	0.91356
RF00177_1133_2VHO_380_389_400_408	1	109562	0	53388	0.6724	Inf	19/8	45	0.91356
RF00177_351_1I95_804_807_829_846	1	109562	0	53388	0.6724	Inf	22/8	46	0.91356
RF00177_495_2B64_859_866_877_882	13	109550	7	53381	0.6815	0.9049436	14/8	47	0.91356
RF00177_1_2GYB_490_498_527_529	9	109554	5	53383	0.7062	0.8771036	12/7	48	0.92684
RF00177_432_2QP0_215_220_250_257	32	109531	19	53369	0.7989	0.8206494	14/11	49	1.00000
RF00177_1_1J5E_482_490_519_521	10	109553	7	53381	0.8411	0.6960716	12/7	50	1.00000
RF00177_243_2QBB_382_389_400_406	5	109558	4	53384	0.8638	0.6090734	15/9	51	1.00000
RF00177_334_1HNW_233_238_268_275	25	109538	17	53371	0.8892	0.716505	14/10	52	1.00000
RF00177_1592_1N36_436_445_457_468	2	109561	2	53386	0.8939	0.487261	22/8	53	1.00000
RF00169_236_1CQ5_37_41_52_56	98	109465	60	53328	0.9294	0.7957331	10/6	54	1.00000
RF00177_579_2J02_440_446_456_463	850	108713	454	52934	0.9463	0.9116285	15/8	55	1.00000
RF00177_1167_2UXD_399_407_418_425	1	109562	3	53385	0.9885	0.1624236	17/6	56	1.00000
RF00177_87_2QAN_541_548_855_857	10	109553	16	53372	0.9993	0.3044897	11/7	57	1.00000
RF00011_1583_1NBS_177_190_209_219	0	109563	0	53388	1.0000	0	25/7	58	1.00000
RF01118_270_2QBC_13_17_35_55	0	109563	0	53388	1.0000	0	26/10	59	1.00000
RF00177_437_2QBF_488_495_500_509	0	109563	0	53388	1.0000	0	18/11	60	1.00000
RF00177_551_2UXD_494_500_507_515	0	109563	0	53388	1.0000	0	16/8	61	1.00000
RF00177_1470_1XNQ_1103_1111_1121_1128	0	109563	1	53387	1.0000	0	17/6	62	1.00000
RF00059_318_2GDI_55_59_72_76	5868	103695	3210	50178	1.0000	0.8845932	10/6	63	1.00000

Table B: Fisher's exact test following the FET scheme (see Supplementary Material S6) for mRm$_{IL}$ models for SISSIz and original data of order R. Yellow highlighted rows have a significant p-value after multiple testing correction using the Benjamini-Hochberg procedure at a 5% false discovery rate (adjusted p-value). "Nuc./Pb" denotes the total strand length and the number of paired bases.

ModelID	A	B	C	D	P-value	Odds ratio	Nuc./Pb	Rank	Adj. p-value
RF00177_206_2Z4M_419_423_456_461	1998	108853	675	52713	9.580057e-17	1.433402	11/10	1	6.03543591e-15
RF00177_235_2HHH_1380_1383_1471_1475	240	110611	62	53326	2.603918e-06	1.866202	9/6	2	8.2023417e-05
RF00177_955_2HHH_436_440_463_468	857	109994	310	53078	5.656643e-06	1.334064	11/7	3	0.00011
RF00177_145_2QBF_1222_1228_1259_1265	564	110287	190	53198	6.964733e-06	1.431847	14/10	4	0.00011
RF00177_874_2F4V_1225_1231_1262_1268	557	110294	190	53198	1.460138e-05	1.413994	14/8	5	0.00018
RF00001_1334_2GYC_21_28_52_58	1784	109067	728	52660	6.815661e-05	1.183163	15/7	6	0.00072
RF00001_196_2QBE_69_77_95_103	2980	107871	1273	52115	0.0001	1.130948	21/16	7	0.00123
RF00001_5_1N8R_75_81_101_106	1167	109684	471	52917	0.0006	1.195349	15/12	8	0.00435
RF00001_449_3CCQ_32_37_43_48	3886	106965	1714	51674	0.0010	1.095269	12/8	9	0.00709
RF00177_831_2V48_435_440_463_469	352	110499	125	53263	0.0016	1.357407	13/6	10	0.00969
RF00167_327_1Y26_26_29_49_56	454	110397	168	53220	0.0017	1.302807	12/6	11	0.00969
RF00015_758_2OZB_28_34_42_45	2423	108428	1058	52330	0.0036	1.105288	11/8	12	0.01906
RF00177_35_2B9M_757_761_777_781	706	110145	287	53101	0.0078	1.185917	10/10	13	0.03672
RF00177_32_1VOX_748_752_768_772	642	110209	259	53129	0.0082	1.194931	10/8	14	0.03672
RF00177_11_2VHP_752_756_772_776	633	110218	260	53128	0.0158	1.173535	10/6	15	0.06639
RF00001_1060_3BBO_68_77_96_105	881	109970	377	53011	0.0282	1.126481	23/14	16	0.11085
RF00177_90_1HNW_1281_1285_1308_1312	210	110641	79	53309	0.0333	1.280741	10/10	17	0.12344
RF00177_1114_2VHP_1404_1410_1441_1446	1140	109711	500	52888	0.0415	1.099108	13/6	18	0.14519
RF00017_631_2J37_181_187_212_216	3078	107773	1415	51973	0.0728	1.049013	12/6	19	0.24125
RF00177_1166_2UXD_1224_1231_1262_1269	304	110547	126	53262	0.0847	1.162436	16/8	20	0.26668
RF00162_600_2GIS_17_21_32_39	779	110072	345	53043	0.1017	1.088099	13/11	21	0.30502
RF00177_231_2GYB_766_770_786_790	680	110171	302	53086	0.1265	1.08496	10/8	22	0.36217
RF00177_284_2QBJ_1099_1104_1118_1124	275	110576	117	53271	0.1417	1.132333	13/9	23	0.38827
RF00177_192_2QBD_657_661_672_679	55	110796	21	53367	0.2179	1.261476	13/10	24	0.56102
RF00177_1127_2VHO_1404_1411_1440_1446	292	110559	129	53259	0.2226	1.090413	15/6	25	0.56102
RF00177_942_1N36_137_143_158_165	14	110837	4	53384	0.2542	1.68575	15/8	26	0.59320
RF00177_857_3BBN_130_136_151_158	14	110837	4	53384	0.2542	1.68575	15/6	27	0.59320
RF00177_751_1IBL_660_665_676_684	6	110845	1	53387	0.2789	2.889801	15/10	28	0.61637
RF00177_418_1S1H_661_665_676_683	59	110792	24	53364	0.2837	1.184063	13/11	29	0.61637
RF00177_244_3DF3_1278_1282_1305_1309	157	110694	70	53318	0.3228	1.080315	10/8	30	0.67795
RF00177_1012_3FIH_120_126_141_148	14	110837	5	53383	0.3808	1.348615	15/10	31	0.77393
RF01857_613_1QZW_192_198_207_213	16	110835	6	53382	0.3934	1.284313	14/6	32	0.77459
RF00177_1595_1N34_1100_1107_1122_1131	9	110842	3	53385	0.4170	1.444895	18/6	33	0.79124
RF00177_1369_2GY9_669_675_686_695	2	110849	0	53388	0.4555	Inf	17/8	34	0.79124
RF00177_524_1VOV_400_408_417_424	2	110849	0	53388	0.4555	Inf	17/6	35	0.79124
RF00177_698_2B64_481_491_518_522	2	110849	0	53388	0.4555	Inf	16/9	36	0.79124
RF00177_1303_2HHH_138_143_158_164	63	110788	29	53359	0.4692	1.046302	13/10	37	0.79124
RF00177_324_1VS5_475_484_513_516	4	110847	1	53387	0.4773	1.926504	14/9	38	0.79124
RF00177_1124_2VHP_419_427_452_461	8	110843	3	53385	0.4964	1.284293	19/8	39	0.79124
RF00177_1312_2HGR_59_65_89_93	66	110785	31	53357	0.5024	1.02542	13/11	40	0.79124
RF00017_1608_1L9A_190_194_205_209	225	110626	109	53279	0.5459	0.9941568	10/8	41	0.83457
RF00177_325_1VS5_121_126_141_147	60	110791	29	53359	0.5564	0.9964522	13/10	42	0.83457
RF00177_36_2QB9_862_869_880_885	14	110837	7	53381	0.6322	0.9632517	14/12	43	0.90470
RF00177_1_2GYB_490_498_527_529	10	110841	5	53383	0.6448	0.963253	12/7	44	0.90470
RF00177_171_1HR0_804_807_829_846	1	110850	0	53388	0.6749	Inf	22/9	45	0.90470
RF00177_1133_2VHO_380_389_400_408	1	110850	0	53388	0.6749	Inf	19/8	46	0.90470
RF00177_351_1I95_804_807_829_846	1	110850	0	53388	0.6749	Inf	22/8	47	0.90470
RF00177_432_2QP0_215_220_250_257	35	110816	19	53369	0.7179	0.8871643	14/11	48	0.94226
RF00177_495_2B64_859_866_877_882	12	110839	7	53381	0.7466	0.8256315	14/8	49	0.95991
RF00177_1_1J5E_482_490_519_521	10	110841	7	53381	0.8467	0.6879856	12/7	50	1.00000
RF00177_334_1HNW_233_238_268_275	27	110824	17	53371	0.8482	0.7648989	14/10	51	1.00000
RF00177_243_2QBB_382_389_400_406	5	110846	4	53384	0.8676	0.6019973	15/9	52	1.00000
RF00177_1592_1N36_436_445_457_468	2	110849	2	53386	0.8961	0.4816001	22/8	53	1.00000
RF00169_236_1CQ5_37_41_52_56	100	110751	60	53328	0.9229	0.8025432	10/6	54	1.00000
RF00177_579_2J02_440_446_456_463	863	109988	454	52934	0.9399	0.9148414	15/8	55	1.00000
RF00177_1167_2UXD_399_407_418_425	1	110850	3	53385	0.9888	0.1605365	17/6	56	1.00000
RF00177_87_2QAN_541_548_855_857	10	110841	16	53372	0.9994	0.3009515	11/7	57	1.00000
RF00059_318_2GDI_55_59_72_76	5955	104896	3210	50178	1.0000	0.8874299	10/6	58	1.00000
RF00011_1583_1NBS_177_190_209_219	0	110851	0	53388	1.0000	0	25/7	59	1.00000
RF01118_270_2QBC_13_17_35_55	0	110851	0	53388	1.0000	0	26/10	60	1.00000
RF00177_437_2QBF_488_495_500_509	0	110851	0	53388	1.0000	0	18/11	61	1.00000
RF00177_551_2UXD_494_500_507_515	0	110851	0	53388	1.0000	0	16/8	62	1.00000
RF00177_1470_1XNQ_1103_1111_1121_1128	0	110851	1	53387	1.0000	0	17/6	63	1.00000

Table C: Results of Fisher's exact test according to FET scheme (see Supplementary Material S6) for mRm_{IL} module predictions of all mRm_{IL} models together on SISSIz and original data of order L and R.

order	model	A	B	C	D	p.value	odds.ratio
H	all	24956	87104	11401	41987	1.312117e-05	1.055145
L	all	25000	84563	11401	41987	1.312126e-11	1.08878
R	all	25263	85588	11401	41987	2.817419e-11	1.087056

Table D: Results of Fisher's exact test according to FET scheme (see Supplementary Material S6) for JAR3D IL module predictions on SISSIz and original data of order L. Red highlighted rows have a significant p-value after multiple testing correction using the Benjamini-Hochberg procedure at a 5% false discovery rate (adjusted p-value) and average 3D sequence length ≥ 9. "Nuc./Bp" denotes the total strand length and the number of base pairs.

ModelID	A	B	C	D	P-value	Odds ratio	Nuc./Bp	Rank	Adj. p-value
IL_47875.1	528	109035	149	53239	4.223527e-10	1.730255	4/2	1	1.165693452e-07
IL_96206.3	283	109280	63	53325	9.451579e-10	2.191967	5/4	2	1.304317902e-07
IL_73000.2	246	109317	54	53334	7.193293e-09	2.222572	6/4	3	6.61782956e-07
IL_92109.3	502	109061	150	53238	2.729099e-08	1.633667	4/2	4	1.88307831e-06
IL_28947.2	318	109245	83	53305	5.78305e-08	1.86945	4/2	5	3.1922436e-06
IL_97217.11	330	109233	92	53296	4.42249e-07	1.75011	4/2	6	2.0343454e-05
IL_56465.4	467	109096	146	53242	7.53791e-07	1.56102	5/2	7	2.97209022857143e-05
IL_58586.2	282	109281	77	53311	1.45476e-06	1.786607	6/2	8	5.018922e-05
IL_39199.4	262	109301	72	53316	4.093697e-06	1.77501	5/4	9	0.00013
IL_94430.5	339	109224	102	53286	5.694159e-06	1.621412	5/3	10	0.00016
IL_23448.1	86	109477	13	53375	6.542763e-06	3.225275	6/2	11	0.00016
IL_25300.3	278	109285	81	53307	1.385853e-05	1.674103	4/2	12	0.00032
IL_58291.4	121	109442	25	53363	1.661174e-05	2.359932	6/2	13	0.00035
IL_73276.5	176	109387	44	53344	1.848308e-05	1.950645	7/5	14	0.00036
IL_28644.1	144	109419	33	53355	2.0099e-05	2.127791	5/2	15	0.00037
IL_67887.1	203	109360	55	53333	3.310894e-05	1.799987	5/3	16	0.00057
IL_70173.1	171	109392	44	53344	4.483946e-05	1.895142	6/2	17	0.00073
IL_44540.4	198	109365	55	53333	7.403848e-05	1.755572	5/4	18	0.00114
IL_69799.1	112	109451	25	53363	0.0001	2.184221	5/2	19	0.00159
IL_92027.3	146	109417	37	53351	0.0001	1.924009	6/4	20	0.00163
IL_46648.6	210	109353	62	53326	0.0002	1.651713	5/3	21	0.00259
IL_87904.5	1109	108454	444	52944	0.0002	1.219298	10/5	22	0.00259
IL_01080.1	203	109360	60	53328	0.0003	1.649834	7/2	23	0.00310
IL_02809.3	225	109338	69	53319	0.0003	1.590168	8/4	24	0.00352
IL_40845.1	170	109393	48	53340	0.0003	1.72691	7/4	25	0.00352
IL_23262.4	433	109130	154	53234	0.0003	1.371574	10/3	26	0.00370
IL_79083.3	622	108941	236	53152	0.0005	1.285847	11/4	27	0.00498
IL_86357.3	76	109487	16	53372	0.0007	2.315487	6/3	28	0.00728
IL_21333.2	238	109325	77	53311	0.0008	1.50724	8/4	29	0.00753
IL_43316.1	806	108757	320	53068	0.0009	1.228994	10/4	30	0.00836
IL_74876.2	830	108733	332	53056	0.0011	1.219841	10/4	31	0.01000
IL_24982.5	1301	108262	545	52843	0.0014	1.165164	10/5	32	0.01221
IL_63133.1	48	109515	8	53380	0.0015	2.924512	6/3	33	0.01221
IL_39980.1	102	109461	27	53361	0.0021	1.841617	6/3	34	0.01696

Continued on next page

Table D – continued from previous page

ModelID	A	B	C	D	P-value	Odds ratio	Nuc./Bp	Rank	Adj. p-value
IL_87065.1	131	109432	38	53350	0.0022	1.680647	5/3	35	0.01706
IL_83856.1	522	109041	201	53187	0.0022	1.266705	12/6	36	0.01706
IL_90459.3	463	109100	176	53212	0.0025	1.283029	6/3	37	0.01842
IL_63952.1	495	109068	191	53197	0.0030	1.264001	13/4	38	0.02206
IL_80348.3	176	109387	57	53331	0.0036	1.505396	5/2	39	0.02539
IL_47174.11	266	109297	94	53294	0.0037	1.379845	6/3	40	0.02539
IL_12147.1	196	109367	66	53322	0.0047	1.447876	6/3	41	0.03169
IL_34628.2	46	109517	9	53379	0.0051	2.491159	12/6	42	0.03367
IL_88119.1	180	109383	60	53328	0.0055	1.462601	6/3	43	0.03513
IL_98421.4	101	109462	29	53359	0.0060	1.697721	7/2	44	0.03713
IL_91089.1	39	109524	7	53381	0.0061	2.715446	8/2	45	0.03713
IL_80494.2	465	109098	182	53206	0.0062	1.245986	12/5	46	0.03713
IL_37406.1	81	109482	22	53366	0.0075	1.794662	7/2	47	0.04400
IL_43877.1	83	109480	23	53365	0.0084	1.759019	6/2	48	0.04826
IL_82444.1	171	109392	58	53330	0.0089	1.437319	6/3	49	0.05000
IL_42251.1	192	109371	67	53321	0.0097	1.397098	6/3	50	0.05357
IL_68767.1	95	109468	28	53360	0.0101	1.653838	6/2	51	0.05367
IL_21639.1	43	109520	9	53379	0.0101	2.328632	9/3	52	0.05367
IL_26971.1	834	108729	351	53037	0.0108	1.159012	10/3	53	0.05509
IL_17212.2	203	109360	72	53316	0.0108	1.374579	6/3	54	0.05509
IL_63253.1	85	109478	25	53363	0.0141	1.657261	6/2	55	0.07005
IL_37325.1	50	109513	12	53376	0.0144	2.030801	7/4	56	0.07005
IL_18675.1	90	109473	27	53361	0.0145	1.62478	7/2	57	0.07005
IL_86059.1	104	109459	33	53355	0.0172	1.536179	4/2	58	0.08064
IL_37197.1	642	108921	268	53120	0.0172	1.168265	11/4	59	0.08064
IL_46721.1	91	109472	28	53360	0.0182	1.584145	10/3	60	0.08356
IL_06808.1	82	109481	25	53363	0.0222	1.598726	8/4	61	0.09931
IL_55938.4	286	109277	111	53277	0.0223	1.256148	7/4	62	0.09931
IL_13777.1	114	109449	38	53350	0.0235	1.462322	5/2	63	0.10167
IL_16166.4	131	109432	45	53343	0.0236	1.419036	5/2	64	0.10167
IL_13959.4	157	109406	56	53332	0.0246	1.366679	8/4	65	0.10429
IL_55649.1	379	109184	153	53235	0.0262	1.207752	8/3	66	0.10948
IL_09587.1	2631	106932	1199	52189	0.0266	1.070957	11/4	67	0.10964
IL_31006.1	174	109389	64	53324	0.0297	1.325353	11/5	68	0.12065
IL_70237.3	893	108670	388	53000	0.0305	1.122493	10/4	69	0.12205
IL_39585.1	972	108591	425	52963	0.0320	1.11546	10/6	70	0.12441
IL_57364.1	26	109537	5	53383	0.0321	2.534211	8/4	71	0.12441
IL_15205.1	116	109447	40	53348	0.0331	1.413561	12/5	72	0.12441
IL_37104.3	182	109381	68	53320	0.0338	1.304747	6/3	73	0.12441
IL_41766.6	337	109226	136	53252	0.0339	1.208071	8/4	74	0.12441
IL_15840.2	686	108877	294	53094	0.0341	1.137844	11/6	75	0.12441
IL_31066.3	40	109523	10	53378	0.0343	1.949464	5/2	76	0.12441
IL_90133.3	145	109418	53	53335	0.0407	1.333607	7/3	77	0.14596
IL_95150.6	65	109498	20	53368	0.0419	1.584006	7/3	78	0.14814
IL_06471.1	468	109095	197	53191	0.0449	1.158267	10/7	79	0.15660
IL_69106.1	181	109382	69	53319	0.0456	1.278644	7/3	80	0.15660
IL_39355.1	328	109235	134	53254	0.0460	1.193308	8/3	81	0.15660
IL_06177.1	38	109525	10	53378	0.0501	1.851958	6/2	82	0.16799
IL_05684.1	223	109340	88	53300	0.0513	1.235263	8/3	83	0.16799
IL_61730.1	24	109539	5	53383	0.0514	2.339232	11/3	84	0.16799
IL_31039.1	56	109507	17	53371	0.0517	1.605467	7/3	85	0.16799
IL_31663.1	90	109473	31	53357	0.0551	1.415036	7/4	86	0.17680
IL_98566.1	75	109488	25	53363	0.0584	1.462157	8/2	87	0.18527
IL_80093.1	609	108954	264	53124	0.0591	1.124756	10/5	88	0.18535
IL_69536.1	1053	108510	471	52917	0.0631	1.090264	8/3	89	0.19566
IL_50911.1	34	109529	9	53379	0.0642	1.841095	7/3	90	0.19605
IL_56513.1	91	109472	32	53356	0.0649	1.386046	5/2	91	0.19605
IL_05723.1	461	109102	197	53191	0.0654	1.140872	10/5	92	0.19605
IL_34363.2	20	109543	4	53384	0.0664	2.436656	13/9	93	0.19708

Continued on next page

Table D – continued from previous page

ModelID	A	B	C	D	P-value	Odds ratio	Nuc./Bp	Rank	Adj. p-value
IL_54450.1	39	109524	11	53377	0.0672	1.727887	8/3	94	0.19739
IL_20775.1	63	109500	21	53367	0.0785	1.462106	7/3	95	0.22750
IL_41791.1	58	109505	19	53369	0.0797	1.487745	11/8	96	0.22750
IL_85510.1	656	108907	289	53099	0.0805	1.106712	6/2	97	0.22750
IL_80714.1	448	109115	193	53195	0.0812	1.131629	12/3	98	0.22750
IL_97509.1	703	108860	311	53077	0.0816	1.102126	10/5	99	0.22750
IL_17066.1	67	109496	23	53365	0.0873	1.419734	6/3	100	0.24106
IL_12211.1	123	109440	47	53341	0.0887	1.27549	12/6	101	0.24243
IL_06847.1	6	109557	0	53388	0.0924	Inf	11/4	102	0.24552
IL_46435.1	6	109557	0	53388	0.0924	Inf	13/2	103	0.24552
IL_50521.1	52	109511	17	53371	0.0929	1.490737	14/7	104	0.24552
IL_17603.1	334	109229	142	53246	0.0934	1.146578	11/5	105	0.24552
IL_47444.3	728	108835	326	53062	0.1072	1.088749	12/6	106	0.27729
IL_06211.3	132	109431	52	53336	0.1096	1.237199	7/2	107	0.27729
IL_22909.1	100	109463	38	53350	0.1105	1.282529	9/3	108	0.27729
IL_80505.1	9	109554	1	53387	0.1109	4.386285	11/2	109	0.27729
IL_97296.1	199	109364	82	53306	0.1111	1.182864	7/3	110	0.27729
IL_30381.1	65	109498	23	53365	0.1115	1.377346	8/4	111	0.27729
IL_25271.2	23	109540	6	53382	0.1151	1.868087	14/7	112	0.28361
IL_41139.1	55	109508	19	53369	0.1186	1.410767	7/2	113	0.28969
IL_24546.4	133	109430	53	53335	0.1217	1.223044	10/3	114	0.29467
IL_08926.3	103	109460	40	53348	0.1281	1.254952	5/3	115	0.30742
IL_65553.8	98	109465	38	53350	0.1335	1.256864	12/7	116	0.31763
IL_28572.2	5	109558	0	53388	0.1374	Inf	15/5	117	0.32140
IL_77014.1	5	109558	0	53388	0.1374	Inf	11/5	118	0.32140
IL_13069.3	77	109486	29	53359	0.1390	1.29397	4/2	119	0.32233
IL_00998.1	94	109469	37	53351	0.1563	1.23813	8/4	120	0.35867
IL_87548.1	395	109168	175	53213	0.1572	1.100221	11/6	121	0.35867
IL_64589.1	57	109506	21	53367	0.1640	1.32283	7/2	122	0.37104
IL_93424.4	587	108976	266	53122	0.1715	1.07572	9/4	123	0.38482
IL_47758.2	115	109448	47	53341	0.1755	1.192467	8/4	124	0.39059
IL_41153.1	28	109535	9	53379	0.1800	1.516114	7/3	125	0.39748
IL_79955.2	340	109223	151	53237	0.1838	1.097489	12/6	126	0.40258
IL_09491.1	30	109533	10	53378	0.1914	1.461967	7/2	127	0.41585
IL_53635.3	96	109467	39	53349	0.1935	1.199616	11/5	128	0.41722
IL_87507.1	203	109360	88	53300	0.1968	1.124293	9/4	129	0.42099
IL_43124.2	168	109395	72	53316	0.2000	1.137192	7/4	130	0.42203
IL_57785.5	46	109517	17	53371	0.2009	1.318702	8/4	131	0.42203
IL_11751.1	32	109531	11	53377	0.2018	1.417674	7/2	132	0.42203
IL_22732.1	172	109391	74	53314	0.2043	1.132801	9/5	133	0.42392
IL_31555.5	594	108969	272	53116	0.2079	1.064485	9/4	134	0.42817
IL_75328.1	22	109541	7	53381	0.2170	1.531558	14/4	135	0.44372
IL_99397.1	17	109546	5	53383	0.2225	1.656853	9/4	136	0.45145
IL_77263.2	265	109298	118	53270	0.2241	1.094544	10/5	137	0.45145
IL_46306.1	1116	108447	522	52866	0.2272	1.042199	9/3	138	0.45448
IL_88865.1	26	109537	9	53379	0.2429	1.407809	8/3	139	0.48229
IL_85647.3	14	109549	4	53384	0.2469	1.705569	15/8	140	0.48589
IL_71685.1	73	109490	30	53358	0.2499	1.185824	14/7	141	0.48589
IL_06306.1	35	109528	13	53375	0.2500	1.312056	13/2	142	0.48589
IL_93568.2	187	109376	83	53305	0.2613	1.098014	12/5	143	0.50437
IL_09333.1	303	109260	138	53250	0.2728	1.070093	12/6	144	0.52157
IL_39526.4	6	109557	1	53387	0.2740	2.923774	9/2	145	0.52157
IL_70299.1	80	109483	34	53354	0.2875	1.146642	10/4	146	0.54341
IL_07300.2	123	109440	54	53334	0.2902	1.110037	9/4	147	0.54485
IL_58454.1	576	108987	269	53119	0.2956	1.043626	9/3	148	0.54832
IL_83920.1	60	109503	25	53363	0.2970	1.169554	7/3	149	0.54832
IL_43946.1	13	109550	4	53384	0.2985	1.583729	12/6	150	0.54832
IL_38807.3	62	109501	26	53362	0.3016	1.162058	11/6	151	0.54832
IL_23639.1	1117	108446	529	52859	0.3036	1.029237	9/4	152	0.54832

Continued on next page

Table D – continued from previous page

ModelID	A	B	C	D	P-value	Odds ratio	Nuc./Bp	Rank	Adj. p-value
IL_25082.1	3	109560	0	53388	0.3040	Inf	17/6	153	0.54832
IL_09530.1	643	108920	302	53086	0.3116	1.037694	8/3	154	0.55842
IL_11778.1	33	109530	13	53375	0.3160	1.236985	7/2	155	0.56265
IL_21254.1	35	109528	14	53374	0.3233	1.218247	12/7	156	0.57194
IL_25380.1	157	109406	71	53317	0.3281	1.077619	8/4	157	0.57673
IL_78744.1	19	109544	7	53381	0.3431	1.322718	12/6	158	0.59932
IL_11302.1	812	108751	386	53002	0.3565	1.025267	10/3	159	0.61880
IL_98591.3	5	109558	1	53387	0.3625	2.43646	13/8	160	0.62535
IL_30067.1	268	109295	125	53263	0.3650	1.04484	10/5	161	0.62563
IL_37347.1	14	109549	5	53383	0.3717	1.364462	15/7	162	0.63329
IL_52173.1	171	109392	79	53309	0.3754	1.054833	9/2	163	0.63557
IL_31224.1	273	109290	128	53260	0.3816	1.039364	7/2	164	0.64221
IL_45262.4	141	109422	65	53323	0.3867	1.057098	9/3	165	0.64495
IL_06468.1	235	109328	110	53278	0.3879	1.041089	10/4	166	0.64495
IL_91379.1	7	109556	2	53386	0.3913	1.705524	12/2	167	0.64671
IL_57977.1	67	109496	30	53358	0.3954	1.088313	11/4	168	0.64959
IL_06421.1	96	109467	44	53344	0.4065	1.063213	7/4	169	0.66383
IL_71942.1	9	109554	3	53385	0.4095	1.461879	10/2	170	0.66487
IL_40892.1	95	109468	44	53344	0.4294	1.052129	8/3	171	0.69300
IL_52509.1	492	109071	236	53152	0.4383	1.015938	10/4	172	0.70340
IL_47732.1	17	109546	7	53381	0.4473	1.183409	6/2	173	0.70554
IL_82601.1	2	109561	0	53388	0.4521	Inf	8/4	174	0.70554
IL_94403.5	2	109561	0	53388	0.4521	Inf	11/3	175	0.70554
IL_94744.1	2	109561	0	53388	0.4521	Inf	17/8	176	0.70554
IL_59529.1	470	109093	226	53162	0.4525	1.013436	10/5	177	0.70554
IL_02835.1	4	109559	1	53387	0.4722	1.949152	13/4	178	0.73219
IL_28003.1	60	109503	28	53360	0.4752	1.044197	8/4	179	0.73270
IL_54966.1	272	109291	131	53257	0.4797	1.011794	9/5	180	0.73543
IL_35043.1	6	109557	2	53386	0.4823	1.461866	10/3	181	0.73543
IL_70401.1	10	109553	4	53384	0.4939	1.218198	11/3	182	0.74899
IL_09882.1	94	109469	45	53343	0.5012	1.017903	12/6	183	0.75184
IL_97191.1	14	109549	6	53382	0.5012	1.136999	13/7	184	0.75184
IL_06180.1	875	108688	426	52962	0.5073	1.000878	9/4	185	0.75678
IL_97057.3	659	108904	321	53067	0.5130	1.000371	11/5	186	0.76121
IL_87523.1	130	109433	63	53325	0.5198	1.005509	10/3	187	0.76725
IL_21077.1	154	109409	75	53313	0.5297	1.000552	8/2	188	0.77700
IL_83250.1	109	109454	53	53335	0.5321	1.002147	11/5	189	0.77700
IL_86336.1	272	109291	133	53255	0.5366	0.9965366	9/2	190	0.77941
IL_46301.1	249	109314	122	53266	0.5443	0.9945207	9/3	191	0.78653
IL_97833.1	27	109536	13	53375	0.5604	1.012054	14/8	192	0.80081
IL_95652.3	31	109532	15	53373	0.5606	1.007052	14/7	193	0.80081
IL_80652.1	15	109548	7	53381	0.5629	1.044179	13/6	194	0.80081
IL_52958.1	2521	107042	1237	52151	0.5878	0.9929122	9/2	195	0.83196
IL_03110.1	349	109214	173	53215	0.5934	0.9829487	13/5	196	0.83550
IL_82563.1	44	109519	22	53366	0.5963	0.9745314	10/3	197	0.83550
IL_54954.1	3	109560	1	53387	0.6027	1.461853	16/7	198	0.84017
IL_91044.1	309	109254	154	53234	0.6116	0.9776475	8/3	199	0.84822
IL_46034.3	79	109484	40	53348	0.6200	0.9623696	8/3	200	0.85558
IL_91078.1	12	109551	6	53382	0.6286	0.9745388	12/4	201	0.86310
IL_45794.1	39	109524	20	53368	0.6325	0.9501816	11/5	202	0.86418
IL_54470.1	325	109238	163	53225	0.6383	0.9714624	8/4	203	0.86789
IL_33842.1	23	109540	12	53376	0.6514	0.9339436	7/4	204	0.87410
IL_87394.1	23	109540	12	53376	0.6514	0.9339436	10/2	205	0.87410
IL_26868.1	382	109181	192	53196	0.6553	0.96935	10/5	206	0.87410
IL_78513.1	21	109542	11	53377	0.6556	0.9302528	8/2	207	0.87410
IL_12507.1	77	109486	40	53348	0.6686	0.9379743	11/4	208	0.87950
IL_05513.1	1	109562	0	53388	0.6724	Inf	12/3	209	0.87950
IL_27668.1	1	109562	0	53388	0.6724	Inf	11/3	210	0.87950
IL_42891.1	1	109562	0	53388	0.6724	Inf	14/3	211	0.87950

Continued on next page

Table D – continued from previous page

ModelID	A	B	C	D	P-value	Odds ratio	Nuc./Bp	Rank	Adj. p-value
IL_49493.4	102	109461	53	53335	0.6818	0.9377301	13/7	212	0.88138
IL_37053.1	231	109332	118	53270	0.6842	0.9538184	12/6	213	0.88138
IL_82188.2	215	109348	110	53278	0.6846	0.9523206	14/3	214	0.88138
IL_65137.1	88	109475	46	53342	0.6866	0.9321375	12/6	215	0.88138
IL_75415.1	4	109559	2	53386	0.6916	0.9745407	10/2	216	0.88371
IL_49527.1	720	108843	362	53026	0.6994	0.9690047	10/4	217	0.88957
IL_48918.3	69	109494	37	53351	0.7195	0.9086598	12/6	218	0.90711
IL_03282.1	104	109459	55	53333	0.7198	0.9213324	10/5	219	0.90711
IL_89794.1	25	109538	14	53374	0.7257	0.8701239	11/7	220	0.91042
IL_24293.1	66	109497	36	53352	0.7444	0.89329	8/3	221	0.92616
IL_27640.1	2	109561	1	53387	0.7483	0.9745412	15/4	222	0.92616
IL_86981.1	2	109561	1	53387	0.7483	0.9745412	15/8	223	0.92616
IL_82650.1	10	109553	6	53382	0.7535	0.8121377	13/5	224	0.92844
IL_53988.1	92	109471	50	53338	0.7631	0.8965155	12/5	225	0.93601
IL_16415.2	13	109550	8	53380	0.7775	0.7918313	14/7	226	0.94957
IL_59934.1	83	109480	46	53342	0.7878	0.8791404	12/5	227	0.95405
IL_72158.3	119	109444	65	53323	0.7946	0.8919876	12/6	228	0.95405
IL_66744.1	73	109490	41	53347	0.7973	0.8675189	13/3	229	0.95405
IL_31754.1	6	109557	4	53384	0.7984	0.7308838	13/7	230	0.95405
IL_76486.1	3	109560	2	53386	0.7985	0.7308912	15/8	231	0.95405
IL_31707.1	9	109554	6	53382	0.8105	0.7308765	11/4	232	0.96419
IL_44067.4	12	109551	8	53380	0.8243	0.7308691	14/6	233	0.97245
IL_92321.4	445	109118	233	53155	0.8245	0.9303635	12/6	234	0.97245
IL_96446.1	109	109454	61	53327	0.8289	0.8705953	10/3	235	0.97348
IL_85805.1	890	108673	458	52930	0.8370	0.9464665	8/2	236	0.97643
IL_92114.3	67	109496	39	53349	0.8385	0.8370388	9/4	237	0.97643
IL_25307.1	59	109504	35	53353	0.8492	0.8213382	9/3	238	0.98320
IL_81398.1	3058	106505	1538	51850	0.8514	0.9679943	10/4	239	0.98320
IL_60649.1	42	109521	27	53361	0.8941	0.7579321	11/5	240	1.00000
IL_62499.7	446	109117	242	53146	0.9173	0.8976353	7/4	241	1.00000
IL_46990.1	1687	107876	870	52518	0.9175	0.9440167	10/2	242	1.00000
IL_98924.1	391	109172	215	53173	0.9285	0.8857705	9/3	243	1.00000
IL_52940.1	226	109337	129	53259	0.9314	0.8533953	14/6	244	1.00000
IL_47687.1	230	109333	133	53255	0.9475	0.8423491	13/6	245	1.00000
IL_90735.1	18	109545	15	53373	0.9561	0.5846575	16/3	246	1.00000
IL_05221.1	2	109561	3	53385	0.9575	0.3248442	15/11	247	1.00000
IL_94973.1	12	109551	11	53377	0.9577	0.5315074	14/6	248	1.00000
IL_27243.1	1	109562	2	53386	0.9648	0.2436375	14/7	249	1.00000
IL_25181.1	1001	108562	540	52848	0.9735	0.9023875	13/5	250	1.00000
IL_30840.2	2233	107330	1171	52217	0.9807	0.9277342	11/5	251	1.00000
IL_53323.1	969	108594	528	52860	0.9819	0.8933337	10/3	252	1.00000
IL_68827.1	24	109539	23	53365	0.9929	0.5083444	11/4	253	1.00000
IL_49976.1	1024	108539	589	52799	0.9994	0.8457285	8/5	254	1.00000
IL_02359.3	0	109563	0	53388	1.0000	0	16/9	255	1.00000
IL_16330.1	0	109563	0	53388	1.0000	0	25/5	256	1.00000
IL_17682.1	0	109563	1	53387	1.0000	0	17/8	257	1.00000
IL_21421.1	0	109563	0	53388	1.0000	0	20/7	258	1.00000
IL_21495.1	0	109563	0	53388	1.0000	0	14/5	259	1.00000
IL_23414.1	0	109563	0	53388	1.0000	0	21/5	260	1.00000
IL_25230.3	0	109563	0	53388	1.0000	0	18/8	261	1.00000
IL_33964.1	0	109563	0	53388	1.0000	0	20/6	262	1.00000
IL_39324.1	0	109563	0	53388	1.0000	0	18/10	263	1.00000
IL_40527.1	0	109563	0	53388	1.0000	0	15/5	264	1.00000
IL_52610.1	0	109563	0	53388	1.0000	0	36/9	265	1.00000
IL_57285.1	0	109563	0	53388	1.0000	0	13/4	266	1.00000
IL_76095.3	0	109563	0	53388	1.0000	0	18/8	267	1.00000
IL_76263.1	0	109563	0	53388	1.0000	0	17/3	268	1.00000
IL_77076.1	0	109563	0	53388	1.0000	0	18/8	269	1.00000
IL_77296.1	0	109563	0	53388	1.0000	0	14/2	270	1.00000

Continued on next page

Table D – continued from previous page

ModelID	A	B	C	D	P-value	Odds ratio	Nuc./Bp	Rank	Adj. p-value
IL_88367.1	0	109563	0	53388	1.0000	0	15/7	271	1.00000
IL_89028.6	0	109563	0	53388	1.0000	0	13/5	272	1.00000
IL_90057.1	0	109563	0	53388	1.0000	0	23/17	273	1.00000
IL_91904.1	0	109563	0	53388	1.0000	0	13/4	274	1.00000
IL_97842.1	0	109563	0	53388	1.0000	0	16/5	275	1.00000
IL_98655.1	0	109563	0	53388	1.0000	0	13/5	276	1.00000

Table E: Results of Fisher's exact test according to FET scheme (see Supplementary Material S6) for JAR3D IL module predictions on SISSIz and original data of order R. Red highlighted rows have a significant p-value after multiple testing correction using the Benjamini-Hochberg procedure at a 5% false discovery rate (adjusted p-value ≤0.05) and average 3D sequence length ≥9. "Nuc./Bp" denotes the total strand length and the number of base pairs.

ModelID	A	B	C	D	P-value	Odds ratio	Nuc./Bp	Rank	Adj. p-value
IL_47875.1	546	110251	149	53239	6.283474e-11	1.769507	4/2	1	1.734238824e-08
IL_96206.3	295	110502	63	53325	1.524187e-10	2.259645	5/4	2	2.10337806e-08
IL_73000.2	254	110543	54	53334	2.369698e-09	2.269399	6/4	3	1.76879223e-07
IL_92109.3	524	110273	150	53238	2.563467e-09	1.686519	4/2	4	1.76879223e-07
IL_28947.2	329	110468	83	53305	1.563343e-08	1.912703	4/2	5	8.62965336e-07
IL_97217.11	345	110452	92	53296	7.166728e-08	1.809467	4/2	6	3.29669488e-06
IL_58586.2	296	110501	77	53311	2.405906e-07	1.854599	6/2	7	9.48614365714286e-06
IL_56465.4	479	110318	146	53242	3.019554e-07	1.583396	5/2	8	1.04174613e-05
IL_94430.5	353	110444	102	53286	1.310732e-06	1.669722	5/3	9	4.01957813333333e-05
IL_39199.4	269	110528	72	53316	2.073315e-06	1.802202	5/4	10	5.7223494e-05
IL_28644.1	156	110641	33	53355	2.423588e-06	2.279647	5/2	11	6.08100261818182e-05
IL_23448.1	89	110708	13	53375	3.739168e-06	3.300605	6/2	12	8.6000864e-05
IL_25300.3	289	110508	81	53307	4.178941e-06	1.721084	4/2	13	8.8722132e-05
IL_58291.4	125	110672	25	53363	9.152472e-06	2.410851	6/2	14	0.00018
IL_73276.5	180	110617	44	53344	1.249924e-05	1.972794	7/5	15	0.00022
IL_67887.1	211	110586	55	53333	1.256306e-05	1.85018	5/3	16	0.00022
IL_87904.5	1161	109636	444	52944	1.337971e-05	1.262695	10/5	17	0.00022
IL_70173.1	178	110619	44	53344	1.78963e-05	1.950839	6/2	18	0.00027
IL_44540.4	207	110590	55	53333	2.436051e-05	1.81504	5/4	19	0.00034
IL_23262.4	463	110334	154	53234	2.46736e-05	1.450571	10/3	20	0.00034
IL_92027.3	154	110643	37	53351	3.658246e-05	2.006947	6/4	21	0.00048
IL_69799.1	118	110679	25	53363	4.048887e-05	2.2757	5/2	22	0.00051
IL_46648.6	222	110575	62	53326	4.643344e-05	1.7268	5/3	23	0.00056
IL_79083.3	654	110143	236	53152	5.695096e-05	1.337336	11/4	24	0.00065
IL_74876.2	876	109921	332	53056	8.219327e-05	1.273516	10/4	25	0.00091
IL_24982.5	1362	109435	545	52843	0.0001	1.206711	10/5	26	0.00112
IL_43316.1	840	109957	320	53068	0.0002	1.266848	10/4	27	0.00154
IL_01080.1	208	110589	60	53328	0.0002	1.671684	7/2	28	0.00168
IL_02809.3	231	110566	69	53319	0.0002	1.614441	8/4	29	0.00178
IL_40845.1	172	110625	48	53340	0.0003	1.727768	7/4	30	0.00284
IL_86357.3	79	110718	16	53372	0.0005	2.380128	6/3	31	0.00385
IL_63133.1	53	110744	8	53380	0.0005	3.193292	6/3	32	0.00385
IL_83856.1	547	110250	201	53187	0.0005	1.312904	12/6	33	0.00385
IL_21333.2	243	110554	77	53311	0.0006	1.521797	8/4	34	0.00472
IL_63952.1	517	110280	191	53197	0.0008	1.30576	13/4	35	0.00625
IL_90459.3	478	110319	176	53212	0.0011	1.310056	6/3	36	0.00823
IL_80494.2	491	110306	182	53206	0.0012	1.301331	12/5	37	0.00884
IL_80348.3	186	110611	57	53331	0.0012	1.573325	5/2	38	0.00901

Continued on next page

Table E – continued from previous page

ModelID	A	B	C	D	P-value	Odds ratio	Nuc./Bp	Rank	Adj. p-value
IL_87065.1	136	110661	38	53350	0.0013	1.725416	5/3	39	0.00901
IL_34628.2	52	110745	9	53379	0.0014	2.784867	12/6	40	0.00954
IL_47174.11	277	110520	94	53294	0.0016	1.420992	6/3	41	0.01049
IL_39585.1	1037	109760	425	52963	0.0024	1.177369	10/6	42	0.01568
IL_26971.1	868	109929	351	53037	0.0027	1.193088	10/3	43	0.01747
IL_39980.1	100	110697	27	53361	0.0035	1.785348	6/3	44	0.02194
IL_12147.1	200	110597	66	53322	0.0038	1.460993	6/3	45	0.02267
IL_88119.1	185	110612	60	53328	0.0038	1.486526	6/3	46	0.02267
IL_09587.1	2726	108071	1199	52189	0.0039	1.097931	11/4	47	0.02291
IL_42251.1	201	110596	67	53321	0.0045	1.44637	6/3	48	0.02575
IL_98421.4	103	110694	29	53359	0.0051	1.71207	7/2	49	0.02869
IL_17212.2	212	110585	72	53316	0.0052	1.419605	6/3	50	0.02869
IL_68767.1	100	110697	28	53360	0.0054	1.721554	6/2	51	0.02930
IL_43877.1	86	110711	23	53365	0.0059	1.802332	6/2	52	0.03089
IL_69536.1	1122	109675	471	52917	0.0059	1.149359	8/3	53	0.03089
IL_63253.1	91	110706	25	53363	0.0063	1.754563	6/2	54	0.03168
IL_70237.3	936	109861	388	53000	0.0063	1.163782	10/4	55	0.03168
IL_91089.1	39	110758	7	53381	0.0068	2.685193	8/2	56	0.03329
IL_82444.1	175	110622	58	53330	0.0069	1.454585	6/3	57	0.03338
IL_37406.1	82	110715	22	53366	0.0073	1.796585	7/2	58	0.03473
IL_80093.1	653	110144	264	53124	0.0082	1.192978	10/5	59	0.03818
IL_46721.1	97	110700	28	53360	0.0086	1.669862	10/3	60	0.03972
IL_21639.1	44	110753	9	53379	0.0089	2.356259	9/3	61	0.04041
IL_55649.1	397	110400	153	53235	0.0097	1.251166	8/3	62	0.04336
IL_86059.1	108	110689	33	53355	0.0116	1.577536	4/2	63	0.04982
IL_55938.4	297	110500	111	53277	0.0117	1.290013	7/4	64	0.04982
IL_31006.1	185	110612	64	53324	0.0117	1.393536	11/5	65	0.04982
IL_39355.1	350	110447	134	53254	0.0123	1.259356	8/3	66	0.05084
IL_37197.1	655	110142	268	53120	0.0123	1.178707	11/4	67	0.05084
IL_37104.3	193	110604	68	53320	0.0140	1.368286	6/3	68	0.05606
IL_41791.1	70	110727	19	53369	0.0140	1.775736	11/8	69	0.05606
IL_18675.1	91	110706	27	53361	0.0144	1.624535	7/2	70	0.05657
IL_13777.1	119	110678	38	53350	0.0146	1.509508	5/2	71	0.05657
IL_16166.4	136	110661	45	53343	0.0154	1.456828	5/2	72	0.05919
IL_37325.1	50	110747	12	53376	0.0159	2.008173	7/4	73	0.06005
IL_69106.1	193	110604	69	53319	0.0178	1.348438	7/3	74	0.06656
IL_15840.2	706	110091	294	53094	0.0183	1.158102	11/6	75	0.06728
IL_06808.1	84	110713	25	53363	0.0188	1.619495	8/4	76	0.06728
IL_06471.1	488	110309	197	53191	0.0188	1.194466	10/7	77	0.06728
IL_13959.4	160	110637	56	53332	0.0214	1.377297	8/4	78	0.07570
IL_05723.1	485	110312	197	53191	0.0226	1.187093	10/5	79	0.07894
IL_98566.1	82	110715	25	53363	0.0251	1.580907	8/2	80	0.08533
IL_41766.6	345	110452	136	53252	0.0251	1.223019	8/4	81	0.08533
IL_95150.6	69	110728	20	53368	0.0254	1.662805	7/3	82	0.08533
IL_97509.1	733	110064	311	53077	0.0311	1.136584	10/5	83	0.10357
IL_05684.1	231	110566	88	53300	0.0329	1.265381	8/3	84	0.10758
IL_85510.1	683	110114	289	53099	0.0333	1.139629	6/2	85	0.10758
IL_15205.1	117	110680	40	53348	0.0340	1.40987	12/5	86	0.10758
IL_57364.1	26	110771	5	53383	0.0341	2.50598	8/4	87	0.10758
IL_80714.1	469	110328	193	53195	0.0343	1.171643	12/3	88	0.10758
IL_31039.1	59	110738	17	53371	0.0356	1.672671	7/3	89	0.11032
IL_06177.1	40	110757	10	53378	0.0371	1.927744	6/2	90	0.11245
IL_31066.3	40	110757	10	53378	0.0371	1.927744	5/2	91	0.11245
IL_31663.1	94	110703	31	53357	0.0380	1.461495	7/4	92	0.11407
IL_17603.1	352	110445	142	53246	0.0395	1.195056	11/5	93	0.11717
IL_90133.3	146	110651	53	53335	0.0431	1.327846	7/3	94	0.12646
IL_93424.4	626	110171	266	53122	0.0449	1.134741	9/4	95	0.13054
IL_54450.1	41	110756	11	53377	0.0509	1.796291	8/3	96	0.14594
IL_56513.1	94	110703	32	53356	0.0513	1.415811	5/2	97	0.14594

Continued on next page

Table E – continued from previous page

ModelID	A	B	C	D	P-value	Odds ratio	Nuc./Bp	Rank	Adj. p-value
IL_61730.1	24	110773	5	53383	0.0544	2.313174	11/3	98	0.15308
IL_12211.1	129	110668	47	53341	0.0568	1.322956	12/6	99	0.15841
IL_06847.1	7	110790	0	53388	0.0637	Inf	11/4	100	0.17589
IL_25271.2	26	110771	6	53382	0.0657	2.088281	14/7	101	0.17795
IL_20775.1	65	110732	21	53367	0.0658	1.491738	7/3	102	0.17795
IL_97296.1	208	110589	82	53306	0.0681	1.222656	7/3	103	0.18251
IL_87548.1	414	110383	175	53213	0.0782	1.140446	11/6	104	0.20514
IL_46306.1	1168	109629	522	52866	0.0786	1.079003	9/3	105	0.20514
IL_23639.1	1183	109614	529	52859	0.0788	1.078403	9/4	106	0.20514
IL_79955.2	360	110437	151	53237	0.0819	1.149267	12/6	107	0.20980
IL_50911.1	33	110764	9	53379	0.0821	1.767021	7/3	108	0.20980
IL_34363.2	19	110778	4	53384	0.0880	2.289018	13/9	109	0.22186
IL_52958.1	2689	108108	1237	52151	0.0884	1.048638	9/2	110	0.22186
IL_46435.1	6	110791	0	53388	0.0944	Inf	13/2	111	0.23299
IL_17066.1	67	110730	23	53365	0.0954	1.403923	6/3	112	0.23299
IL_30381.1	67	110730	23	53365	0.0954	1.403923	8/4	113	0.23299
IL_00998.1	100	110697	37	53351	0.0978	1.302631	8/4	114	0.23686
IL_13069.3	81	110716	29	53359	0.0994	1.346159	4/2	115	0.23853
IL_65553.8	102	110695	38	53350	0.1011	1.293618	12/7	116	0.24065
IL_22909.1	101	110696	38	53350	0.1114	1.280928	9/3	117	0.25993
IL_47444.3	735	110062	326	53062	0.1115	1.086963	12/6	118	0.25993
IL_41139.1	56	110741	19	53369	0.1126	1.420424	7/2	119	0.25993
IL_06211.3	133	110664	52	53336	0.1137	1.232685	7/2	120	0.25993
IL_80505.1	9	110788	1	53387	0.1140	4.337415	11/2	121	0.25993
IL_58454.1	610	110187	269	53119	0.1189	1.093191	9/3	122	0.26909
IL_93568.2	202	110595	83	53305	0.1222	1.173007	12/5	123	0.27431
IL_08926.3	104	110693	40	53348	0.1295	1.253023	5/3	124	0.28643
IL_47758.2	120	110677	47	53341	0.1298	1.230487	8/4	125	0.28643
IL_50521.1	50	110747	17	53371	0.1308	1.417418	14/7	126	0.28643
IL_53635.3	101	110696	39	53349	0.1380	1.248071	11/5	127	0.29651
IL_28572.2	5	110792	0	53388	0.1399	Inf	15/5	128	0.29651
IL_64589.1	59	110738	21	53367	0.1400	1.354004	7/2	129	0.29651
IL_87507.1	211	110586	88	53300	0.1402	1.155641	9/4	130	0.29651
IL_31555.5	612	110185	272	53116	0.1407	1.084637	9/4	131	0.29651
IL_57785.5	49	110748	17	53371	0.1485	1.389068	8/4	132	0.31052
IL_43124.2	174	110623	72	53316	0.1537	1.164727	7/4	133	0.31886
IL_59529.1	509	110288	226	53162	0.1620	1.085629	10/5	134	0.33362
IL_77263.2	274	110523	118	53270	0.1666	1.119171	10/5	135	0.34066
IL_24546.4	130	110667	53	53335	0.1717	1.182104	10/3	136	0.34531
IL_09333.1	317	110480	138	53250	0.1719	1.10717	12/6	137	0.34531
IL_09491.1	31	110766	10	53378	0.1731	1.493883	7/2	138	0.34531
IL_11302.1	850	109947	386	53002	0.1739	1.061549	10/3	139	0.34531
IL_11751.1	33	110764	11	53377	0.1841	1.445695	7/2	140	0.36287
IL_41153.1	28	110769	9	53379	0.1884	1.499225	7/3	141	0.36881
IL_09530.1	667	110130	302	53086	0.1935	1.064615	8/3	142	0.37618
IL_22732.1	174	110623	74	53314	0.2031	1.13321	9/5	143	0.39191
IL_77014.1	4	110793	0	53388	0.2074	Inf	11/5	144	0.39617
IL_25380.1	167	110630	71	53317	0.2081	1.133569	8/4	145	0.39617
IL_06180.1	927	109870	426	52962	0.2169	1.048951	9/4	146	0.40998
IL_71685.1	75	110722	30	53358	0.2255	1.204753	14/7	147	0.42339
IL_97057.3	700	110097	321	53067	0.2415	1.051094	11/5	148	0.45030
IL_70299.1	83	110714	34	53354	0.2439	1.176409	10/4	149	0.45185
IL_91379.1	9	110788	2	53386	0.2520	2.168429	12/2	150	0.46377
IL_85647.3	14	110783	4	53384	0.2539	1.686571	15/8	151	0.46412
IL_06306.1	35	110762	13	53375	0.2616	1.297342	13/2	152	0.47372
IL_75328.1	21	110776	7	53381	0.2637	1.445647	14/4	153	0.47372
IL_06421.1	104	110693	44	53344	0.2643	1.139051	7/4	154	0.47372
IL_21254.1	37	110760	14	53374	0.2705	1.273521	12/7	155	0.48158
IL_99397.1	16	110781	5	53383	0.2744	1.542008	9/4	156	0.48414

Continued on next page

Table E – continued from previous page

ModelID	A	B	C	D	P-value	Odds ratio	Nuc./Bp	Rank	Adj. p-value
IL_06468.1	246	110551	110	53278	0.2771	1.077772	10/4	157	0.48414
IL_39526.4	6	110791	1	53387	0.2787	2.89121	9/2	158	0.48414
IL_30067.1	278	110519	125	53263	0.2789	1.071822	10/5	159	0.48414
IL_91044.1	339	110458	154	53234	0.2893	1.06089	8/3	160	0.49691
IL_88865.1	25	110772	9	53379	0.2899	1.3386	8/3	161	0.49691
IL_11778.1	34	110763	13	53375	0.2940	1.260276	7/2	162	0.50089
IL_43946.1	13	110784	4	53384	0.3060	1.566088	12/6	163	0.51539
IL_78744.1	20	110777	7	53381	0.3062	1.376821	12/6	164	0.51539
IL_45262.4	147	110650	65	53323	0.3094	1.089848	9/3	165	0.51754
IL_83920.1	60	110737	25	53363	0.3139	1.156524	7/3	166	0.51976
IL_07300.2	123	110674	54	53334	0.3145	1.097662	9/4	167	0.51976
IL_38807.3	61	110736	26	53362	0.3452	1.130569	11/6	168	0.56719
IL_09882.1	101	110696	45	53343	0.3670	1.081567	12/6	169	0.59662
IL_98591.3	5	110792	1	53387	0.3675	2.409323	13/8	170	0.59662
IL_37347.1	14	110783	5	53383	0.3804	1.349272	15/7	171	0.60938
IL_31224.1	276	110521	128	53260	0.3823	1.03908	7/2	172	0.60938
IL_52509.1	503	110294	236	53152	0.3840	1.027151	10/4	173	0.60938
IL_81398.1	3222	107575	1538	51850	0.3856	1.009733	10/4	174	0.60938
IL_54966.1	282	110515	131	53257	0.3865	1.03735	9/5	175	0.60938
IL_57977.1	68	110729	30	53358	0.3886	1.092257	11/4	176	0.60938
IL_47732.1	18	110779	7	53381	0.4036	1.239062	6/2	177	0.62935
IL_52173.1	171	110626	79	53309	0.4072	1.043062	9/2	178	0.63143
IL_28003.1	62	110735	28	53360	0.4367	1.066999	8/4	179	0.67339
IL_46301.1	259	110538	122	53266	0.4420	1.023024	9/3	180	0.67773
IL_25082.1	2	110795	0	53388	0.4554	Inf	17/6	181	0.68309
IL_82601.1	2	110795	0	53388	0.4554	Inf	8/4	182	0.68309
IL_94403.5	2	110795	0	53388	0.4554	Inf	11/3	183	0.68309
IL_94744.1	2	110795	0	53388	0.4554	Inf	17/8	184	0.68309
IL_86336.1	280	110517	133	53255	0.4691	1.014474	9/2	185	0.69802
IL_21077.1	159	110638	75	53313	0.4704	1.021577	8/2	186	0.69802
IL_02835.1	4	110793	1	53387	0.4771	1.927443	13/4	187	0.70052
IL_40892.1	94	110703	44	53344	0.4772	1.02947	8/3	188	0.70052
IL_03110.1	362	110435	173	53215	0.4850	1.008302	13/5	189	0.70820
IL_35043.1	6	110791	2	53386	0.4884	1.445586	10/3	190	0.70947
IL_83250.1	112	110685	53	53335	0.4937	1.018288	11/5	191	0.71307
IL_71942.1	8	110789	3	53385	0.4960	1.284918	10/2	192	0.71307
IL_70401.1	10	110787	4	53384	0.5019	1.204633	11/3	193	0.71420
IL_26868.1	400	110397	192	53196	0.5020	1.003877	10/5	194	0.71420
IL_97191.1	14	110783	6	53382	0.5107	1.124336	13/7	195	0.72287
IL_97833.1	28	110769	13	53375	0.5301	1.037831	14/8	196	0.74645
IL_45794.1	42	110755	20	53368	0.5429	1.011903	11/5	197	0.76058
IL_49493.4	109	110688	53	53335	0.5586	0.9909743	13/7	198	0.77872
IL_49527.1	745	110052	362	53026	0.5663	0.9916048	10/4	199	0.77918
IL_46034.3	82	110715	40	53348	0.5680	0.987792	8/3	200	0.77918
IL_80652.1	15	110782	7	53381	0.5727	1.032519	13/6	201	0.77918
IL_33842.1	25	110772	12	53376	0.5731	1.003864	7/4	202	0.77918
IL_87394.1	25	110772	12	53376	0.5731	1.003864	10/2	203	0.77918
IL_87523.1	128	110669	63	53325	0.5885	0.9789664	10/3	204	0.79622
IL_85805.1	939	109858	458	52930	0.5972	0.987798	8/2	205	0.80406
IL_54954.1	3	110794	1	53387	0.6071	1.445574	16/7	206	0.81334
IL_82563.1	44	110753	22	53366	0.6129	0.9637125	10/3	207	0.81334
IL_95652.3	30	110767	15	53373	0.6152	0.963717	14/7	208	0.81334
IL_24293.1	72	110725	36	53352	0.6159	0.9637033	8/3	209	0.81334
IL_37053.1	238	110559	118	53270	0.6241	0.9717888	12/6	210	0.82020
IL_54470.1	329	110468	163	53225	0.6345	0.9724704	8/4	211	0.82867
IL_91078.1	12	110785	6	53382	0.6372	0.963723	12/4	212	0.82867
IL_48918.3	73	110724	37	53351	0.6416	0.9506537	12/6	213	0.82867
IL_65137.1	91	110706	46	53342	0.6425	0.9531956	12/6	214	0.82867
IL_92321.4	470	110327	233	53155	0.6553	0.9718353	12/6	215	0.84124

Continued on next page

Table E – continued from previous page

ModelID	A	B	C	D	P-value	Odds ratio	Nuc./Bp	Rank	Adj. p-value
IL_53988.1	98	110699	50	53338	0.6646	0.9443862	12/5	216	0.84661
IL_12507.1	78	110719	40	53348	0.6657	0.9395745	11/4	217	0.84661
IL_05513.1	1	110796	0	53388	0.6748	Inf	12/3	218	0.84661
IL_27668.1	1	110796	0	53388	0.6748	Inf	11/3	219	0.84661
IL_42891.1	1	110796	0	53388	0.6748	Inf	14/3	220	0.84661
IL_16415.2	15	110782	8	53380	0.6823	0.9034678	14/7	221	0.85131
IL_03282.1	107	110690	55	53333	0.6847	0.9373664	10/5	222	0.85131
IL_82188.2	217	110580	110	53278	0.6907	0.9504708	14/3	223	0.85485
IL_75415.1	4	110793	2	53386	0.6964	0.9637256	10/2	224	0.85808
IL_72158.3	126	110671	65	53323	0.7022	0.9339824	12/6	225	0.86135
IL_78513.1	20	110777	11	53377	0.7120	0.8760839	8/2	226	0.86955
IL_92114.3	73	110724	39	53349	0.7352	0.9018719	9/4	227	0.89181
IL_89794.1	25	110772	14	53374	0.7367	0.8604327	11/7	228	0.89181
IL_46990.1	1759	109038	870	52518	0.7445	0.9737936	10/2	229	0.89731
IL_27640.1	2	110795	1	53387	0.7516	0.9637262	15/4	230	0.89797
IL_86981.1	2	110795	1	53387	0.7516	0.9637262	15/8	231	0.89797
IL_82650.1	10	110787	6	53382	0.7603	0.8030946	13/5	232	0.90453
IL_59934.1	84	110713	46	53342	0.7869	0.8798237	12/5	233	0.93218
IL_76486.1	3	110794	2	53386	0.8021	0.7227533	15/8	234	0.94344
IL_31754.1	6	110791	4	53384	0.8033	0.7227458	13/7	235	0.94344
IL_96446.1	111	110686	61	53327	0.8183	0.8767007	10/3	236	0.95701
IL_44067.4	12	110785	8	53380	0.8305	0.7227308	14/6	237	0.96322
IL_66744.1	72	110725	41	53347	0.8306	0.8460958	13/3	238	0.96322
IL_98924.1	412	110385	215	53173	0.8395	0.9230835	9/3	239	0.96950
IL_60649.1	45	110752	27	53361	0.8481	0.8030324	11/5	240	0.97529
IL_62499.7	463	110334	242	53146	0.8571	0.9215694	7/4	241	0.98159
IL_25307.1	59	110738	35	53353	0.8610	0.8121884	9/3	242	0.98198
IL_31707.1	8	110789	6	53382	0.8658	0.6424372	11/4	243	0.98339
IL_30840.2	2333	108464	1171	52217	0.8789	0.959153	11/5	244	0.99420
IL_53323.1	1024	109773	528	52860	0.9025	0.9338958	10/3	245	1.00000
IL_05221.1	3	110794	3	53385	0.9077	0.4818301	15/11	246	1.00000
IL_52940.1	229	110568	129	53259	0.9294	0.8550962	14/6	247	1.00000
IL_47687.1	236	110561	133	53255	0.9327	0.8547212	13/6	248	1.00000
IL_94973.1	13	110784	11	53377	0.9432	0.5694015	14/6	249	1.00000
IL_25181.1	1027	109770	540	52848	0.9528	0.9156363	13/5	250	1.00000
IL_90735.1	18	110779	15	53373	0.9590	0.5781455	16/3	251	1.00000
IL_27243.1	1	110796	2	53386	0.9656	0.240924	14/7	252	1.00000
IL_68827.1	26	110771	23	53365	0.9879	0.5445731	11/4	253	1.00000
IL_49976.1	1049	109748	589	52799	0.9986	0.8568302	8/5	254	1.00000
IL_02359.3	0	110797	0	53388	1.0000	0	16/9	255	1.00000
IL_16330.1	0	110797	0	53388	1.0000	0	25/5	256	1.00000
IL_17682.1	0	110797	1	53387	1.0000	0	17/8	257	1.00000
IL_21421.1	0	110797	0	53388	1.0000	0	20/7	258	1.00000
IL_21495.1	0	110797	0	53388	1.0000	0	14/5	259	1.00000
IL_23414.1	0	110797	0	53388	1.0000	0	21/5	260	1.00000
IL_25230.3	0	110797	0	53388	1.0000	0	18/8	261	1.00000
IL_33964.1	0	110797	0	53388	1.0000	0	20/6	262	1.00000
IL_39324.1	0	110797	0	53388	1.0000	0	18/10	263	1.00000
IL_40527.1	0	110797	0	53388	1.0000	0	15/5	264	1.00000
IL_52610.1	0	110797	0	53388	1.0000	0	36/9	265	1.00000
IL_57285.1	0	110797	0	53388	1.0000	0	13/4	266	1.00000
IL_76095.3	0	110797	0	53388	1.0000	0	18/8	267	1.00000
IL_76263.1	0	110797	0	53388	1.0000	0	17/3	268	1.00000
IL_77076.1	0	110797	0	53388	1.0000	0	18/8	269	1.00000
IL_77296.1	0	110797	0	53388	1.0000	0	14/2	270	1.00000
IL_88367.1	0	110797	0	53388	1.0000	0	15/7	271	1.00000
IL_89028.6	0	110797	0	53388	1.0000	0	13/5	272	1.00000
IL_90057.1	0	110797	0	53388	1.0000	0	23/17	273	1.00000
IL_91904.1	0	110797	0	53388	1.0000	0	13/4	274	1.00000

Continued on next page

Table E – continued from previous page

ModelID	A	B	C	D	P-value	Odds ratio	Nuc./Bp	Rank	Adj. p-value
IL_97842.1	0	110797	0	53388	1.0000	0	16/5	275	1.00000
IL_98655.1	0	110797	0	53388	1.0000	0	13/5	276	1.00000

Table F: Results of Fisher's exact test according to FET scheme (see Supplementary Material S6) for JAR3D IL module predictions of all JAR3D IL models together with mean interior edit distance ≤ 4, passed cutoff ≥ 50, and average 3D sequence length ≥ 9.

Order	Model	A	B	C	D	P-value	Odds ratio
H	all	20309	88776	9324	43117	2.311515e-05	1.057894
L	all	19944	86698	9324	43117	4.032838e-06	1.063783
R	all	20488	87348	9324	43117	1.861548e-09	1.084674

Table G: Results of Fisher's exact test according to FET scheme (see Supplementary Material S6) for JAR3D HL module predictions for SISSIz and original data of order L. Brown highlighted rows have a significant p-value after multiple testing correction using the Benjamini-Hochberg procedure at a 5% false discovery rate (adjusted p-value). "Nuc./Bp" denotes the total strand length and the number of base pairs.

ModelID	A	B	C	D	P-value	Odds ratio	Nuc./Bp	Rank	Adj. p-value
HL_75759.4	5604	103959	2287	51101	7.009408e-14	1.204449	3/1	1	1.773380224e-11
HL_35865.1	5818	103745	2436	50952	4.03886e-11	1.17296	5/1	2	5.1091579e-09
HL_87554.1	1844	107719	705	52683	1.124556e-08	1.279282	5/1	3	8.607559675e-07
HL_06643.3	5252	104311	2233	51155	1.360879e-08	1.153423	5/2	4	8.607559675e-07
HL_90542.1	999	108564	358	53030	1.780895e-07	1.363093	7/2	5	9.0113287e-06
HL_74559.1	2744	106819	1138	52250	1.601183e-06	1.179432	4/1	6	6.75165498333e-05
HL_97499.1	2020	107543	823	52565	5.523972e-06	1.199659	4/1	7	0.00020
HL_42677.2	4451	105112	1935	51453	8.935428e-06	1.125983	3/1	8	0.00028
HL_30008.1	1970	107593	807	52581	1.257518e-05	1.192971	5/1	9	0.00035
HL_10378.1	1670	107893	674	52714	1.414211e-05	1.210542	4/1	10	0.00036
HL_05361.1	550	109013	192	53196	2.633234e-05	1.397866	6/2	11	0.00061
HL_63941.1	4344	105219	1903	51485	3.761264e-05	1.116953	4/1	12	0.00076
HL_74505.1	4075	105488	1779	51609	3.911024e-05	1.120653	4/1	13	0.00076
HL_59604.1	2217	107346	940	52448	0.0001	1.152329	3/1	14	0.00266
HL_65071.1	933	108630	366	53022	0.0002	1.244212	4/1	15	0.00326
HL_75850.1	3866	105697	1705	51683	0.0002	1.108716	5/1	16	0.00373
HL_99207.1	1816	107747	764	52624	0.0003	1.160904	7/3	17	0.00428
HL_34027.2	7001	102562	3180	50208	0.0003	1.07776	6/1	18	0.00483
HL_59225.1	1953	107610	838	52550	0.0009	1.138085	5/1	19	0.01250
HL_57514.2	9121	100442	4207	49181	0.0011	1.061583	4/1	20	0.01337
HL_17790.1	2113	107450	916	52472	0.0014	1.126477	3/1	21	0.01715
HL_35619.2	1451	108112	615	52773	0.0018	1.151664	5/2	22	0.01914
HL_86115.1	3996	105567	1795	51593	0.0018	1.087993	4/2	23	0.01914
HL_17723.4	1735	107828	745	52643	0.0018	1.13697	7/1	24	0.01914
HL_03785.1	3736	105827	1675	51713	0.0020	1.089918	5/1	25	0.02021
HL_26579.1	656	108907	262	53126	0.0032	1.221361	5/2	26	0.03118
HL_75579.2	15795	93768	7437	45951	0.0043	1.040791	3/1	27	0.03986

Continued on next page

Table G – continued from previous page

ModelID	A	B	C	D	P-value	Odds ratio	Nuc./Bp	Rank	Adj. p-value
HL_68435.1	831	108732	343	53045	0.0048	1.181919	7/2	28	0.04351
HL_45018.3	862	108701	358	53030	0.0055	1.174645	10/4	29	0.04789
HL_73465.1	28	109535	4	53384	0.0083	3.411463	11/2	30	0.07018
HL_01418.1	1536	108027	671	52717	0.0089	1.117081	5/1	31	0.07304
HL_00721.1	1277	108286	552	52836	0.0093	1.128773	2/1	32	0.07321
HL_42077.2	14710	94853	6949	46439	0.0112	1.03639	5/2	33	0.08590
HL_00090.1	744	108819	311	53077	0.0118	1.166834	11/1	34	0.08776
HL_18587.1	824	108739	348	53040	0.0128	1.154946	8/2	35	0.09277
HL_24707.1	1691	107872	748	52640	0.0136	1.103183	4/2	36	0.09368
HL_98523.2	4894	104669	2257	51131	0.0137	1.059254	6/1	37	0.09368
HL_46489.2	940	108623	403	52985	0.0160	1.13776	9/2	38	0.10667
HL_99584.1	1284	108279	563	52825	0.0185	1.112628	4/1	39	0.12003
HL_72273.1	736	108827	312	53076	0.0202	1.150484	6/2	40	0.12762
HL_48116.2	8582	100981	4028	49360	0.0209	1.041443	6/1	41	0.12866
HL_33277.1	1573	107990	701	52687	0.0246	1.094786	7/1	42	0.14837
HL_98833.3	738	108825	317	53071	0.0313	1.135331	8/2	43	0.18404
HL_19905.3	7800	101763	3670	49718	0.0354	1.038372	4/1	44	0.20364
HL_48039.2	18163	91400	8664	44724	0.0376	1.025803	4/1	45	0.21165
HL_50312.1	4273	105290	1991	51397	0.0473	1.047643	6/1	46	0.26022
HL_36842.1	1981	107582	904	52484	0.0512	1.069062	8/2	47	0.26135
HL_78420.1	546	109017	234	53154	0.0529	1.137667	9/1	48	0.26135
HL_66877.1	170	109393	65	53323	0.0534	1.274809	11/4	49	0.26135
HL_24711.1	713	108850	311	53077	0.0538	1.117905	7/2	50	0.26135
HL_73972.1	950	108613	421	52967	0.0542	1.100433	6/3	51	0.26135
HL_41827.1	1121	108442	501	52887	0.0553	1.091234	7/2	52	0.26135
HL_21675.3	7352	102211	3470	49918	0.0554	1.034752	5/2	53	0.26135
HL_34108.2	9524	100039	4515	48873	0.0566	1.030533	4/1	54	0.26135
HL_95716.1	5768	103795	2711	50677	0.0568	1.038797	5/1	55	0.26135
HL_78361.1	639	108924	279	53109	0.0662	1.116707	8/2	56	0.29892
HL_78507.1	2103	107460	968	52420	0.0716	1.059775	5/1	57	0.31766
HL_27397.1	370	109193	157	53231	0.0785	1.148863	7/2	58	0.34203
HL_44467.1	38	109525	11	53377	0.0798	1.683567	8/1	59	0.34203
HL_30128.1	915	108648	411	52977	0.0884	1.085535	9/2	60	0.37282
HL_38138.1	820	108743	367	53021	0.0916	1.089415	6/1	61	0.37987
HL_48254.1	1365	108198	625	52763	0.1012	1.065031	9/2	62	0.41304
HL_05113.1	86	109477	32	53356	0.1122	1.309857	7/1	63	0.44363
HL_56809.1	86	109477	32	53356	0.1122	1.309857	4/1	64	0.44363
HL_42345.1	1066	108497	487	52901	0.1232	1.067269	7/1	65	0.47972
HL_58601.1	3688	105875	1739	51649	0.1282	1.034571	5/2	66	0.49160
HL_23182.1	1162	108401	534	52854	0.1354	1.060984	8/2	67	0.51119
HL_55202.1	14	109549	3	53385	0.1410	2.274131	13/2	68	0.51752
HL_30731.1	1573	107990	730	52658	0.1411	1.050718	4/1	69	0.51752
HL_33451.1	8	109555	1	53387	0.1512	3.898066	11/1	70	0.54663
HL_97270.3	2701	106862	1271	52117	0.1534	1.03642	7/2	71	0.54666
HL_52116.1	753	108810	344	53044	0.1680	1.067096	7/2	72	0.59017
HL_44522.1	163	109400	69	53319	0.1814	1.151329	5/2	73	0.62872
HL_53015.1	544	109019	248	53140	0.2026	1.069217	8/3	74	0.65451
HL_90579.3	440	109123	199	53189	0.2030	1.077716	10/2	75	0.65451
HL_33875.1	675	108888	310	53078	0.2031	1.061393	11/3	76	0.65451
HL_66174.1	980	108583	455	52933	0.2042	1.049975	9/4	77	0.65451
HL_08203.1	55	109508	21	53367	0.2042	1.276306	9/4	78	0.65451
HL_24473.3	4	109559	0	53388	0.2044	Inf	13/4	79	0.65451
HL_57923.1	247	109316	109	53279	0.2106	1.104438	7/2	80	0.66587
HL_93530.1	1008	108555	470	52918	0.2225	1.045481	7/1	81	0.69508
HL_84888.1	439	109124	200	53188	0.2279	1.069861	9/3	82	0.70311
HL_91872.1	449	109114	205	53183	0.2327	1.06754	9/3	83	0.70624
HL_93567.1	1763	107800	833	52555	0.2367	1.031788	7/1	84	0.70624
HL_53789.1	2048	107515	970	52418	0.2373	1.029366	6/1	85	0.70624
HL_63304.1	14	109549	4	53384	0.2469	1.705569	15/1	86	0.72629

Continued on next page

Table G – continued from previous page

ModelID	A	B	C	D	P-value	Odds ratio	Nuc./Bp	Rank	Adj. p-value
HL_17008.1	28	109535	10	53378	0.2540	1.364508	10/2	87	0.72961
HL_20028.1	166	109397	73	53315	0.2553	1.108224	8/1	88	0.72961
HL_82294.3	2977	106586	1420	51968	0.2567	1.022178	7/2	89	0.72961
HL_18156.2	253	109310	114	53274	0.2626	1.081609	7/2	90	0.73819
HL_42094.1	636	108927	296	53092	0.2686	1.04727	5/2	91	0.74667
HL_74686.1	249	109314	114	53274	0.3117	1.06447	10/2	92	0.85055
HL_25175.1	8	109555	2	53386	0.3127	1.949187	16/4	93	0.85055
HL_33640.1	609	108954	287	53101	0.3339	1.034152	8/1	94	0.89877
HL_74465.5	863	108700	411	52977	0.3629	1.023375	9/1	95	0.95886
HL_13707.2	66	109497	29	53359	0.3654	1.109046	8/3	96	0.95886
HL_62970.1	238	109325	111	53277	0.3750	1.044898	13/4	97	0.95886
HL_66880.1	57	109506	25	53363	0.3787	1.111054	11/1	98	0.95886
HL_68081.3	1533	108030	736	52652	0.3789	1.015161	8/2	99	0.95886
HL_27429.3	221	109342	103	53285	0.3790	1.045617	9/1	100	0.95886
HL_82288.1	574	108989	273	53115	0.3859	1.02469	6/1	101	0.96660
HL_65347.1	513	109050	245	53143	0.4143	1.020417	10/3	102	1.00000
HL_57217.1	434	109129	207	53181	0.4179	1.021745	8/2	103	1.00000
HL_78731.1	843	108720	405	52983	0.4201	1.014385	9/1	104	1.00000
HL_87223.1	135	109428	63	53325	0.4210	1.044228	7/1	105	1.00000
HL_44390.1	13	109550	5	53383	0.4325	1.266921	11/3	106	1.00000
HL_96915.1	602	108961	289	53099	0.4328	1.01512	9/2	107	1.00000
HL_11547.2	306	109257	146	53242	0.4388	1.021363	8/1	108	1.00000
HL_42436.1	2	109561	0	53388	0.4521	Inf	15/3	109	1.00000
HL_15603.2	77	109486	36	53352	0.4629	1.042264	12/1	110	1.00000
HL_23195.1	4	109559	1	53387	0.4722	1.949152	17/3	111	1.00000
HL_94578.1	365	109198	176	53212	0.4747	1.010594	6/3	112	1.00000
HL_66948.1	482	109081	233	53155	0.4777	1.008062	7/2	113	1.00000
HL_12706.1	1593	107970	774	52614	0.4833	1.002936	4/2	114	1.00000
HL_20920.1	8	109555	3	53385	0.4889	1.299487	11/3	115	1.00000
HL_72543.2	308	109255	149	53239	0.4932	1.007288	10/3	116	1.00000
HL_62967.1	9344	100219	4554	48834	0.5084	0.9997828	5/3	117	1.00000
HL_42969.1	24	109539	11	53377	0.5131	1.063172	10/1	118	1.00000
HL_83865.1	5284	104279	2576	50812	0.5134	0.9995082	6/1	119	1.00000
HL_28676.1	1317	108246	643	52745	0.5270	0.9980315	6/1	120	1.00000
HL_01926.3	1067	108496	521	52867	0.5273	0.9979244	8/3	121	1.00000
HL_76585.1	475	109088	232	53156	0.5295	0.9976563	9/2	122	1.00000
HL_19226.1	524	109039	256	53132	0.5306	0.9973914	5/1	123	1.00000
HL_11376.1	23882	85681	11647	41741	0.5361	0.9989297	5/1	124	1.00000
HL_24544.2	2126	107437	1039	52349	0.5396	0.9970162	8/3	125	1.00000
HL_65249.1	8634	100929	4214	49174	0.5400	0.9982448	6/2	126	1.00000
HL_93263.1	328	109235	161	53227	0.5516	0.9927003	2/1	127	1.00000
HL_21545.1	2149	107414	1056	52332	0.5974	0.9914662	6/1	128	1.00000
HL_06997.2	16	109547	8	53380	0.6177	0.9745379	12/2	129	1.00000
HL_93771.1	2734	106829	1347	52041	0.6383	0.9887479	6/1	130	1.00000
HL_86398.1	82	109481	42	53346	0.6436	0.9513231	11/3	131	1.00000
HL_46175.2	484	109079	242	53146	0.6449	0.9744286	10/2	132	1.00000
HL_11509.2	407	109156	204	53184	0.6469	0.9720451	10/3	133	1.00000
HL_55718.1	8095	101468	3973	49415	0.6542	0.9922638	3/1	134	1.00000
HL_85044.1	21	109542	11	53377	0.6556	0.9302528	11/2	135	1.00000
HL_17537.1	181	109382	92	53296	0.6556	0.9586119	8/1	136	1.00000
HL_79902.1	195	109368	99	53289	0.6558	0.9597349	8/3	137	1.00000
HL_88960.1	402	109161	202	53186	0.6571	0.969597	8/2	138	1.00000
HL_62880.1	2961	106602	1461	51927	0.6607	0.9872195	6/1	139	1.00000
HL_85534.1	335	109228	169	53219	0.6628	0.9658319	7/3	140	1.00000
HL_20806.1	1	109562	0	53388	0.6724	Inf	11/2	141	1.00000
HL_98577.1	1	109562	0	53388	0.6724	Inf	13/2	142	1.00000
HL_26495.1	375	109188	190	53198	0.6871	0.9616226	8/1	143	1.00000
HL_94697.1	139	109424	72	53316	0.6917	0.9406496	11/2	144	1.00000
HL_50059.1	620	108943	312	53076	0.6926	0.9681637	7/4	145	1.00000

Continued on next page

Table G – continued from previous page

ModelID	A	B	C	D	P-value	Odds ratio	Nuc./Bp	Rank	Adj. p-value
HL_13786.1	2675	106888	1325	52063	0.6957	0.9833411	5/1	146	1.00000
HL_38897.1	113	109450	59	53329	0.6979	0.9332003	7/1	147	1.00000
HL_58083.2	263	109300	135	53253	0.7088	0.949175	9/2	148	1.00000
HL_60200.1	1089	108474	546	52842	0.7174	0.9715771	8/2	149	1.00000
HL_95049.1	687	108876	348	53040	0.7346	0.9617354	8/1	150	1.00000
HL_68733.1	191	109372	100	53288	0.7418	0.9305881	8/1	151	1.00000
HL_46570.1	937	108626	473	52915	0.7452	0.9650137	8/1	152	1.00000
HL_59182.1	2	109561	1	53387	0.7483	0.9745412	15/2	153	1.00000
HL_32644.2	342	109221	177	53211	0.7585	0.9413447	10/3	154	1.00000
HL_24108.1	63	109500	35	53353	0.7691	0.877043	8/2	155	1.00000
HL_54751.2	59	109504	33	53355	0.7738	0.8711387	11/2	156	1.00000
HL_29196.1	570	108993	293	53095	0.7834	0.9476821	6/1	157	1.00000
HL_58223.1	166	109397	89	53299	0.7875	0.908727	5/1	158	1.00000
HL_96994.1	11	109552	7	53381	0.7923	0.7657342	11/1	159	1.00000
HL_82538.1	21233	88330	10437	42951	0.7936	0.9892345	5/1	160	1.00000
HL_19528.1	504	109059	261	53127	0.7995	0.9406854	9/3	161	1.00000
HL_23129.1	50	109513	29	53359	0.8080	0.8400808	9/2	162	1.00000
HL_07915.1	554	109009	287	53101	0.8111	0.9403051	6/1	163	1.00000
HL_87844.2	2332	107231	1172	52216	0.8135	0.9689421	6/2	164	1.00000
HL_04194.1	322	109241	170	53218	0.8149	0.9227434	7/3	165	1.00000
HL_85018.1	83	109480	47	53341	0.8210	0.8604222	10/1	166	1.00000
HL_84289.1	27	109536	17	53371	0.8393	0.7738897	11/2	167	1.00000
HL_62881.1	494	109069	260	53128	0.8524	0.9255007	10/4	168	1.00000
HL_76679.1	3499	106064	1758	51630	0.8598	0.9688864	5/2	169	1.00000
HL_72498.12	791	108772	411	52977	0.8622	0.9373582	7/2	170	1.00000
HL_77692.1	598	108965	314	53074	0.8665	0.927614	10/4	171	1.00000
HL_45411.1	677	108886	354	53034	0.8668	0.9314694	8/2	172	1.00000
HL_67761.1	4056	105507	2039	51349	0.8818	0.9681542	6/1	173	1.00000
HL_64371.2	27	109536	18	53370	0.8825	0.7308323	12/2	174	1.00000
HL_88311.2	541	109022	287	53101	0.8854	0.9181322	7/3	175	1.00000
HL_82243.1	1189	108374	616	52772	0.8971	0.9398969	9/1	176	1.00000
HL_39486.1	4127	105436	2079	51309	0.8986	0.9660417	7/1	177	1.00000
HL_39895.6	5389	104174	2704	50684	0.9008	0.9696169	5/2	178	1.00000
HL_80492.2	68	109495	42	53346	0.9042	0.7888252	8/4	179	1.00000
HL_25195.1	3	109560	3	53385	0.9054	0.4872563	12/2	180	1.00000
HL_30366.1	257	109306	144	53244	0.9181	0.8693638	11/5	181	1.00000
HL_69403.1	176	109387	102	53286	0.9269	0.8405565	9/3	182	1.00000
HL_65802.1	255	109308	144	53244	0.9285	0.8625837	11/3	183	1.00000
HL_35200.1	1558	108005	808	52580	0.9289	0.9387143	6/1	184	1.00000
HL_42553.1	15	109548	12	53376	0.9302	0.6090377	10/3	185	1.00000
HL_70912.1	586	108977	316	53072	0.9316	0.9031151	7/1	186	1.00000
HL_91226.3	1384	108179	722	52666	0.9352	0.9332265	8/1	187	1.00000
HL_38898.1	329	109234	184	53204	0.9383	0.8709007	8/1	188	1.00000
HL_39872.2	644	108919	347	53041	0.9387	0.9037876	8/2	189	1.00000
HL_84768.1	502	109061	274	53114	0.9391	0.8922679	4/1	190	1.00000
HL_18781.4	1872	107691	969	52419	0.9403	0.9403553	8/2	191	1.00000
HL_80459.3	14823	94740	7373	46015	0.9405	0.9764514	5/1	192	1.00000
HL_47784.2	604	108959	327	53061	0.9416	0.8995052	11/2	193	1.00000
HL_23290.1	1127	108436	594	52794	0.9427	0.9237397	7/1	194	1.00000
HL_68697.2	12017	97546	5997	47391	0.9460	0.9735069	6/1	195	1.00000
HL_91613.1	11	109552	10	53378	0.9505	0.5359408	10/3	196	1.00000
HL_33402.3	1587	107976	830	52558	0.9537	0.9307034	7/2	197	1.00000
HL_65924.1	2	109561	3	53385	0.9575	0.3248442	14/4	198	1.00000
HL_56775.1	595	108968	329	53059	0.9694	0.8806118	7/2	199	1.00000
HL_16770.1	164	109399	101	53287	0.9716	0.7909402	10/2	200	1.00000
HL_39942.2	881	108682	478	52910	0.9726	0.8972851	7/1	201	1.00000
HL_38130.2	25	109538	21	53367	0.9761	0.5799887	11/1	202	1.00000
HL_19132.1	309	109254	181	53207	0.9775	0.8314178	8/3	203	1.00000
HL_78228.1	666	108897	372	53016	0.9837	0.8716184	12/2	204	1.00000

Continued on next page

Table G – continued from previous page

ModelID	A	B	C	D	P-value	Odds ratio	Nuc./Bp	Rank	Adj. p-value
HL_60203.1	162	109401	103	53285	0.9846	0.7660876	5/2	205	1.00000
HL_39422.1	541	109022	308	53080	0.9864	0.8552022	7/1	206	1.00000
HL_66467.1	2305	107258	1215	52173	0.9878	0.9228091	5/1	207	1.00000
HL_41833.2	51	109512	40	53348	0.9904	0.621097	11/4	208	1.00000
HL_48480.1	640	108923	365	53023	0.9923	0.8535667	7/1	209	1.00000
HL_49492.1	2	109561	5	53383	0.9938	0.1949056	14/4	210	1.00000
HL_64543.2	1458	108105	793	52595	0.9941	0.8945105	9/2	211	1.00000
HL_86123.2	927	108636	519	52869	0.9947	0.8692485	10/2	212	1.00000
HL_08382.2	15222	94341	7681	45707	0.9965	0.9601591	6/1	213	1.00000
HL_49036.3	23814	85749	11922	41466	0.9968	0.9659612	6/2	214	1.00000
HL_52574.3	4400	105163	2298	51090	0.9971	0.9302002	7/1	215	1.00000
HL_37962.1	246	109317	159	53229	0.9975	0.7533249	8/2	216	1.00000
HL_61547.5	16430	93133	8292	45096	0.9977	0.959443	6/1	217	1.00000
HL_47337.1	4597	104966	2402	50986	0.9977	0.9296183	5/1	218	1.00000
HL_06122.1	1626	107937	897	52491	0.9987	0.8815479	7/3	219	1.00000
HL_98233.1	86	109477	69	53319	0.9991	0.6070172	8/1	220	1.00000
HL_68579.1	346	109217	221	53167	0.9992	0.7621725	6/2	221	1.00000
HL_99633.1	514	109049	314	53074	0.9992	0.7967206	8/3	222	1.00000
HL_34440.1	2062	107501	1130	52258	0.9993	0.8870608	6/1	223	1.00000
HL_59610.1	1643	107920	916	52472	0.9995	0.872111	5/1	224	1.00000
HL_49210.1	702	108861	420	52968	0.9996	0.8132785	7/2	225	1.00000
HL_62228.3	6568	102995	3427	49961	0.9996	0.929683	6/2	226	1.00000
HL_99779.1	4567	104996	2444	50944	0.9999	0.9066755	5/1	227	1.00000
HL_27271.2	22446	87117	11421	41967	1.0000	0.9467607	6/1	228	1.00000
HL_55272.1	2268	107295	1283	52105	1.0000	0.8584628	10/4	229	1.00000
HL_67000.1	6416	103147	3426	49962	1.0000	0.9071141	5/2	230	1.00000
HL_56824.2	11571	97992	6029	47359	1.0000	0.9275523	5/1	231	1.00000
HL_95720.1	1039	108524	639	52749	1.0000	0.7903445	8/3	232	1.00000
HL_52540.1	4722	104841	2575	50813	1.0000	0.8887819	4/2	233	1.00000
HL_91693.1	11115	98448	5826	47562	1.0000	0.9217078	5/1	234	1.00000
HL_10116.1	0	109563	0	53388	1.0000	0	18/5	235	1.00000
HL_19221.2	6314	103249	3607	49781	1.0000	0.8440003	6/2	236	1.00000
HL_19399.1	0	109563	0	53388	1.0000	0	16/2	237	1.00000
HL_19452.1	0	109563	2	53386	1.0000	0	11/3	238	1.00000
HL_25124.2	4382	105181	2493	50895	1.0000	0.8505381	5/2	239	1.00000
HL_25197.1	0	109563	1	53387	1.0000	0	15/4	240	1.00000
HL_29831.1	6972	102591	3899	49489	1.0000	0.8625975	6/2	241	1.00000
HL_45358.1	0	109563	0	53388	1.0000	0	13/2	242	1.00000
HL_57843.1	3467	106096	1966	51422	1.0000	0.8547237	5/2	243	1.00000
HL_67042.12	14496	95067	7801	45587	1.0000	0.8910694	6/2	244	1.00000
HL_70420.2	0	109563	0	53388	1.0000	0	14/8	245	1.00000
HL_76036.3	11702	97861	6499	46889	1.0000	0.8627389	5/1	246	1.00000
HL_76766.4	8628	100935	4796	48592	1.0000	0.8660805	5/2	247	1.00000
HL_79038.1	10292	99271	5684	47704	1.0000	0.8701258	5/1	248	1.00000
HL_84353.1	9138	100425	5037	48351	1.0000	0.8734671	6/2	249	1.00000
HL_87136.2	1242	108321	821	52567	1.0000	0.7341161	8/1	250	1.00000
HL_90102.1	0	109563	0	53388	1.0000	0	19/3	251	1.00000
HL_97784.1	0	109563	2	53386	1.0000	0	15/1	252	1.00000
HL_97971.1	6475	103088	3721	49667	1.0000	0.8383916	6/1	253	1.00000

Table H: Results of Fisher's exact test according to FET scheme (see Supplementary Material S6) for JAR3D HL module predictions for SISSIz and original data of order R. Brown highlighted rows have a significant p-value after multiple testing correction using the Benjamini-Hochberg procedure at a 5% false discovery rate (adjusted p-value). "Nuc./Bp" denotes the total strand length and the number of base pairs.

ModelID	A	B	C	D	P-value	Odds ratio	Nuc./Bp	Rank	Adj. p-value
HL_75759.4	5824	104977	2287	51101	3.771924e-18	1.239581	3/1	1	9.54296772e-16
HL_35865.1	5987	104814	2436	50952	1.55991e-13	1.194715	5/1	2	1.97328615e-11
HL_06643.3	5399	105402	2233	51155	1.891175e-10	1.173429	5/2	3	1.59489091667e-08
HL_87554.1	1906	108895	705	52683	4.169671e-10	1.308007	5/1	4	2.6373169075e-08
HL_42677.2	4647	106154	1935	51453	1.48667e-08	1.164019	3/1	5	7.5225502e-07
HL_74559.1	2845	107956	1138	52250	2.970469e-08	1.209957	4/1	6	1.25254776167e-06
HL_90542.1	1016	109785	358	53030	9.929761e-08	1.370871	7/2	7	3.58889933285714e-06
HL_97499.1	2104	108697	823	52565	1.183941e-07	1.236269	4/1	8	3.7442134125e-06
HL_30008.1	2054	108747	807	52581	2.835842e-07	1.230629	5/1	9	7.97186695555e-06
HL_10378.1	1731	109070	674	52714	9.084284e-07	1.241211	4/1	10	2.298323852e-05
HL_63941.1	4471	106330	1903	51485	1.717646e-06	1.137593	4/1	11	3.9505858e-05
HL_75579.2	16365	94436	7437	45951	2.927149e-06	1.070728	3/1	12	6.17140580833e-05
HL_75850.1	4014	106787	1705	51683	4.125485e-06	1.139406	5/1	13	8.0288285e-05
HL_59604.1	2304	108497	940	52448	6.254898e-06	1.184838	3/1	14	0.00011
HL_74505.1	4164	106637	1779	51609	7.052144e-06	1.132789	4/1	15	0.00012
HL_34027.2	7211	103590	3180	50208	8.167672e-06	1.099075	6/1	16	0.00012
HL_57514.2	9424	101377	4207	49181	8.206241e-06	1.086736	4/1	17	0.00012
HL_05361.1	566	110235	192	53196	9.273833e-06	1.422578	6/2	18	0.00013
HL_65071.1	975	109826	366	53022	1.8207e-05	1.286049	4/1	19	0.00024
HL_86115.1	4160	106641	1795	51593	3.270497e-05	1.121225	4/2	20	0.00041
HL_42077.2	15215	95586	6949	46439	3.471757e-05	1.063753	5/2	21	0.00042
HL_03785.1	3876	106925	1675	51713	7.347684e-05	1.119146	5/1	22	0.00084
HL_17790.1	2200	108601	916	52472	8.366967e-05	1.160422	3/1	23	0.00092
HL_99207.1	1852	108949	764	52624	0.0001	1.170854	7/3	24	0.00136
HL_59225.1	1998	108803	838	52550	0.0003	1.15154	5/1	25	0.00331
HL_48039.2	18707	92094	8664	44724	0.0004	1.048569	4/1	26	0.00416
HL_48116.2	8869	101932	4028	49360	0.0006	1.066232	6/1	27	0.00555
HL_19905.3	8091	102710	3670	49718	0.0008	1.067183	4/1	28	0.00738
HL_26579.1	682	110119	262	53126	0.0009	1.255784	5/2	29	0.00748
HL_00721.1	1336	109465	552	52836	0.0011	1.168199	2/1	30	0.00936
HL_35619.2	1475	109326	615	52773	0.0012	1.157713	5/2	31	0.00982
HL_17723.4	1761	109040	745	52643	0.0013	1.141181	7/1	32	0.01065
HL_01418.1	1593	109208	671	52717	0.0016	1.146003	5/1	33	0.01250
HL_68435.1	852	109949	343	53045	0.0024	1.198371	7/2	34	0.01747
HL_99584.1	1345	109456	563	52825	0.0024	1.152948	4/1	35	0.01747
HL_45018.3	874	109927	358	53030	0.0048	1.177714	10/4	36	0.03360
HL_34108.2	9796	101005	4515	48873	0.0049	1.049829	4/1	37	0.03372
HL_98523.2	4991	105810	2257	51131	0.0053	1.0686	6/1	38	0.03547
HL_21675.3	7572	103229	3470	49918	0.0057	1.055208	5/2	39	0.03713
HL_95716.1	5951	104850	2711	50677	0.0065	1.060973	5/1	40	0.04129
HL_18587.1	842	109959	348	53040	0.0081	1.16708	8/2	41	0.04981
HL_73465.1	28	110773	4	53384	0.0090	3.373325	11/2	42	0.05338
HL_00090.1	757	110044	311	53077	0.0091	1.174005	11/1	43	0.05338
HL_24707.1	1714	109087	748	52640	0.0117	1.105733	4/2	44	0.06728
HL_72273.1	753	110048	312	53076	0.0127	1.163997	6/2	45	0.07061
HL_11376.1	24714	86087	11647	41741	0.0128	1.028858	5/1	46	0.07061
HL_46489.2	951	109850	403	52985	0.0156	1.138217	9/2	47	0.08393
HL_50312.1	4368	106433	1991	51397	0.0185	1.059434	6/1	48	0.09752
HL_33277.1	1597	109204	701	52687	0.0197	1.099133	7/1	49	0.10196
HL_98833.3	746	110055	317	53071	0.0315	1.134812	8/2	50	0.15958

Continued on next page

Table H – continued from previous page

ModelID	A	B	C	D	P-value	Odds ratio	Nuc./Bp	Rank	Adj. p-value
HL_24711.1	732	110069	311	53077	0.0327	1.134982	7/2	51	0.16077
HL_58601.1	3804	106997	1739	51649	0.0330	1.055923	5/2	52	0.16077
HL_44467.1	41	110760	11	53377	0.0509	1.796226	8/1	53	0.24304
HL_78420.1	552	110249	234	53154	0.0530	1.137317	9/1	54	0.24842
HL_30731.1	1627	109174	730	52658	0.0554	1.075001	4/1	55	0.25308
HL_41827.1	1133	109668	501	52887	0.0562	1.090586	7/2	56	0.25308
HL_36842.1	1999	108802	904	52484	0.0570	1.066679	8/2	57	0.25308
HL_53789.1	2136	108665	970	52418	0.0632	1.062233	6/1	58	0.27556
HL_73972.1	955	109846	421	52967	0.0665	1.093808	6/3	59	0.28512
HL_78507.1	2128	108673	968	52420	0.0691	1.060403	5/1	60	0.29149
HL_42345.1	1095	109706	487	52901	0.0729	1.084221	7/1	61	0.30228
HL_27397.1	374	110427	157	53231	0.0789	1.148306	7/2	62	0.31490
HL_30128.1	928	109873	411	52977	0.0803	1.088683	9/2	63	0.31490
HL_56809.1	90	110711	32	53356	0.0810	1.355489	4/1	64	0.31490
HL_48254.1	1388	109413	625	52763	0.0817	1.07095	9/2	65	0.31490
HL_78361.1	641	110160	279	53109	0.0821	1.107634	8/2	66	0.31490
HL_38138.1	831	109970	367	53021	0.0857	1.09171	6/1	67	0.32357
HL_66877.1	166	110635	65	53323	0.0871	1.230854	11/4	68	0.32421
HL_82538.1	21968	88833	10437	42951	0.0941	1.017687	5/1	69	0.34518
HL_97270.3	2756	108045	1271	52117	0.0980	1.045943	7/2	70	0.35416
HL_05113.1	88	110713	32	53356	0.1004	1.325353	7/1	71	0.35792
HL_23182.1	1185	109616	534	52854	0.1025	1.069992	8/2	72	0.36010
HL_62967.1	9659	101142	4554	48834	0.1045	1.024071	5/3	73	0.36219
HL_84888.1	462	110339	200	53188	0.1094	1.113511	9/3	74	0.37420
HL_91872.1	471	110330	205	53183	0.1191	1.107502	9/3	75	0.40191
HL_44522.1	169	110632	69	53319	0.1369	1.180411	5/2	76	0.45589
HL_93567.1	1808	108993	833	52555	0.1451	1.046571	7/1	77	0.47310
HL_55202.1	14	110787	3	53385	0.1459	2.248719	13/2	78	0.47310
HL_93530.1	1035	109766	470	52918	0.1484	1.061641	7/1	79	0.47457
HL_52116.1	765	110036	344	53044	0.1501	1.072022	7/2	80	0.47457
HL_55718.1	8396	102405	3973	49415	0.1669	1.019745	3/1	81	0.51589
HL_65249.1	8900	101901	4214	49174	0.1672	1.019184	6/2	82	0.51589
HL_66174.1	998	109803	455	52933	0.1701	1.05738	9/4	83	0.51849
HL_83865.1	5466	105335	2576	50812	0.1740	1.02357	6/1	84	0.52408
HL_53015.1	553	110248	248	53140	0.1832	1.07479	8/3	85	0.54538
HL_08203.1	56	110745	21	53367	0.1957	1.284995	9/4	86	0.56254
HL_57923.1	251	110550	109	53279	0.1978	1.109795	7/2	87	0.56254
HL_33875.1	683	110118	310	53078	0.2003	1.061976	11/3	88	0.56254
HL_82294.3	3028	107773	1420	51968	0.2012	1.028238	7/2	89	0.56254
HL_12706.1	1667	109134	774	52614	0.2016	1.038316	4/2	90	0.56254
HL_90579.3	445	110356	199	53189	0.2023	1.077785	10/2	91	0.56254
HL_24473.3	4	110797	0	53388	0.2074	Inf	13/4	92	0.56804
HL_33451.1	7	110794	1	53387	0.2088	3.372874	11/1	93	0.56804
HL_66880.1	64	110737	25	53363	0.2198	1.233608	11/1	94	0.59149
HL_17008.1	29	110772	10	53378	0.2309	1.39745	10/2	95	0.61487
HL_18156.2	257	110544	114	53274	0.2491	1.086444	7/2	96	0.65559
HL_20028.1	168	110633	73	53315	0.2531	1.109046	8/1	97	0.65559
HL_63304.1	14	110787	4	53384	0.2539	1.68651	15/1	98	0.65559
HL_74686.1	255	110546	114	53274	0.2722	1.07797	10/2	99	0.69572
HL_13707.2	70	110731	29	53359	0.2847	1.163145	8/3	100	0.71353
HL_74465.5	884	109917	411	52977	0.2848	1.036632	9/1	101	0.71353
HL_42094.1	639	110162	296	53092	0.3001	1.040403	5/2	102	0.74447
HL_68081.3	1562	109239	736	52652	0.3160	1.022935	8/2	103	0.76771
HL_62970.1	245	110556	111	53277	0.3166	1.063653	13/4	104	0.76771
HL_25175.1	8	110793	2	53386	0.3186	1.927407	16/4	105	0.76771
HL_33640.1	617	110184	287	53101	0.3243	1.036044	8/1	106	0.77406
HL_78731.1	863	109938	405	52983	0.3421	1.026963	9/1	107	0.80896
HL_65347.1	526	110275	245	53143	0.3457	1.034613	10/3	108	0.80985
HL_66948.1	499	110302	233	53155	0.3619	1.03203	7/2	109	0.83508

Continued on next page

Table H – continued from previous page

ModelID	A	B	C	D	P-value	Odds ratio	Nuc./Bp	Rank	Adj. p-value
HL_82288.1	583	110218	273	53115	0.3631	1.029159	6/1	110	0.83508
HL_23195.1	5	110796	1	53387	0.3675	2.409236	17/3	111	0.83763
HL_44390.1	14	110787	5	53383	0.3805	1.349223	11/3	112	0.85296
HL_93771.1	2825	107976	1347	52041	0.3813	1.010809	6/1	113	0.85296
HL_15603.2	81	110720	36	53352	0.3843	1.084192	12/1	114	0.85296
HL_11547.2	312	110489	146	53242	0.4065	1.029792	8/1	115	0.89423
HL_87223.1	137	110664	63	53325	0.4118	1.047862	7/1	116	0.89807
HL_27429.3	221	110580	103	53285	0.4156	1.033885	9/1	117	0.89859
HL_94578.1	373	110428	176	53212	0.4291	1.021252	6/3	118	0.92003
HL_57217.1	437	110364	207	53181	0.4381	1.017289	8/2	119	0.92758
HL_96915.1	608	110193	289	53099	0.4400	1.013773	9/2	120	0.92758
HL_13786.1	2764	108037	1325	52063	0.4458	1.005261	5/1	121	0.93209
HL_42436.1	2	110799	0	53388	0.4554	Inf	15/3	122	0.94441
HL_28676.1	1341	109460	643	52745	0.4699	1.004949	6/1	123	0.95848
HL_21545.1	2199	108602	1056	52332	0.4722	1.003439	6/1	124	0.95848
HL_72543.2	313	110488	149	53239	0.4736	1.01222	10/3	125	0.95848
HL_42969.1	25	110776	11	53377	0.4792	1.095102	10/1	126	0.96224
HL_20920.1	8	110793	3	53385	0.4961	1.284872	11/3	127	0.98823
HL_62880.1	3033	107768	1461	51927	0.5036	1.000272	6/1	128	0.99535
HL_01926.3	1081	109720	521	52867	0.5138	0.9997379	8/3	129	1.00000
HL_24544.2	2152	108649	1039	52349	0.5299	0.9979514	8/3	130	1.00000
HL_68697.2	12432	98369	5997	47391	0.5342	0.9987218	6/1	131	1.00000
HL_93263.1	331	110470	161	53227	0.5604	0.9905804	2/1	132	1.00000
HL_88960.1	414	110387	202	53186	0.5771	0.9874749	8/2	133	1.00000
HL_67761.1	4210	106591	2039	51349	0.5830	0.9946629	6/1	134	1.00000
HL_50059.1	639	110162	312	53076	0.5913	0.9867566	7/4	135	1.00000
HL_19226.1	522	110279	256	53132	0.6081	0.9824022	5/1	136	1.00000
HL_76679.1	3619	107182	1758	51630	0.6181	0.9916284	5/2	137	1.00000
HL_86398.1	84	110717	42	53346	0.6183	0.9636644	11/3	138	1.00000
HL_11509.2	414	110387	204	53184	0.6217	0.9777458	10/3	139	1.00000
HL_38897.1	118	110683	59	53329	0.6258	0.9636532	7/1	140	1.00000
HL_85534.1	342	110459	169	53219	0.6259	0.9749803	7/3	141	1.00000
HL_76585.1	470	110331	232	53156	0.6354	0.9760143	9/2	142	1.00000
HL_46175.2	490	110311	242	53146	0.6400	0.9754919	10/2	143	1.00000
HL_87844.2	2401	108400	1172	52216	0.6510	0.9868163	6/2	144	1.00000
HL_79902.1	197	110604	99	53289	0.6590	0.9587398	8/3	145	1.00000
HL_85044.1	21	110780	11	53377	0.6667	0.9198577	11/2	146	1.00000
HL_20806.1	1	110800	0	53388	0.6748	Inf	11/2	147	1.00000
HL_98577.1	1	110800	0	53388	0.6748	Inf	13/2	148	1.00000
HL_06997.2	15	110786	8	53380	0.6823	0.9034351	12/2	149	1.00000
HL_46570.1	957	109844	473	52915	0.6863	0.9746392	8/1	150	1.00000
HL_24108.1	67	110734	35	53353	0.6923	0.9223288	8/2	151	1.00000
HL_29196.1	588	110213	293	53095	0.6950	0.9668116	6/1	152	1.00000
HL_68733.1	196	110605	100	53288	0.7030	0.9443027	8/1	153	1.00000
HL_95049.1	699	110102	348	53040	0.7040	0.9676502	8/1	154	1.00000
HL_60200.1	1102	109699	546	52842	0.7136	0.972197	8/2	155	1.00000
HL_94697.1	139	110662	72	53316	0.7183	0.930127	11/2	156	1.00000
HL_39895.6	5538	105263	2704	50684	0.7232	0.9861402	5/2	157	1.00000
HL_32644.2	349	110452	177	53211	0.7278	0.9499055	10/3	158	1.00000
HL_80459.3	15181	95620	7373	46015	0.7289	0.990845	5/1	159	1.00000
HL_17537.1	178	110623	92	53296	0.7312	0.9321422	8/1	160	1.00000
HL_19528.1	518	110283	261	53127	0.7361	0.9560845	9/3	161	1.00000
HL_49036.3	24585	86216	11922	41466	0.7443	0.991801	6/2	162	1.00000
HL_59182.1	2	110799	1	53387	0.7516	0.9636914	15/2	163	1.00000
HL_58083.2	262	110539	135	53253	0.7552	0.9349682	9/2	164	1.00000
HL_07915.1	567	110234	287	53101	0.7644	0.951675	6/1	165	1.00000
HL_54751.2	60	110741	33	53355	0.7666	0.8760075	11/2	166	1.00000
HL_26495.1	371	110430	190	53198	0.7680	0.9406523	8/1	167	1.00000
HL_23129.1	52	110749	29	53359	0.7752	0.863929	9/2	168	1.00000

Continued on next page

Table H – continued from previous page

ModelID	A	B	C	D	P-value	Odds ratio	Nuc./Bp	Rank	Adj. p-value
HL_08382.2	15785	95016	7681	45707	0.7799	0.9885783	6/1	169	1.00000
HL_58223.1	168	110633	89	53299	0.7863	0.9094009	5/1	170	1.00000
HL_84289.1	29	110772	17	53371	0.7901	0.8219281	11/2	171	1.00000
HL_96994.1	11	110790	7	53381	0.7989	0.7571199	11/1	172	1.00000
HL_04194.1	327	110474	170	53218	0.8036	0.9266128	7/3	173	1.00000
HL_61547.5	17030	93771	8292	45096	0.8043	0.9876949	6/1	174	1.00000
HL_39486.1	4220	106581	2079	51309	0.8048	0.9771567	7/1	175	1.00000
HL_85018.1	83	110718	47	53341	0.8365	0.8508035	10/1	176	1.00000
HL_35200.1	1606	109195	808	52580	0.8488	0.9570911	6/1	177	1.00000
HL_72498.12	802	109999	411	52977	0.8532	0.9397921	7/2	178	1.00000
HL_64371.2	28	110773	18	53370	0.8669	0.7494343	12/2	179	1.00000
HL_62881.1	497	110304	260	53128	0.8676	0.9206965	10/4	180	1.00000
HL_23290.1	1164	109637	594	52794	0.8789	0.9436142	7/1	181	1.00000
HL_88311.2	548	110253	287	53101	0.8815	0.9196281	7/3	182	1.00000
HL_77692.1	601	110200	314	53074	0.8849	0.9218202	10/4	183	1.00000
HL_47784.2	625	110176	327	53061	0.8931	0.920496	11/2	184	1.00000
HL_45411.1	678	110123	354	53034	0.8962	0.9223674	8/2	185	1.00000
HL_38898.1	341	110460	184	53204	0.9004	0.8926443	8/1	186	1.00000
HL_18781.4	1911	108890	969	52419	0.9073	0.9493756	8/2	187	1.00000
HL_25195.1	3	110798	3	53385	0.9077	0.4818127	12/2	188	1.00000
HL_91226.3	1411	109390	722	52666	0.9105	0.9408992	8/1	189	1.00000
HL_84768.1	513	110288	274	53114	0.9214	0.9016742	4/1	190	1.00000
HL_33402.3	1622	109179	830	52558	0.9250	0.9407475	7/2	191	1.00000
HL_82243.1	1192	109609	616	52772	0.9252	0.931652	9/1	192	1.00000
HL_30366.1	258	110543	144	53244	0.9283	0.8629817	11/5	193	1.00000
HL_69403.1	177	110624	102	53286	0.9330	0.8358812	9/3	194	1.00000
HL_91613.1	12	110789	10	53378	0.9330	0.5781475	10/3	195	1.00000
HL_80492.2	65	110736	42	53346	0.9424	0.7455251	8/4	196	1.00000
HL_66467.1	2388	108413	1215	52173	0.9426	0.9458515	5/1	197	1.00000
HL_70912.1	588	110213	316	53072	0.9452	0.8960357	7/1	198	1.00000
HL_47337.1	4794	106007	2402	50986	0.9463	0.959945	5/1	199	1.00000
HL_42553.1	14	110787	12	53376	0.9517	0.5620746	10/3	200	1.00000
HL_39872.2	644	110157	347	53041	0.9565	0.8936317	8/2	201	1.00000
HL_56775.1	608	110193	329	53059	0.9581	0.8898474	7/2	202	1.00000
HL_65924.1	2	110799	3	53385	0.9586	0.3212147	14/4	203	1.00000
HL_65802.1	249	110552	144	53244	0.9632	0.8328159	11/3	204	1.00000
HL_19132.1	315	110486	181	53207	0.9730	0.8381099	8/3	205	1.00000
HL_16770.1	163	110638	101	53287	0.9792	0.7773183	10/2	206	1.00000
HL_39942.2	885	109916	478	52910	0.9793	0.8912406	7/1	207	1.00000
HL_78228.1	678	110123	372	53016	0.9795	0.8774439	12/2	208	1.00000
HL_49492.1	3	110798	5	53383	0.9827	0.2890809	14/4	209	1.00000
HL_38130.2	24	110777	21	53367	0.9836	0.5505469	11/1	210	1.00000
HL_27271.2	23190	87611	11421	41967	0.9846	0.9726052	6/1	211	1.00000
HL_62228.3	6805	103996	3427	49961	0.9856	0.9539571	6/2	212	1.00000
HL_39422.1	546	110255	308	53080	0.9875	0.8534545	7/1	213	1.00000
HL_60203.1	161	110640	103	53285	0.9891	0.7527761	5/2	214	1.00000
HL_52574.3	4502	106299	2298	51090	0.9894	0.941592	7/1	215	1.00000
HL_86123.2	950	109851	519	52869	0.9900	0.8809612	10/2	216	1.00000
HL_41833.2	51	110750	40	53348	0.9916	0.6141556	11/4	217	1.00000
HL_64543.2	1479	109322	793	52595	0.9930	0.8972929	9/2	218	1.00000
HL_48480.1	646	110155	365	53023	0.9930	0.8519335	7/1	219	1.00000
HL_34440.1	2140	108661	1130	52258	0.9942	0.9107854	6/1	220	1.00000
HL_56824.2	12033	98768	6029	47359	0.9957	0.9570127	5/1	221	1.00000
HL_59610.1	1691	109110	916	52472	0.9980	0.8877979	5/1	222	1.00000
HL_91693.1	11568	99233	5826	47562	0.9982	0.9516826	5/1	223	1.00000
HL_06122.1	1651	109150	897	52491	0.9983	0.885154	7/3	224	1.00000
HL_37962.1	245	110556	159	53229	0.9984	0.7418576	8/2	225	1.00000
HL_99633.1	528	110273	314	53074	0.9985	0.8093343	8/3	226	1.00000
HL_67000.1	6682	104119	3426	49962	0.9989	0.9359004	5/2	227	1.00000

Continued on next page

Table H – continued from previous page

ModelID	A	B	C	D	P-value	Odds ratio	Nuc./Bp	Rank	Adj. p-value
HL_68579.1	352	110449	221	53167	0.9990	0.76674	6/2	228	1.00000
HL_99779.1	4696	106105	2444	50944	0.9992	0.92254	5/1	229	1.00000
HL_49210.1	718	110083	420	52968	0.9992	0.8225793	7/2	230	1.00000
HL_98233.1	86	110715	69	53319	0.9993	0.6002307	8/1	231	1.00000
HL_52540.1	4940	105861	2575	50813	0.9995	0.9208518	4/2	232	1.00000
HL_55272.1	2293	108508	1283	52105	1.0000	0.8582234	10/4	233	1.00000
HL_95720.1	1061	109740	639	52749	1.0000	0.798135	8/3	234	1.00000
HL_57843.1	3564	107237	1966	51422	1.0000	0.8692862	5/2	235	1.00000
HL_25124.2	4552	106249	2493	50895	1.0000	0.8746496	5/2	236	1.00000
HL_10116.1	0	110801	0	53388	1.0000	0	18/5	237	1.00000
HL_19221.2	6554	104247	3607	49781	1.0000	0.8676902	6/2	238	1.00000
HL_19399.1	0	110801	0	53388	1.0000	0	16/2	239	1.00000
HL_19452.1	0	110801	2	53386	1.0000	0	11/3	240	1.00000
HL_25197.1	0	110801	1	53387	1.0000	0	15/4	241	1.00000
HL_29831.1	7235	103566	3899	49489	1.0000	0.8867061	6/2	242	1.00000
HL_45358.1	0	110801	0	53388	1.0000	0	13/2	243	1.00000
HL_67042.12	15031	95770	7801	45587	1.0000	0.9171711	6/2	244	1.00000
HL_70420.2	0	110801	0	53388	1.0000	0	14/8	245	1.00000
HL_76036.3	12176	98625	6499	46889	1.0000	0.890727	5/1	246	1.00000
HL_76766.4	8971	101830	4796	48592	1.0000	0.8925925	5/2	247	1.00000
HL_79038.1	10721	100080	5684	47704	1.0000	0.8990645	5/1	248	1.00000
HL_84353.1	9487	101314	5037	48351	1.0000	0.8988663	6/2	249	1.00000
HL_87136.2	1265	109536	821	52567	1.0000	0.7394172	8/1	250	1.00000
HL_90102.1	0	110801	0	53388	1.0000	0	19/3	251	1.00000
HL_97784.1	0	110801	2	53386	1.0000	0	15/1	252	1.00000
HL_97971.1	6747	104054	3721	49667	1.0000	0.8654953	6/1	253	1.00000

Table I: Results of Fisher's exact test according to the FET scheme (see Supplementary Material S6) for JAR3D HL module predictions of all JAR3D HL models with mean interior edit distance ≤ 4 and passed cutoff ≥ 50.

Order	Model	A	B	C	D	P-value	Odds ratio
H	all	94208	17852	44755	8633	0.108449	1.017947
L	all	92134	17429	44755	8633	0.08859385	1.0197
R	all	93603	17198	44755	8633	0.0003771921	1.049861
