# Supplementary material for: RNA 3D Modules in Genome-Wide Predictions of RNA 2D Structure
Source: PLoS One. 2015 Oct 28;10(10):e0139900. doi: 10.1371/journal.pone.0139900 (PMC4624896; doi:10.1371/journal.pone.0139900)
Supplement: S4 Text — False discovery rates for individual mRm modules of ordering L (Table A) and of ordering R (Table B), individual JAR3D IL modules of ordering L (Table C) and of ordering H (Table D), and individual JAR3D HL modules of ordering L (Table E) and of ordering R (Table F). (PDF) [file pone.0139900.s008.pdf]

Supplementary Information 4
False discovery rates of alternative approaches L and R.

Table A: False discovery rates (FDR) for individual mRm$_{IL}$ models with mean RMDetect score $\geq$ Q$_{0.75}$ in windows with p-score > 0.9 and 0.25 $\leq$ GC content $\leq$ 0.75 of order L. Status "+" denotes models with adjusted p-value $\leq$ 0.05 and odds ratio $\geq$ 1.0 whereas "*" denotes models with odds ratio $\geq$ 1.0. Models assigned with "-" are neither enriched nor occur at higher rates in the original data. Colored rows have an FDR $\leq \hat{F}_{rnaz}$ and original window count $\geq$ 6. The table is sorted in ascending order according to FDR.

**p-score > 0.9, 0.25 ≤ GC content ≤ 0.75**					
ModelID	# original	# shuffled	FDR (%)	Odds ratio	Stat.
$\hat{F}_{rnaz}$	142 490	66 891	46.9%		
RF00011_1583_1NBS_177_190_209_219	0	0	0.000	0.000	-
RF00177_1133_2VHO_380_389_400_408	1	0	0.000	Inf	*
RF00177_698_2B64_481_491_518_522	1	0	0.000	Inf	*
RF01118_270_2QBC_13_17_35_55	0	0	0.000	0.000	-
RF00177_351_1I95_804_807_829_846	1	0	0.000	Inf	*
RF00177_437_2QBF_488_495_500_509	0	0	0.000	0.000	-
RF00177_551_2UXD_494_500_507_515	0	0	0.000	0.000	-
RF00177_171_1HR0_804_807_829_846	1	0	0.000	Inf	*
RF00177_1470_1XNQ_1103_1111_1121_1128	0	0	0.000	0.000	-
RF00177_1369_2GY9_669_675_686_695	2	0	0.000	Inf	*
RF00177_524_1VOV_400_408_417_424	3	0	0.000	Inf	*
RF00177_942_1N36_137_143_158_165	13	2	15.385	1.584	*
RF00177_857_3BBN_130_136_151_158	13	2	15.385	1.584	*
RF00177_1012_3FIH_120_126_141_148	13	2	15.385	1.267	*
RF00177_751_1IBL_660_665_676_684	4	1	25.000	2.924	*
RF00177_243_2QBB_382_389_400_406	4	1	25.000	0.609	-
RF01857_613_1QZW_192_198_207_213	16	4	25.000	1.462	*
RF00177_235_2HHH_1380_1383_1471_1475	179	47	26.257	1.809	+
RF00177_1_1J5E_482_490_519_521	10	3	30.000	0.696	-
RF00177_831_2V48_435_440_463_469	283	86	30.389	1.393	+
RF00177_206_2Z4M_419_423_456_461	1524	497	32.612	1.410	+
RF00177_1124_2VHP_419_427_452_461	6	2	33.333	1.299	*
RF00177_324_1VS5_475_484_513_516	3	1	33.333	1.949	*
RF00177_1_2GYB_490_498_527_529	9	3	33.333	0.877	-
RF00177_1312_2HGR_59_65_89_93	54	18	33.333	0.959	-
RF00177_874_2F4V_1225_1231_1262_1268	408	149	36.520	1.371	+
RF00177_325_1VS5_121_126_141_147	49	18	36.735	0.991	-
RF00177_145_2QBF_1222_1228_1259_1265	414	153	36.957	1.387	+
RF00177_955_2HHH_436_440_463_468	654	243	37.156	1.286	+
RF00167_327_1Y26_26_29_49_56	331	124	37.462	1.242	*
RF00177_1595_1N34_1100_1107_1122_1131	8	3	37.500	1.299	*
RF00001_5_1N8R_75_81_101_106	919	353	38.411	1.202	*
RF00177_32_1VOX_748_752_768_772	499	198	39.679	1.135	*
RF00177_11_2VHP_752_756_772_776	493	196	39.757	1.116	*
RF00001_1334_2GYC_21_28_52_58	1373	547	39.840	1.173	*

Continued on next page

Table A – continued from previous page

p-score > 0.9, 0.25 ≤ GC content ≤ 0.75					
ModelID	# original	# shuffled	FDR (%)	Odds ratio	Stat.
$\hat{F}_{rnaz}$	142 490	66 891	46.9%		
RF00177_35_2B9M_757_761_777_781	529	211	39.887	1.176	*
RF00177_418_1S1H_661_665_676_683	45	18	40.000	1.198	*
RF00015_758_2OZB_28_34_42_45	1964	791	40.275	1.113	*
RF00162_600_2GIS_17_21_32_39	642	259	40.343	1.091	*
RF00001_196_2QBE_69_77_95_103	2380	965	40.546	1.129	*
RF00177_90_1HNW_1281_1285_1308_1312	162	67	41.358	1.234	*
RF00177_1166_2UXD_1224_1231_1262_1269	227	96	42.291	1.102	*
RF00177_231_2GYB_766_770_786_790	527	224	42.505	1.033	*
RF00177_1114_2VHP_1404_1410_1441_1446	885	385	43.503	1.093	*
RF00001_449_3CCQ_32_37_43_48	3147	1371	43.565	1.092	*
RF00177_192_2QBD_657_661_672_679	41	18	43.902	1.276	*
RF00001_1060_3BBO_68_77_96_105	716	318	44.413	1.131	*
RF00177_284_2QBJ_1099_1104_1118_1124	223	102	45.740	1.116	*
RF00017_631_2J37_181_187_212_216	2512	1163	46.298	1.048	*
RF00177_1127_2VHO_1404_1411_1440_1446	219	102	46.575	1.069	*
RF00177_244_3DF3_1278_1282_1305_1309	126	61	48.413	1.044	*
RF00177_1303_2HHH_138_143_158_164	51	25	49.020	1.008	*
RF00017_1608_1L9A_190_194_205_209	196	97	49.490	1.010	*
RF00177_579_2J02_440_446_456_463	690	343	49.710	0.912	-
RF00177_1592_1N36_436_445_457_468	2	1	50.000	0.487	-
RF00059_318_2GDI_55_59_72_76	4865	2558	52.580	0.885	-
RF00177_432_2QP0_215_220_250_257	27	18	66.667	0.821	-
RF00169_236_1CQ5_37_41_52_56	82	57	69.512	0.796	-
RF00177_36_2QB9_862_869_880_885	11	8	72.727	0.975	-
RF00177_334_1HNW_233_238_268_275	21	16	76.190	0.717	-
RF00177_87_2QAN_541_548_855_857	7	6	85.714	0.304	-
RF00177_495_2B64_859_866_877_882	10	9	90.000	0.905	-
RF00177_1167_2UXD_399_407_418_425	1	3	300.000	0.162	-

Table B: False discovery rates (FDR) for individual mRm$_{IL}$ models with mean RMDetect score ≥ $Q_{0.75}$ in windows with p-score > 0.9 and 0.25 ≤ GC content ≤ 0.75 of order R. Status "+" denotes models with adjusted p-value ≤ 0.05 and odds ratio ≥ 1.0 whereas "*" denotes models with odds ratio ≥ 1.0. Models assigned with "-" are neither enriched nor occur at higher rates in the original data. Colored rows have an FDR ≤ $\hat{F}_{rnaz}$ and original window count ≥ 6. The table is sorted in ascending order according to FDR.

p-score > 0.9, 0.25 ≤ GC content ≤ 0.75					
ModelID	# original	# shuffled	FDR (%)	Odds ratio	Stat.
$\hat{F}_{rnaz}$	142 475	66 891	46.9%		
RF00011_1583_1NBS_177_190_209_219	0	0	0.000	0.000	-
RF00177_1133_2VHO_380_389_400_408	1	0	0.000	Inf	*
RF00177_698_2B64_481_491_518_522	1	0	0.000	Inf	*
RF01118_270_2QBC_13_17_35_55	0	0	0.000	0.000	-
RF00177_351_1I95_804_807_829_846	1	0	0.000	Inf	*
RF00177_437_2QBF_488_495_500_509	0	0	0.000	0.000	-
RF00177_551_2UXD_494_500_507_515	0	0	0.000	0.000	-
RF00177_171_1HR0_804_807_829_846	1	0	0.000	Inf	*
RF00177_1470_1XNQ_1103_1111_1121_1128	0	0	0.000	0.000	-
RF00177_1369_2GY9_669_675_686_695	2	0	0.000	Inf	*

Continued on next page

Table B – continued from previous page

**p-score > 0.9, 0.25 ≤ GC content ≤ 0.75**					
ModelID	# original	# shuffled	FDR (%)	Odds ratio	Stat.
$\hat{F}_{rnaz}$	142 475	66 891	46.9%		
RF00177_524_1VOV_400_408_417_424	2	0	0.000	Inf	*
RF00177_942_1N36_137_143_158_165	13	2	15.385	1.686	*
RF00177_857_3BBN_130_136_151_158	13	2	15.385	1.686	*
RF00177_1012_3FIH_120_126_141_148	13	2	15.385	1.349	*
RF00177_243_2QBB_382_389_400_406	4	1	25.000	0.602	-
RF00177_235_2HHH_1380_1383_1471_1475	183	47	25.683	1.866	+
RF01857_613_1QZW_192_198_207_213	15	4	26.667	1.284	*
RF00177_1_1J5E_482_490_519_521	10	3	30.000	0.688	-
RF00177_1312_2HGR_59_65_89_93	58	18	31.034	1.025	*
RF00177_831_2V48_435_440_463_469	277	86	31.047	1.357	+
RF00177_206_2Z4M_419_423_456_461	1553	497	32.003	1.433	+
RF00177_751_1IBL_660_665_676_684	3	1	33.333	2.890	*
RF00177_1124_2VHP_419_427_452_461	6	2	33.333	1.284	*
RF00177_324_1VS5_475_484_513_516	3	1	33.333	1.927	*
RF00177_1_2GYB_490_498_527_529	9	3	33.333	0.963	-
RF00177_325_1VS5_121_126_141_147	51	18	35.294	0.996	-
RF00177_874_2F4V_1225_1231_1262_1268	421	149	35.392	1.414	+
RF00177_145_2QBF_1222_1228_1259_1265	426	153	35.915	1.432	+
RF00177_955_2HHH_436_440_463_468	676	243	35.947	1.334	+
RF00167_327_1Y26_26_29_49_56	338	124	36.686	1.303	+
RF00177_1595_1N34_1100_1107_1122_1131	8	3	37.500	1.445	*
RF00177_32_1VOX_748_752_768_772	517	198	38.298	1.195	*
RF00177_11_2VHP_752_756_772_776	510	196	38.431	1.174	*
RF00001_5_1N8R_75_81_101_106	916	353	38.537	1.195	*
RF00177_90_1HNW_1281_1285_1308_1312	171	67	39.181	1.281	*
RF00001_1334_2GYC_21_28_52_58	1395	547	39.211	1.183	*
RF00177_35_2B9M_757_761_777_781	535	211	39.439	1.186	*
RF00162_600_2GIS_17_21_32_39	652	259	39.724	1.088	*
RF00177_418_1S1H_661_665_676_683	45	18	40.000	1.184	*
RF00015_758_2OZB_28_34_42_45	1964	791	40.275	1.105	*
RF00001_196_2QBE_69_77_95_103	2394	965	40.309	1.131	*
RF00177_1166_2UXD_1224_1231_1262_1269	237	96	40.506	1.162	*
RF00177_231_2GYB_766_770_786_790	545	224	41.101	1.085	*
RF00177_1114_2VHP_1404_1410_1441_1446	888	385	43.356	1.099	*
RF00001_449_3CCQ_32_37_43_48	3147	1371	43.565	1.095	*
RF00177_192_2QBD_657_661_672_679	41	18	43.902	1.261	*
RF00001_1060_3BBO_68_77_96_105	714	318	44.538	1.126	*
RF00177_284_2QBJ_1099_1104_1118_1124	226	102	45.133	1.132	*
RF00177_1127_2VHO_1404_1411_1440_1446	221	102	46.154	1.090	*
RF00017_631_2J37_181_187_212_216	2487	1163	46.763	1.049	*
RF00177_244_3DF3_1278_1282_1305_1309	130	61	46.923	1.080	*
RF00177_1303_2HHH_138_143_158_164	53	25	47.170	1.046	*
RF00017_1608_1L9A_190_194_205_209	197	97	49.239	0.994	-
RF00177_1592_1N36_436_445_457_468	2	1	50.000	0.482	-
RF00177_579_2J02_440_446_456_463	680	343	50.441	0.915	-
RF00059_318_2GDI_55_59_72_76	4893	2558	52.279	0.887	-
RF00177_432_2QP0_215_220_250_257	31	18	58.065	0.887	-
RF00177_334_1HNW_233_238_268_275	25	16	64.000	0.765	-
RF00177_36_2QB9_862_869_880_885	12	8	66.667	0.963	-
RF00169_236_1CQ5_37_41_52_56	79	57	72.152	0.803	-
RF00177_87_2QAN_541_548_855_857	7	6	85.714	0.301	-
RF00177_495_2B64_859_866_877_882	10	9	90.000	0.826	-
RF00177_1167_2UXD_399_407_418_425	1	3	300.000	0.161	-

Table C: False discovery rates (FDR) for individual JAR3D IL models with mean interior edit distance $\leq$ 4 and passed cutoff $\geq$ 50 in windows with p-score $>$ 0.9 and 0.25 $\leq$ GC content $\leq$ 0.75 of order L. Status "+" denotes models with adjusted p-value $\leq$ 0.05 and odds ratio $\geq$ 1.0 whereas "*" denotes models with odds ratio $\geq$ 1.0. Models assigned with "-" are neither enriched nor occur at higher rates in the original data. "Msl" = mean sequence length of module instances. Colored rows have an FDR $\leq \hat{F}_{rnaz}$, original window count $\geq$6, and Msl $\geq$9. The table is sorted in ascending order according to FDR.

**p-score $>$ 0.9, 0.25 $\leq$ GC content $\leq$ 0.75**						
ModelID	# original	# shuffled	FDR (%)	Odds ratio	Stat.	Msl
$\hat{F}_{rnaz}$	142 490	66 891	46.9%			
IL_05513.1	2	0	0.000	Inf	*	12
IL_39324.1	0	0	0.000	0.000	-	20
IL_40527.1	0	0	0.000	0.000	-	16
IL_42891.1	1	0	0.000	Inf	*	16
IL_46435.1	7	0	0.000	Inf	*	13
IL_52610.1	0	0	0.000	0.000	-	37
IL_76095.3	0	0	0.000	0.000	-	20
IL_76263.1	0	0	0.000	0.000	-	19
IL_77076.1	0	0	0.000	0.000	-	18
IL_77296.1	0	0	0.000	0.000	-	15
IL_06847.1	7	0	0.000	Inf	*	13
IL_88367.1	0	0	0.000	0.000	-	15
IL_89028.6	0	0	0.000	0.000	-	16
IL_90057.1	0	0	0.000	0.000	-	25
IL_94744.1	2	0	0.000	Inf	*	17
IL_97842.1	0	0	0.000	0.000	-	16
IL_98655.1	0	0	0.000	0.000	-	16
IL_02359.3	0	0	0.000	0.000	-	17.6
IL_16330.1	0	0	0.000	0.000	-	28
IL_21421.1	0	0	0.000	0.000	-	20
IL_21495.1	1	0	0.000	0.000	-	15
IL_23414.1	0	0	0.000	0.000	-	21
IL_25082.1	3	0	0.000	Inf	*	18
IL_25230.3	0	0	0.000	0.000	-	18
IL_27668.1	2	0	0.000	Inf	*	12
IL_28572.2	7	0	0.000	Inf	*	16
IL_33964.1	0	0	0.000	0.000	-	23
IL_80505.1	11	1	9.091	4.386	*	11
IL_34628.2	79	11	13.924	2.491	+	13
IL_39526.4	7	1	14.286	2.924	*	9
IL_98591.3	6	1	16.667	2.436	*	16
IL_63133.1	53	9	16.981	2.925	+	6
IL_23448.1	92	16	17.391	3.225	+	6

Continued on next page

Table C – continued from previous page

p-score > 0.9, 0.25 ≤ GC content ≤ 0.75						
ModelID	# original	# shuffled	FDR (%)	Odds ratio	Stat.	Msl
$\hat{F}_{rnaz}$	142 490	66 891	46.9%			
IL_61730.1	28	5	17.857	2.339	*	11
IL_21639.1	51	10	19.608	2.329	*	9
IL_77014.1	5	1	20.000	Inf	*	11
IL_57364.1	29	6	20.690	2.534	*	8
IL_86357.3	82	17	20.732	2.315	+	6
IL_41791.1	92	20	21.739	1.488	*	12
IL_34363.2	23	5	21.739	2.437	*	14
IL_96206.3	315	72	22.857	2.192	+	6
IL_73000.2	274	63	22.993	2.223	+	6.57
IL_58291.4	134	31	23.134	2.360	+	6
IL_28644.1	157	38	24.204	2.128	+	6
IL_06177.1	41	10	24.390	1.852	*	6
IL_69799.1	123	30	24.390	2.184	+	6
IL_54450.1	45	11	24.444	1.728	*	8
IL_73276.5	217	54	24.885	1.951	+	8.18
IL_71942.1	12	3	25.000	1.462	*	10
IL_75415.1	8	2	25.000	0.975	-	10
IL_85647.3	16	4	25.000	1.706	*	15.06
IL_91089.1	48	12	25.000	2.715	+	8
IL_99397.1	20	5	25.000	1.657	*	9
IL_31066.3	44	11	25.000	1.949	*	7
IL_43877.1	91	23	25.275	1.759	+	7.5
IL_70173.1	185	48	25.946	1.895	+	7.5
IL_37406.1	99	26	26.263	1.795	+	8.5
IL_39980.1	125	33	26.400	1.842	+	8
IL_98421.4	125	33	26.400	1.698	+	7
IL_37325.1	53	14	26.415	2.031	*	7
IL_87065.1	147	39	26.531	1.681	+	7
IL_92027.3	165	44	26.667	1.924	+	6.24
IL_92027.3	165	44	26.667	1.924	+	6.24
IL_67887.1	231	62	26.840	1.800	+	6.5
IL_28947.2	346	93	26.879	1.869	+	5.45
IL_40845.1	212	57	26.887	1.727	+	8
IL_06808.1	103	28	27.184	1.599	*	9
IL_41153.1	33	9	27.273	1.516	*	8
IL_58586.2	330	90	27.273	1.787	+	7
IL_50911.1	40	11	27.500	1.841	*	8
IL_44540.4	224	62	27.679	1.756	+	5.3
IL_39199.4	289	80	27.682	1.775	+	5.28
IL_97217.11	371	103	27.763	1.750	+	5.14
IL_43946.1	18	5	27.778	1.584	*	12
IL_98566.1	96	27	28.125	1.462	*	9
IL_50521.1	63	18	28.571	1.491	*	15
IL_11751.1	42	12	28.571	1.418	*	7

Continued on next page

Table C – continued from previous page

**p-score > 0.9, 0.25 ≤ GC content ≤ 0.75**						
ModelID	# original	# shuffled	FDR (%)	Odds ratio	Stat.	Msl
$\hat{F}_{rnaz}$	142 490	66 891	46.9%			
IL_25300.3	309	89	28.803	1.674	+	5.29
IL_47875.1	548	160	29.197	1.730	+	7.67
IL_92109.3	567	166	29.277	1.634	+	6.07
IL_46648.6	235	69	29.362	1.652	+	5.62
IL_94430.5	383	113	29.504	1.621	+	5.94
IL_15205.1	151	45	29.801	1.414	*	13
IL_56465.4	512	153	29.883	1.561	+	5.74
IL_01080.1	226	68	30.088	1.650	+	8
IL_31039.1	63	19	30.159	1.605	*	7
IL_56513.1	112	34	30.357	1.386	*	9
IL_31663.1	112	34	30.357	1.415	*	7.5
IL_20775.1	69	21	30.435	1.462	*	8
IL_09491.1	36	11	30.556	1.462	*	8
IL_82444.1	191	59	30.890	1.437	*	6.59
IL_41139.1	64	20	31.250	1.411	*	8
IL_68767.1	105	33	31.429	1.654	*	6
IL_18675.1	105	33	31.429	1.625	*	7
IL_13069.3	91	29	31.868	1.294	*	8
IL_86059.1	116	37	31.897	1.536	*	7
IL_17066.1	72	23	31.944	1.420	*	6
IL_63253.1	97	31	31.959	1.657	*	7
IL_21254.1	50	16	32.000	1.218	*	14
IL_17212.2	230	74	32.174	1.375	*	6.73
IL_21333.2	299	97	32.441	1.507	+	9.5
IL_88119.1	199	65	32.663	1.463	+	8
IL_02809.3	281	92	32.740	1.590	+	9.11
IL_80348.3	191	63	32.984	1.505	+	6.33
IL_31006.1	218	72	33.028	1.325	*	12
IL_12147.1	220	73	33.182	1.448	+	6.5
IL_37347.1	18	6	33.333	1.364	*	16
IL_47732.1	21	7	33.333	1.183	*	9
IL_86981.1	3	1	33.333	0.975	-	15
IL_91379.1	9	3	33.333	1.706	*	12
IL_35043.1	6	2	33.333	1.462	*	11
IL_47174.11	295	99	33.559	1.380	+	6.2
IL_16166.4	140	47	33.571	1.419	*	5
IL_13777.1	118	40	33.898	1.462	*	5
IL_95150.6	76	26	34.211	1.584	*	8.33
IL_90133.3	166	57	34.337	1.334	*	8
IL_13959.4	183	63	34.426	1.367	*	8.23
IL_42251.1	209	72	34.450	1.397	*	7
IL_25271.2	26	9	34.615	1.868	*	15
IL_37104.3	219	76	34.703	1.305	*	7.36
IL_71685.1	92	32	34.783	1.186	*	14

Continued on next page

p-score > 0.9, 0.25 ≤ GC content ≤ 0.75						
ModelID	# original	# shuffled	FDR (%)	Odds ratio	Stat.	Msl
$\hat{F}_{rnaz}$	142 490	66 891	46.9%			
IL_57785.5	54	19	35.185	1.319	*	9
IL_23262.4	535	189	35.327	1.372	+	11
IL_69106.1	205	73	35.610	1.279	*	8.5
IL_90459.3	537	192	35.754	1.283	+	7.56
IL_80494.2	591	213	36.041	1.246	*	12.67
IL_46721.1	102	37	36.275	1.584	*	11
IL_22909.1	118	43	36.441	1.283	*	9
IL_79083.3	798	291	36.466	1.286	+	11
IL_30381.1	71	26	36.620	1.377	*	8.5
IL_88865.1	30	11	36.667	1.408	*	9
IL_11778.1	38	14	36.842	1.237	*	8
IL_63952.1	617	228	36.953	1.264	+	13
IL_55938.4	339	126	37.168	1.256	*	7.56
IL_43316.1	1022	380	37.182	1.229	*	11
IL_87507.1	258	96	37.209	1.124	*	9.5
IL_39355.1	400	150	37.500	1.193	*	9
IL_08926.3	114	43	37.719	1.255	*	5.5
IL_47758.2	127	48	37.795	1.192	*	8.5
IL_55649.1	452	171	37.832	1.208	*	9
IL_97296.1	234	89	38.034	1.183	*	8.5
IL_83920.1	68	26	38.235	1.170	*	7
IL_70401.1	13	5	38.462	1.218	*	11
IL_05684.1	262	101	38.550	1.235	*	9
IL_78513.1	31	12	38.710	0.930	-	10
IL_87394.1	31	12	38.710	0.934	-	10
IL_87904.5	1389	538	38.733	1.219	*	10
IL_43124.2	190	74	38.947	1.137	*	7.09
IL_83856.1	646	252	39.009	1.267	+	12
IL_41766.6	379	148	39.050	1.208	*	8.26
IL_64589.1	64	25	39.062	1.323	*	8
IL_12211.1	151	59	39.073	1.275	*	13.5
IL_57977.1	87	34	39.080	1.088	*	11
IL_06421.1	115	45	39.130	1.063	*	8
IL_65553.8	130	51	39.231	1.257	*	13.3
IL_24546.4	175	69	39.429	1.223	*	10
IL_74876.2	1017	401	39.430	1.220	*	10.67
IL_06471.1	595	235	39.496	1.158	*	10
IL_26971.1	1027	409	39.825	1.159	*	11
IL_06211.3	158	63	39.873	1.237	*	9.33
IL_75328.1	25	10	40.000	1.532	*	14
IL_00998.1	110	44	40.000	1.238	*	8
IL_82650.1	15	6	40.000	0.812	-	13
IL_15840.2	874	350	40.046	1.138	*	11
IL_77263.2	354	142	40.113	1.095	*	11

Continued on next page

Table C – continued from previous page

ModelID	# original	# shuffled	FDR (%)	Odds ratio	Stat.	Msl
**p-score > 0.9, 0.25 ≤ GC content ≤ 0.75**						
$\hat{F}_{rnaz}$	142 490	66 891	46.9%			
IL_24982.5	1614	650	40.273	1.165	*	10
IL_37197.1	804	324	40.299	1.168	*	11
IL_22732.1	209	85	40.670	1.133	*	9
IL_85510.1	853	348	40.797	1.107	*	11
IL_97191.1	17	7	41.176	1.137	*	13
IL_97509.1	889	369	41.507	1.102	*	10.5
IL_80714.1	563	234	41.563	1.132	*	12
IL_47444.3	953	399	41.868	1.089	*	12
IL_70237.3	1093	459	41.995	1.122	*	10
IL_07300.2	150	63	42.000	1.110	*	9.33
IL_80093.1	740	311	42.027	1.125	*	11
IL_31224.1	364	153	42.033	1.039	*	10
IL_53635.3	121	51	42.149	1.200	*	13
IL_45794.1	52	22	42.308	0.950	-	14
IL_93568.2	234	99	42.308	1.098	*	12.5
IL_93424.4	743	315	42.396	1.076	*	10.19
IL_39585.1	1205	511	42.407	1.115	*	12
IL_87548.1	510	217	42.549	1.100	*	11.5
IL_05723.1	568	243	42.782	1.141	*	10
IL_11302.1	1068	457	42.790	1.025	*	10
IL_09587.1	3315	1419	42.805	1.071	*	11.5
IL_45262.4	175	75	42.857	1.057	*	9.25
IL_52509.1	630	270	42.857	1.016	*	10
IL_70299.1	95	41	43.158	1.147	*	14
IL_46306.1	1422	616	43.319	1.042	*	10
IL_21077.1	203	88	43.350	1.001	*	10
IL_25380.1	191	83	43.455	1.078	*	8.33
IL_09333.1	377	164	43.501	1.070	*	13
IL_79955.2	429	187	43.590	1.097	*	12.67
IL_69536.1	1303	569	43.668	1.090	*	10
IL_31555.5	746	328	43.968	1.064	*	9.23
IL_86336.1	352	156	44.318	0.997	-	10
IL_06468.1	313	140	44.728	1.041	*	11
IL_46034.3	96	43	44.792	0.962	-	9
IL_09530.1	790	354	44.810	1.038	*	11.5
IL_82563.1	60	27	45.000	0.975	-	10
IL_54966.1	332	150	45.181	1.012	*	9
IL_23639.1	1379	626	45.395	1.029	*	10
IL_52173.1	198	90	45.455	1.055	*	9
IL_17603.1	409	187	45.721	1.147	*	12
IL_58454.1	694	318	45.821	1.044	*	10
IL_54470.1	438	201	45.890	0.971	-	9
IL_38807.3	76	35	46.053	1.162	*	12.75
IL_06180.1	1092	507	46.429	1.001	*	10.67

Continued on next page

Table C – continued from previous page

**p-score > 0.9, 0.25 ≤ GC content ≤ 0.75**						
ModelID	# original	# shuffled	FDR (%)	Odds ratio	Stat.	Msl
$\hat{F}_{rnaz}$	142 490	66 891	46.9%			
IL_95652.3	43	20	46.512	1.007	*	14
IL_82188.2	273	127	46.520	0.952	-	14
IL_87523.1	170	80	47.059	1.006	*	11
IL_16415.2	17	8	47.059	0.792	-	15
IL_03110.1	448	211	47.098	0.983	-	13
IL_52958.1	3115	1470	47.191	0.993	-	10
IL_30067.1	332	157	47.289	1.045	*	10
IL_06306.1	38	18	47.368	1.312	*	14
IL_26868.1	472	224	47.458	0.969	-	11
IL_59529.1	578	275	47.578	1.013	*	11
IL_91044.1	382	182	47.644	0.978	-	11
IL_46301.1	291	139	47.766	0.995	-	9.43
IL_78744.1	23	11	47.826	1.323	*	12
IL_24293.1	81	39	48.148	0.893	-	8.5
IL_53988.1	120	58	48.333	0.897	-	13
IL_49493.4	132	64	48.485	0.938	-	13
IL_98924.1	500	243	48.600	0.886	-	10
IL_96446.1	146	71	48.630	0.871	-	11
IL_97057.3	820	399	48.659	1.000	*	11.83
IL_81398.1	3817	1861	48.756	0.968	-	10
IL_12507.1	92	45	48.913	0.938	-	13
IL_03282.1	143	70	48.951	0.921	-	10
IL_28003.1	65	32	49.231	1.044	*	8.67
IL_62499.7	560	278	49.643	0.898	-	11
IL_92321.4	559	278	49.732	0.930	-	12.14
IL_49527.1	894	445	49.776	0.969	-	10
IL_65137.1	108	54	50.000	0.932	-	13
IL_66744.1	94	47	50.000	0.868	-	14
IL_80652.1	18	9	50.000	1.044	*	14
IL_82601.1	2	1	50.000	Inf	*	8
IL_94403.5	2	1	50.000	Inf	*	12.75
IL_02835.1	4	2	50.000	1.949	*	13
IL_27640.1	2	1	50.000	0.975	-	15
IL_33842.1	26	13	50.000	0.934	-	9
IL_46990.1	2086	1051	50.384	0.944	-	12.5
IL_30840.2	2798	1411	50.429	0.928	-	11.33
IL_37053.1	299	151	50.502	0.954	-	13.67
IL_85805.1	1094	555	50.731	0.946	-	9
IL_53323.1	1176	597	50.765	0.893	-	10
IL_40892.1	121	62	51.240	1.052	*	11
IL_72158.3	161	83	51.553	0.892	-	13
IL_83250.1	147	76	51.701	1.002	*	11
IL_48918.3	86	45	52.326	0.909	-	13
IL_25307.1	70	37	52.857	0.821	-	9

Continued on next page

Table C – continued from previous page

**p-score > 0.9, 0.25 ≤ GC content ≤ 0.75**						
ModelID	# original	# shuffled	FDR (%)	Odds ratio	Stat.	Msl
$\hat{F}_{rnaz}$	142 490	66 891	46.9%			
IL_09882.1	122	65	53.279	1.018	*	12
IL_91078.1	15	8	53.333	0.975	-	14
IL_25181.1	1247	673	53.970	0.902	-	13
IL_49976.1	1247	693	55.573	0.846	-	13
IL_31754.1	7	4	57.143	0.731	-	13
IL_97833.1	33	19	57.576	1.012	*	14
IL_47687.1	291	168	57.732	0.842	-	14
IL_59934.1	94	55	58.511	0.879	-	13
IL_89794.1	29	17	58.621	0.870	-	14
IL_60649.1	51	30	58.824	0.758	-	13
IL_92114.3	74	44	59.459	0.837	-	9
IL_31707.1	13	8	61.538	0.731	-	11
IL_52940.1	270	167	61.852	0.853	-	15.5
IL_44067.4	16	10	62.500	0.731	-	14
IL_54954.1	3	2	66.667	1.462	*	17
IL_76486.1	3	2	66.667	0.731	-	15
IL_94973.1	17	12	70.588	0.532	-	14
IL_68827.1	35	27	77.143	0.508	-	11
IL_17682.1	1	1	100.000	0.000	-	17
IL_05221.1	3	3	100.000	0.325	-	17
IL_90735.1	20	21	105.000	0.585	-	17
IL_27243.1	2	3	150.000	0.244	-	15
IL_57285.1	0	1	Inf	0.000	-	13
IL_91904.1	0	1	Inf	0.000	-	17

Table D: False discovery rates (FDR) for individual JAR3D IL models with mean interior edit distance ≤ 4 and passed cutoff ≥ 50 in windows with p-score > 0.9 and 0.25 ≤ GC content ≤ 0.75 of order R. Status "+" denotes models with adjusted p-value ≤ 0.05 and odds ratio ≥ 1.0 whereas "*" denotes models with odds ratio ≥1.0. Models assigned with "-" are neither enriched nor occur at higher rates in the original data. "Msl" = mean sequence length of module instances. Colored rows have an FDR ≤ $\hat{F}_{rnaz}$ and original window count ≥6, and Msl ≥9. The table is sorted in ascending order according to FDR.

**p-score > 0.9, 0.25 ≤ GC content ≤ 0.75**						
ModelID	# original	# shuffled	FDR (%)	Odds ratio	Stat.	Msl
$\hat{F}_{rnaz}$	142 475	66 891	46.9%			
IL_05513.1	2	0	0.000	Inf	*	12
IL_39324.1	0	0	0.000	0.000	-	20
IL_40527.1	0	0	0.000	0.000	-	16

Continued on next page

Table D – continued from previous page

**p-score > 0.9, 0.25 ≤ GC content ≤ 0.75**						
ModelID	# original	# shuffled	FDR (%)	Odds ratio	Stat.	Msl
$\hat{F}_{rnaz}$	142 475	66 891	46.9%			
IL_42891.1	1	0	0.000	Inf	*	16
IL_46435.1	7	0	0.000	Inf	*	13
IL_52610.1	0	0	0.000	0.000	-	37
IL_76095.3	0	0	0.000	0.000	-	20
IL_76263.1	0	0	0.000	0.000	-	19
IL_77076.1	0	0	0.000	0.000	-	18
IL_77296.1	0	0	0.000	0.000	-	15
IL_06847.1	7	0	0.000	Inf	*	13
IL_88367.1	0	0	0.000	0.000	-	15
IL_89028.6	0	0	0.000	0.000	-	16
IL_90057.1	0	0	0.000	0.000	-	25
IL_94744.1	2	0	0.000	Inf	*	17
IL_97842.1	0	0	0.000	0.000	-	16
IL_98655.1	0	0	0.000	0.000	-	16
IL_02359.3	0	0	0.000	0.000	-	17.6
IL_16330.1	0	0	0.000	0.000	-	28
IL_21421.1	0	0	0.000	0.000	-	20
IL_21495.1	1	0	0.000	0.000	-	15
IL_23414.1	0	0	0.000	0.000	-	21
IL_25082.1	2	0	0.000	Inf	*	18
IL_25230.3	0	0	0.000	0.000	-	18
IL_27668.1	2	0	0.000	Inf	*	12
IL_28572.2	7	0	0.000	Inf	*	16
IL_33964.1	0	0	0.000	0.000	-	23
IL_80505.1	11	1	9.091	4.337	*	11
IL_34628.2	83	11	13.253	2.785	+	13
IL_39526.4	7	1	14.286	2.891	*	9
IL_63133.1	58	9	15.517	3.193	+	6
IL_98591.3	6	1	16.667	2.409	*	16
IL_23448.1	95	16	16.842	3.301	+	6
IL_61730.1	28	5	17.857	2.313	*	11
IL_41791.1	104	20	19.231	1.776	*	12
IL_21639.1	52	10	19.231	2.356	+	9
IL_86357.3	85	17	20.000	2.380	+	6
IL_57364.1	28	6	21.429	2.506	*	8
IL_96206.3	326	72	22.086	2.260	+	6
IL_73000.2	282	63	22.340	2.269	+	6.57
IL_28644.1	169	38	22.485	2.280	+	6
IL_58291.4	137	31	22.628	2.411	+	6
IL_34363.2	22	5	22.727	2.289	*	14
IL_54450.1	48	11	22.917	1.796	*	8
IL_06177.1	43	10	23.256	1.928	*	6
IL_69799.1	128	30	23.438	2.276	+	6
IL_73276.5	223	54	24.215	1.973	+	8.18

Continued on next page

Table D – continued from previous page

p-score > 0.9, 0.25 ≤ GC content ≤ 0.75						
ModelID	# original	# shuffled	FDR (%)	Odds ratio	Stat.	Msl
$\hat{F}_{rnaz}$	142 475	66 891	46.9%			
IL_43877.1	94	23	24.468	1.802	+	7.5
IL_70173.1	192	48	25.000	1.951	+	7.5
IL_71942.1	12	3	25.000	1.285	*	10
IL_75415.1	8	2	25.000	0.964	-	10
IL_77014.1	4	1	25.000	Inf	*	11
IL_85647.3	16	4	25.000	1.687	*	15.06
IL_91089.1	48	12	25.000	2.685	+	8
IL_31066.3	44	11	25.000	1.928	*	7
IL_92027.3	174	44	25.287	2.007	+	6.24
IL_92027.3	174	44	25.287	2.007	+	6.24
IL_87065.1	152	39	25.658	1.725	+	7
IL_98421.4	128	33	25.781	1.712	+	7
IL_28947.2	358	93	25.978	1.913	+	5.45
IL_67887.1	238	62	26.050	1.850	+	6.5
IL_37406.1	99	26	26.263	1.797	+	8.5
IL_58586.2	342	90	26.316	1.855	+	7
IL_99397.1	19	5	26.316	1.542	*	9
IL_37325.1	53	14	26.415	2.008	*	7
IL_98566.1	102	27	26.471	1.581	*	9
IL_44540.4	234	62	26.496	1.815	+	5.3
IL_06808.1	105	28	26.667	1.619	*	9
IL_97217.11	386	103	26.684	1.809	+	5.14
IL_40845.1	212	57	26.887	1.728	+	8
IL_39199.4	297	80	26.936	1.802	+	5.28
IL_39980.1	122	33	27.049	1.785	+	8
IL_41153.1	33	9	27.273	1.499	*	8
IL_91379.1	11	3	27.273	2.168	*	12
IL_11751.1	44	12	27.273	1.446	*	7
IL_43946.1	18	5	27.778	1.566	*	12
IL_25300.3	320	89	27.812	1.721	+	5.29
IL_46648.6	248	69	27.823	1.727	+	5.62
IL_50911.1	39	11	28.205	1.767	*	8
IL_47875.1	567	160	28.219	1.770	+	7.67
IL_92109.3	587	166	28.279	1.687	+	6.07
IL_31039.1	67	19	28.358	1.673	*	7
IL_94430.5	396	113	28.535	1.670	+	5.94
IL_56465.4	526	153	29.087	1.583	+	5.74
IL_50521.1	61	18	29.508	1.417	*	15
IL_31663.1	115	34	29.565	1.461	*	7.5
IL_01080.1	229	68	29.694	1.672	+	8
IL_15205.1	151	45	29.801	1.410	*	13
IL_56513.1	114	34	29.825	1.416	*	9
IL_82444.1	197	59	29.949	1.455	+	6.59
IL_20775.1	70	21	30.000	1.492	*	8

Continued on next page

Table D – continued from previous page

p-score > 0.9, 0.25 ≤ GC content ≤ 0.75						
ModelID	# original	# shuffled	FDR (%)	Odds ratio	Stat.	Msl
$\hat{F}_{rnaz}$	142 475	66 891	46.9%			
IL_63253.1	103	31	30.097	1.755	+	7
IL_13069.3	96	29	30.208	1.346	*	8
IL_68767.1	109	33	30.275	1.722	+	6
IL_09491.1	36	11	30.556	1.494	*	8
IL_17212.2	241	74	30.705	1.420	+	6.73
IL_41139.1	65	20	30.769	1.420	*	8
IL_25271.2	29	9	31.034	2.088	*	15
IL_86059.1	119	37	31.092	1.578	+	7
IL_18675.1	106	33	31.132	1.625	*	7
IL_80348.3	202	63	31.188	1.573	+	6.33
IL_21254.1	51	16	31.373	1.274	*	14
IL_88119.1	205	65	31.707	1.487	+	8
IL_47732.1	22	7	31.818	1.239	*	9
IL_17066.1	72	23	31.944	1.404	*	6
IL_31006.1	225	72	32.000	1.394	+	12
IL_02809.3	286	92	32.168	1.614	+	9.11
IL_21333.2	301	97	32.226	1.522	+	9.5
IL_13777.1	124	40	32.258	1.510	*	5
IL_16166.4	145	47	32.414	1.457	*	5
IL_47174.11	305	99	32.459	1.421	+	6.2
IL_95150.6	80	26	32.500	1.663	*	8.33
IL_12147.1	223	73	32.735	1.461	+	6.5
IL_37104.3	230	76	33.043	1.368	*	7.36
IL_42251.1	217	72	33.180	1.446	+	7
IL_37347.1	18	6	33.333	1.349	*	16
IL_57785.5	57	19	33.333	1.389	*	9
IL_86981.1	3	1	33.333	0.964	-	15
IL_35043.1	6	2	33.333	1.446	*	11
IL_23262.4	563	189	33.570	1.451	+	11
IL_71685.1	95	32	33.684	1.205	*	14
IL_90133.3	168	57	33.929	1.328	*	8
IL_69106.1	215	73	33.953	1.348	*	8.5
IL_13959.4	185	63	34.054	1.377	*	8.23
IL_46721.1	108	37	34.259	1.670	+	11
IL_80494.2	613	213	34.747	1.301	+	12.67
IL_90459.3	551	192	34.846	1.310	+	7.56
IL_79083.3	831	291	35.018	1.337	+	11
IL_30381.1	73	26	35.616	1.404	*	8.5
IL_39355.1	420	150	35.714	1.259	*	9
IL_11778.1	39	14	35.897	1.260	*	8
IL_63952.1	633	228	36.019	1.306	+	13
IL_43316.1	1053	380	36.087	1.267	+	11
IL_55938.4	347	126	36.311	1.290	+	7.56
IL_87394.1	33	12	36.364	1.004	*	10

Continued on next page

Table D – continued from previous page

**p-score > 0.9, 0.25 ≤ GC content ≤ 0.75**						
ModelID	# original	# shuffled	FDR (%)	Odds ratio	Stat.	Msl
$\hat{F}_{rnaz}$	142 475	66 891	46.9%			
IL_22909.1	118	43	36.441	1.281	*	9
IL_87507.1	263	96	36.502	1.156	*	9.5
IL_06421.1	123	45	36.585	1.139	*	8
IL_55649.1	467	171	36.617	1.251	+	9
IL_47758.2	131	48	36.641	1.230	*	8.5
IL_97296.1	242	89	36.777	1.223	*	8.5
IL_08926.3	116	43	37.069	1.253	*	5.5
IL_87904.5	1444	538	37.258	1.263	+	10
IL_43124.2	198	74	37.374	1.165	*	7.09
IL_05684.1	269	101	37.546	1.265	*	9
IL_83856.1	669	252	37.668	1.313	+	12
IL_57977.1	90	34	37.778	1.092	*	11
IL_74876.2	1061	401	37.795	1.274	+	10.67
IL_64589.1	66	25	37.879	1.354	*	8
IL_88865.1	29	11	37.931	1.339	*	9
IL_65553.8	134	51	38.060	1.294	*	13.3
IL_12211.1	155	59	38.065	1.323	*	13.5
IL_83920.1	68	26	38.235	1.157	*	7
IL_41766.6	387	148	38.243	1.223	*	8.26
IL_00998.1	115	44	38.261	1.303	*	8
IL_70401.1	13	5	38.462	1.205	*	11
IL_06471.1	611	235	38.462	1.194	*	10
IL_24982.5	1673	650	38.852	1.207	*	10
IL_26971.1	1049	409	38.990	1.193	*	11
IL_15840.2	889	350	39.370	1.158	*	11
IL_77263.2	360	142	39.444	1.119	*	11
IL_93568.2	250	99	39.600	1.173	*	12.5
IL_06211.3	159	63	39.623	1.233	*	9.33
IL_37197.1	817	324	39.657	1.179	*	11
IL_80093.1	781	311	39.821	1.193	*	11
IL_85510.1	873	348	39.863	1.140	*	11
IL_82650.1	15	6	40.000	0.803	-	13
IL_97509.1	921	369	40.065	1.137	*	10.5
IL_24546.4	172	69	40.116	1.182	*	10
IL_39585.1	1269	511	40.268	1.177	*	12
IL_93424.4	781	315	40.333	1.135	*	10.19
IL_22732.1	210	85	40.476	1.133	*	9
IL_80714.1	578	234	40.484	1.172	*	12
IL_70237.3	1128	459	40.691	1.164	*	10
IL_45794.1	54	22	40.741	1.012	*	14
IL_05723.1	594	243	40.909	1.187	*	10
IL_97191.1	17	7	41.176	1.124	*	13
IL_78513.1	29	12	41.379	0.876	-	10
IL_53635.3	123	51	41.463	1.248	*	13

Continued on next page

Table D – continued from previous page

ModelID	# original	# shuffled	FDR (%)	Odds ratio	Stat.	Msl
**p-score > 0.9, 0.25 ≤ GC content ≤ 0.75**						
$\hat{F}_{rnaz}$	142 475	66 891	46.9%			
IL_87548.1	523	217	41.491	1.140	*	11.5
IL_25380.1	200	83	41.500	1.134	*	8.33
IL_11302.1	1098	457	41.621	1.062	*	10
IL_69536.1	1367	569	41.624	1.149	*	10
IL_75328.1	24	10	41.667	1.446	*	14
IL_47444.3	956	399	41.736	1.087	*	12
IL_79955.2	448	187	41.741	1.149	*	12.67
IL_70299.1	98	41	41.837	1.176	*	14
IL_31224.1	365	153	41.918	1.039	*	10
IL_09587.1	3384	1419	41.933	1.098	*	11.5
IL_46306.1	1464	616	42.077	1.079	*	10
IL_16415.2	19	8	42.105	0.903	-	15
IL_45262.4	178	75	42.135	1.090	*	9.25
IL_09333.1	388	164	42.268	1.107	*	13
IL_07300.2	149	63	42.282	1.098	*	9.33
IL_21077.1	208	88	42.308	1.022	*	10
IL_52509.1	638	270	42.320	1.027	*	10
IL_31555.5	763	328	42.988	1.085	*	9.23
IL_86336.1	361	156	43.213	1.014	*	10
IL_23639.1	1448	626	43.232	1.078	*	10
IL_06468.1	322	140	43.478	1.078	*	11
IL_09530.1	812	354	43.596	1.065	*	11.5
IL_58454.1	728	318	43.681	1.093	*	10
IL_17603.1	428	187	43.692	1.195	*	12
IL_46034.3	98	43	43.878	0.988	-	9
IL_54966.1	341	150	43.988	1.037	*	9
IL_91044.1	412	182	44.175	1.061	*	11
IL_06180.1	1141	507	44.435	1.049	*	10.67
IL_59529.1	614	275	44.788	1.086	*	11
IL_82563.1	60	27	45.000	0.964	-	10
IL_52958.1	3265	1470	45.023	1.049	*	10
IL_52173.1	199	90	45.226	1.043	*	9
IL_24293.1	86	39	45.349	0.964	-	8.5
IL_54470.1	443	201	45.372	0.972	-	9
IL_49493.4	139	64	46.043	0.991	-	13
IL_38807.3	76	35	46.053	1.131	*	12.75
IL_30067.1	340	157	46.176	1.072	*	10
IL_46301.1	300	139	46.333	1.023	*	9.43
IL_03110.1	455	211	46.374	1.008	*	13
IL_26868.1	483	224	46.377	1.004	*	11
IL_33842.1	28	13	46.429	1.004	*	9
IL_97057.3	858	399	46.503	1.051	*	11.83
IL_81398.1	3957	1861	47.031	1.010	*	10
IL_82188.2	270	127	47.037	0.950	-	14

Continued on next page

Table D – continued from previous page

p-score > 0.9, 0.25 ≤ GC content ≤ 0.75						
ModelID	# original	# shuffled	FDR (%)	Odds ratio	Stat.	Msl
$\hat{F}_{rnaz}$	142 475	66 891	46.9%			
IL_53988.1	123	58	47.154	0.944	-	13
IL_98924.1	515	243	47.184	0.923	-	10
IL_06306.1	38	18	47.368	1.297	*	14
IL_92321.4	584	278	47.603	0.972	-	12.14
IL_95652.3	42	20	47.619	0.964	-	14
IL_78744.1	23	11	47.826	1.377	*	12
IL_87523.1	166	80	48.193	0.979	-	11
IL_03282.1	145	70	48.276	0.937	-	10
IL_96446.1	147	71	48.299	0.877	-	11
IL_12507.1	93	45	48.387	0.940	-	13
IL_28003.1	66	32	48.485	1.067	*	8.67
IL_62499.7	573	278	48.517	0.922	-	11
IL_65137.1	111	54	48.649	0.953	-	13
IL_53323.1	1227	597	48.655	0.934	-	10
IL_85805.1	1139	555	48.727	0.988	-	9
IL_49527.1	912	445	48.794	0.992	-	10
IL_46990.1	2143	1051	49.043	0.974	-	12.5
IL_30840.2	2864	1411	49.267	0.959	-	11.33
IL_48918.3	91	45	49.451	0.951	-	13
IL_37053.1	304	151	49.671	0.972	-	13.67
IL_72158.3	167	83	49.701	0.934	-	13
IL_80652.1	18	9	50.000	1.033	*	14
IL_82601.1	2	1	50.000	Inf	*	8
IL_94403.5	2	1	50.000	Inf	*	12.75
IL_09882.1	130	65	50.000	1.082	*	12
IL_02835.1	4	2	50.000	1.927	*	13
IL_27640.1	2	1	50.000	0.964	-	15
IL_66744.1	92	47	51.087	0.846	-	14
IL_83250.1	148	76	51.351	1.018	*	11
IL_25307.1	70	37	52.857	0.812	-	9
IL_40892.1	117	62	52.991	1.029	*	11
IL_25181.1	1266	673	53.160	0.916	-	13
IL_91078.1	15	8	53.333	0.964	-	14
IL_49976.1	1271	693	54.524	0.857	-	13
IL_92114.3	79	44	55.696	0.902	-	9
IL_97833.1	34	19	55.882	1.038	*	14
IL_60649.1	53	30	56.604	0.803	-	13
IL_31754.1	7	4	57.143	0.723	-	13
IL_59934.1	96	55	57.292	0.880	-	13
IL_47687.1	293	168	57.338	0.855	-	14
IL_89794.1	28	17	60.714	0.860	-	14
IL_52940.1	272	167	61.397	0.855	-	15.5
IL_44067.4	16	10	62.500	0.723	-	14
IL_54954.1	3	2	66.667	1.446	*	17

Continued on next page

Table D – continued from previous page

**p-score > 0.9, 0.25 ≤ GC content ≤ 0.75**						
ModelID	# original	# shuffled	FDR (%)	Odds ratio	Stat.	Msl
$\hat{F}_{rnaz}$	142 475	66 891	46.9%			
IL_76486.1	3	2	66.667	0.723	-	15
IL_94973.1	18	12	66.667	0.569	-	14
IL_31707.1	12	8	66.667	0.642	-	11
IL_68827.1	38	27	71.053	0.545	-	11
IL_05221.1	4	3	75.000	0.482	-	17
IL_17682.1	1	1	100.000	0.000	-	17
IL_90735.1	20	21	105.000	0.578	-	17
IL_27243.1	2	3	150.000	0.241	-	15
IL_57285.1	0	1	Inf	0.000	-	13
IL_91904.1	0	1	Inf	0.000	-	17

Table E: False discovery rates (FDR) for individual JAR3D HL models with mean interior edit distance ≤ 4 and passed cutoff ≥ 50 in windows with p-score > 0.9 and $0.25 \leq$ GC content ≤ 0.75 of order L. Status "+" denotes models with adjusted p-value ≤ 0.05 and odds ratio ≥ 1.0 whereas "*" denotes models with odds ratio ≥ 1.0. Models assigned with "-" are neither enriched nor occur at higher rates in the original data. Colored rows have an FDR $\leq \hat{F}_{rnaz}$ and original window count ≥ 6. The table is sorted in ascending order according to FDR.

**p-score > 0.9, 0.25 ≤ GC content ≤ 0.75**					
ModelID	# original	# shuffled	FDR (%)	Odds ratio	Stat.
$\hat{F}_{rnaz}$	142 490	66 891	46.9%		
HL_45358.1	0	0	0.000	0.000	-
HL_10116.1	0	0	0.000	0.000	-
HL_70420.2	1	0	0.000	0.000	-
HL_90102.1	0	0	0.000	0.000	-
HL_19399.1	0	0	0.000	0.000	-
HL_24473.3	5	0	0.000	Inf	*
HL_42436.1	2	0	0.000	Inf	*
HL_33451.1	11	1	9.091	3.898	*
HL_73465.1	36	5	13.889	3.411	*
HL_44467.1	50	11	22.000	1.684	*
HL_20920.1	13	3	23.077	1.299	*
HL_44390.1	25	6	24.000	1.267	*
HL_55202.1	16	4	25.000	2.274	*
HL_25175.1	8	2	25.000	1.949	*
HL_56809.1	114	36	31.579	1.310	*
HL_05361.1	743	253	34.051	1.398	+
HL_87223.1	213	73	34.272	1.044	*
HL_66877.1	213	78	36.620	1.275	*
HL_65071.1	1260	463	36.746	1.244	*
HL_90542.1	1233	455	36.902	1.363	+
HL_26579.1	866	320	36.952	1.221	*
HL_87554.1	2392	888	37.124	1.279	+
HL_98833.3	1041	388	37.272	1.135	*
HL_68435.1	1152	437	37.934	1.182	*
HL_45018.3	1132	438	38.693	1.175	*
HL_08203.1	62	24	38.710	1.276	*
HL_75759.4	7326	2838	38.739	1.204	*
HL_05113.1	103	40	38.835	1.310	*
HL_13707.2	95	37	38.947	1.109	*
HL_97499.1	2601	1018	39.139	1.200	*
HL_30008.1	2609	1025	39.287	1.193	*
HL_74559.1	3522	1392	39.523	1.179	*
HL_00721.1	1804	713	39.523	1.129	*
HL_35865.1	7511	2972	39.569	1.173	*

Continued on next page

Table E – continued from previous page

p-score > 0.9, 0.25 ≤ GC content ≤ 0.75					
ModelID	# original	# shuffled	FDR (%)	Odds ratio	Stat.
$\hat{F}_{rnaz}$	142 490	66 891	46.9%		
HL_27429.3	308	122	39.610	1.046	*
HL_10378.1	2055	821	39.951	1.211	*
HL_17723.4	2345	937	39.957	1.137	*
HL_17790.1	2862	1150	40.182	1.126	*
HL_78420.1	700	282	40.286	1.138	*
HL_06643.3	6962	2807	40.319	1.153	*
HL_59225.1	2680	1084	40.448	1.138	*
HL_24711.1	940	381	40.532	1.118	*
HL_35619.2	1943	788	40.556	1.152	*
HL_59604.1	2831	1149	40.586	1.152	*
HL_42969.1	32	13	40.625	1.063	*
HL_46489.2	1210	493	40.744	1.138	*
HL_01418.1	1975	808	40.911	1.117	*
HL_44522.1	214	88	41.121	1.151	*
HL_63304.1	17	7	41.176	1.706	*
HL_42677.2	5801	2390	41.200	1.126	*
HL_72273.1	982	406	41.344	1.150	*
HL_74505.1	5508	2283	41.449	1.121	*
HL_06997.2	24	10	41.667	0.975	-
HL_27397.1	470	196	41.702	1.149	*
HL_63941.1	5720	2391	41.801	1.117	*
HL_33640.1	849	356	41.932	1.034	*
HL_20028.1	212	89	41.981	1.108	*
HL_41827.1	1564	657	42.008	1.091	*
HL_24707.1	2212	930	42.043	1.103	*
HL_15603.2	107	45	42.056	1.042	*
HL_33277.1	2065	872	42.228	1.095	*
HL_18587.1	1039	440	42.348	1.155	*
HL_73972.1	1192	505	42.366	1.100	*
HL_78361.1	807	343	42.503	1.117	*
HL_57923.1	329	140	42.553	1.104	*
HL_99207.1	2260	968	42.832	1.161	*
HL_75850.1	4857	2083	42.887	1.109	*
HL_99584.1	1657	712	42.969	1.113	*
HL_00090.1	923	397	43.012	1.167	*
HL_94578.1	514	222	43.191	1.011	*
HL_52116.1	1004	434	43.227	1.067	*
HL_34027.2	9098	3939	43.295	1.078	*
HL_86115.1	5084	2202	43.312	1.088	*
HL_03785.1	4693	2036	43.384	1.090	*
HL_53789.1	2750	1195	43.455	1.029	*
HL_53015.1	729	318	43.621	1.069	*
HL_94697.1	197	86	43.655	0.941	-
HL_48254.1	1722	755	43.844	1.065	*

Continued on next page

Table E – continued from previous page

p-score > 0.9, 0.25 ≤ GC content ≤ 0.75					
ModelID	# original	# shuffled	FDR (%)	Odds ratio	Stat.
$\hat{F}_{rnaz}$	142 490	66 891	46.9%		
HL_65347.1	660	290	43.939	1.020	*
HL_90579.3	573	252	43.979	1.078	*
HL_66880.1	75	33	44.000	1.111	*
HL_50312.1	5464	2414	44.180	1.048	*
HL_30731.1	1998	885	44.294	1.051	*
HL_82288.1	731	324	44.323	1.025	*
HL_38138.1	1110	494	44.505	1.089	*
HL_57217.1	568	253	44.542	1.022	*
HL_93567.1	2362	1053	44.581	1.032	*
HL_21675.3	9426	4214	44.706	1.035	*
HL_36842.1	2510	1123	44.741	1.069	*
HL_42094.1	840	376	44.762	1.047	*
HL_30128.1	1094	490	44.790	1.086	*
HL_78507.1	2594	1163	44.834	1.060	*
HL_33875.1	844	379	44.905	1.061	*
HL_74465.5	1120	504	45.000	1.023	*
HL_66174.1	1235	556	45.020	1.050	*
HL_68081.3	1979	892	45.073	1.015	*
HL_23182.1	1482	668	45.074	1.061	*
HL_34108.2	12333	5559	45.074	1.031	*
HL_91872.1	570	257	45.088	1.068	*
HL_97270.3	3454	1558	45.107	1.036	*
HL_75579.2	19715	8899	45.138	1.041	*
HL_42077.2	18433	8321	45.142	1.036	*
HL_17008.1	31	14	45.161	1.365	*
HL_57514.2	11526	5219	45.280	1.062	*
HL_42345.1	1325	601	45.358	1.067	*
HL_12706.1	2034	923	45.379	1.003	*
HL_93530.1	1236	561	45.388	1.045	*
HL_98523.2	6011	2731	45.433	1.059	*
HL_74686.1	332	151	45.482	1.064	*
HL_48116.2	11093	5047	45.497	1.041	*
HL_58601.1	4685	2132	45.507	1.035	*
HL_62970.1	303	138	45.545	1.045	*
HL_58083.2	369	169	45.799	0.949	-
HL_13786.1	3601	1652	45.876	0.983	-
HL_96915.1	768	353	45.964	1.015	*
HL_29196.1	774	356	45.995	0.948	-
HL_18156.2	332	153	46.084	1.082	*
HL_85044.1	26	12	46.154	0.930	-
HL_48039.2	22797	10524	46.164	1.026	*
HL_65249.1	11042	5100	46.187	0.998	-
HL_19905.3	9818	4537	46.211	1.038	*
HL_84888.1	558	258	46.237	1.070	*

Continued on next page

Table E – continued from previous page

ModelID	# original	# shuffled	FDR (%)	Odds ratio	Stat.
**p-score > 0.9, 0.25 ≤ GC content ≤ 0.75**					
$\hat{F}_{rnaz}$	142 490	66 891	46.9%		
HL_19226.1	679	315	46.392	0.997	-
HL_95716.1	7023	3269	46.547	1.039	*
HL_62967.1	11991	5598	46.685	1.000	-
HL_11376.1	30015	14026	46.730	0.999	-
HL_82538.1	26675	12472	46.755	0.989	-
HL_11509.2	538	253	47.026	0.972	-
HL_62880.1	3923	1849	47.132	0.987	-
HL_72543.2	388	183	47.165	1.007	*
HL_78731.1	1108	523	47.202	1.014	*
HL_68697.2	15254	7222	47.345	0.974	-
HL_28676.1	1742	828	47.532	0.998	-
HL_80459.3	18699	8913	47.666	0.976	-
HL_76679.1	4534	2163	47.706	0.969	-
HL_21545.1	2793	1334	47.762	0.991	-
HL_87844.2	2929	1399	47.764	0.969	-
HL_82294.3	3643	1741	47.790	1.022	*
HL_93771.1	3553	1699	47.819	0.989	-
HL_01926.3	1328	636	47.892	0.998	-
HL_95049.1	881	422	47.900	0.962	-
HL_49036.3	29841	14309	47.951	0.966	-
HL_58223.1	248	119	47.984	0.909	-
HL_08382.2	19073	9160	48.026	0.960	-
HL_55718.1	10322	4960	48.053	0.992	-
HL_39895.6	7030	3382	48.108	0.970	-
HL_83865.1	6534	3147	48.163	1.000	-
HL_34440.1	2882	1389	48.196	0.887	-
HL_77692.1	784	378	48.214	0.928	-
HL_66948.1	605	292	48.264	1.008	*
HL_50059.1	817	395	48.348	0.968	-
HL_66467.1	3058	1480	48.398	0.923	-
HL_39486.1	5418	2627	48.487	0.966	-
HL_45411.1	884	429	48.529	0.931	-
HL_07915.1	687	334	48.617	0.940	-
HL_61547.5	20350	9907	48.683	0.959	-
HL_27271.2	28303	13779	48.684	0.947	-
HL_67761.1	5175	2523	48.754	0.968	-
HL_30366.1	344	168	48.837	0.869	-
HL_23290.1	1453	711	48.933	0.924	-
HL_86398.1	94	46	48.936	0.951	-
HL_35200.1	2002	981	49.001	0.939	-
HL_46570.1	1206	591	49.005	0.965	-
HL_56824.2	14644	7184	49.058	0.928	-
HL_91693.1	14027	6889	49.112	0.922	-
HL_11547.2	405	199	49.136	1.021	*

Continued on next page

Table E – continued from previous page

**p-score > 0.9, 0.25 ≤ GC content ≤ 0.75**					
ModelID	# original	# shuffled	FDR (%)	Odds ratio	Stat.
$\hat{F}_{rnaz}$	142 490	66 891	46.9%		
HL_62228.3	8153	4033	49.466	0.930	-
HL_46175.2	602	298	49.502	0.974	-
HL_24544.2	2596	1289	49.653	0.997	-
HL_76585.1	612	304	49.673	0.998	-
HL_19528.1	654	325	49.694	0.941	-
HL_18781.4	2372	1179	49.705	0.940	-
HL_62881.1	629	313	49.762	0.926	-
HL_26495.1	471	235	49.894	0.962	-
HL_96994.1	14	7	50.000	0.766	-
HL_98577.1	2	1	50.000	Inf	*
HL_23195.1	4	2	50.000	1.949	*
HL_59610.1	2194	1098	50.046	0.872	-
HL_47337.1	5519	2763	50.063	0.930	-
HL_91226.3	1755	881	50.199	0.933	-
HL_79902.1	245	123	50.204	0.960	-
HL_39872.2	833	419	50.300	0.904	-
HL_33402.3	2047	1033	50.464	0.931	-
HL_60200.1	1349	681	50.482	0.972	-
HL_88311.2	696	352	50.575	0.918	-
HL_32644.2	419	212	50.597	0.941	-
HL_72498.12	976	494	50.615	0.937	-
HL_52574.3	5595	2834	50.652	0.930	-
HL_84768.1	743	377	50.740	0.892	-
HL_67042.12	18339	9327	50.859	0.891	-
HL_67000.1	7827	3988	50.952	0.907	-
HL_82243.1	1436	732	50.975	0.940	-
HL_64543.2	1899	971	51.132	0.895	-
HL_70912.1	772	395	51.166	0.903	-
HL_85534.1	404	207	51.238	0.966	-
HL_88960.1	495	254	51.313	0.970	-
HL_17537.1	224	115	51.339	0.959	-
HL_64371.2	35	18	51.429	0.731	-
HL_68733.1	245	126	51.429	0.931	-
HL_84353.1	11526	5938	51.518	0.873	-
HL_39942.2	1119	577	51.564	0.897	-
HL_47784.2	776	402	51.804	0.900	-
HL_06122.1	2119	1103	52.053	0.882	-
HL_93263.1	401	209	52.120	0.993	-
HL_52540.1	5733	2989	52.137	0.889	-
HL_23129.1	69	36	52.174	0.840	-
HL_04194.1	380	199	52.368	0.923	-
HL_38898.1	437	229	52.403	0.871	-
HL_76766.4	10810	5666	52.414	0.866	-
HL_25124.2	5652	2977	52.672	0.851	-

Continued on next page

Table E – continued from previous page

**p-score > 0.9, 0.25 ≤ GC content ≤ 0.75**					
ModelID	# original	# shuffled	FDR (%)	Odds ratio	Stat.
$\hat{F}_{rnaz}$	142 490	66 891	46.9%		
HL_76036.3	14597	7690	52.682	0.863	-
HL_79038.1	12683	6687	52.724	0.870	-
HL_86123.2	1239	654	52.785	0.869	-
HL_29831.1	8682	4586	52.822	0.863	-
HL_99779.1	5568	2949	52.963	0.907	-
HL_38897.1	166	88	53.012	0.933	-
HL_56775.1	741	393	53.036	0.881	-
HL_65802.1	327	174	53.211	0.863	-
HL_39422.1	684	366	53.509	0.855	-
HL_48480.1	811	435	53.637	0.854	-
HL_19221.2	8074	4344	53.802	0.844	-
HL_97971.1	8042	4363	54.253	0.838	-
HL_60203.1	227	124	54.626	0.766	-
HL_49210.1	951	522	54.890	0.813	-
HL_84289.1	34	19	55.882	0.774	-
HL_57843.1	4213	2356	55.922	0.855	-
HL_80492.2	96	54	56.250	0.789	-
HL_78228.1	825	465	56.364	0.872	-
HL_55272.1	2785	1579	56.697	0.858	-
HL_85018.1	101	58	57.426	0.860	-
HL_95720.1	1323	764	57.748	0.790	-
HL_54751.2	79	46	58.228	0.871	-
HL_37962.1	309	180	58.252	0.753	-
HL_16770.1	218	128	58.716	0.791	-
HL_24108.1	76	45	59.211	0.877	-
HL_68579.1	463	275	59.395	0.762	-
HL_69403.1	208	125	60.096	0.841	-
HL_99633.1	645	391	60.620	0.797	-
HL_38130.2	37	23	62.162	0.580	-
HL_87136.2	1572	990	62.977	0.734	-
HL_19132.1	360	229	63.611	0.831	-
HL_41833.2	68	45	66.176	0.621	-
HL_98233.1	126	91	72.222	0.607	-
HL_65924.1	4	3	75.000	0.325	-
HL_42553.1	18	14	77.778	0.609	-
HL_91613.1	12	10	83.333	0.536	-
HL_59182.1	3	3	100.000	0.975	-
HL_20806.1	1	1	100.000	Inf	*
HL_25195.1	3	3	100.000	0.487	-
HL_97784.1	0	3	Inf	0.000	-
HL_49492.1	2	5	250.000	0.195	-
HL_25197.1	0	1	Inf	0.000	-
HL_19452.1	0	2	Inf	0.000	-

Table F: False discovery rates (FDR) for individual JAR3D HL models with mean interior edit distance ≤ 4 and passed cutoff ≥ 50 in windows with p-score > 0.9 and 0.25 ≤ GC content ≤ 0.75 of order R. Status "+" denotes models with adjusted p-value ≤ 0.05 and odds ratio ≥ 1.0 whereas "*" denotes models with odds ratio ≥ 1.0. Models assigned with "-" are neither enriched nor occur at higher rates in the original data. Colored rows have an FDR ≤ $\hat{F}_{rnaz}$ and original window count ≥ 6. The table is sorted in ascending order according to FDR.

**p-score > 0.9, 0.25 ≤ GC content ≤ 0.75**					
ModelID	# original	# shuffled	FDR (%)	Odds ratio	Stat.
$\hat{F}_{rnaz}$	142 475	66 891	46.9%		
HL_45358.1	0	0	0.000	0.000	-
HL_10116.1	0	0	0.000	0.000	-
HL_70420.2	1	0	0.000	0.000	-
HL_90102.1	0	0	0.000	0.000	-
HL_19399.1	0	0	0.000	0.000	-
HL_24473.3	5	0	0.000	Inf	*
HL_42436.1	2	0	0.000	Inf	*
HL_33451.1	10	1	10.000	3.373	*
HL_73465.1	35	5	14.286	3.373	*
HL_44467.1	51	11	21.569	1.796	*
HL_44390.1	25	6	24.000	1.349	*
HL_55202.1	16	4	25.000	2.249	*
HL_20920.1	12	3	25.000	1.285	*
HL_25175.1	8	2	25.000	1.927	*
HL_56809.1	119	36	30.252	1.355	*
HL_05361.1	749	253	33.778	1.423	+
HL_87223.1	212	73	34.434	1.048	*
HL_65071.1	1285	463	36.031	1.286	+
HL_26579.1	884	320	36.199	1.256	+
HL_87554.1	2439	888	36.408	1.308	+
HL_90542.1	1238	455	36.753	1.371	+
HL_98833.3	1040	388	37.308	1.135	*
HL_13707.2	99	37	37.374	1.163	*
HL_68435.1	1161	437	37.640	1.198	*
HL_66877.1	207	78	37.681	1.231	*
HL_75759.4	7488	2838	37.901	1.240	*
HL_08203.1	63	24	38.095	1.285	*
HL_97499.1	2665	1018	38.199	1.236	*
HL_30008.1	2681	1025	38.232	1.231	*
HL_74559.1	3592	1392	38.753	1.210	*
HL_00721.1	1839	713	38.771	1.168	*
HL_45018.3	1126	438	38.899	1.178	*
HL_35865.1	7623	2972	38.987	1.195	*
HL_10378.1	2097	821	39.151	1.241	*

Continued on next page

Table F – continued from previous page

ModelID	# original	# shuffled	FDR (%)	Odds ratio	Stat.
**p-score > 0.9, 0.25 ≤ GC content ≤ 0.75**					
$\hat{F}_{rnaz}$	142 475	66 891	46.9%		
HL_05113.1	102	40	39.216	1.325	*
HL_17790.1	2921	1150	39.370	1.160	*
HL_42969.1	33	13	39.394	1.095	*
HL_27429.3	308	122	39.610	1.034	*
HL_59604.1	2897	1149	39.662	1.185	*
HL_06643.3	7062	2807	39.748	1.173	*
HL_01418.1	2024	808	39.921	1.146	*
HL_24711.1	954	381	39.937	1.135	*
HL_17723.4	2344	937	39.974	1.141	*
HL_44522.1	220	88	40.000	1.180	*
HL_23195.1	5	2	40.000	2.409	*
HL_42677.2	5962	2390	40.087	1.164	*
HL_59225.1	2700	1084	40.148	1.152	*
HL_35619.2	1955	788	40.307	1.158	*
HL_78420.1	695	282	40.576	1.137	*
HL_72273.1	994	406	40.845	1.164	*
HL_46489.2	1206	493	40.879	1.138	*
HL_63304.1	17	7	41.176	1.687	*
HL_74505.1	5541	2283	41.202	1.133	*
HL_66880.1	80	33	41.250	1.234	*
HL_15603.2	109	45	41.284	1.084	*
HL_63941.1	5787	2391	41.317	1.138	*
HL_99584.1	1712	712	41.589	1.153	*
HL_33640.1	856	356	41.589	1.036	*
HL_75850.1	4978	2083	41.844	1.139	*
HL_27397.1	467	196	41.970	1.148	*
HL_20028.1	212	89	41.981	1.109	*
HL_18587.1	1048	440	41.985	1.167	*
HL_24707.1	2214	930	42.005	1.106	*
HL_41827.1	1561	657	42.088	1.091	*
HL_57923.1	332	140	42.169	1.110	*
HL_86115.1	5215	2202	42.224	1.121	*
HL_33277.1	2064	872	42.248	1.099	*
HL_03785.1	4807	2036	42.355	1.119	*
HL_53789.1	2815	1195	42.451	1.062	*
HL_73972.1	1188	505	42.508	1.094	*
HL_34027.2	9247	3939	42.598	1.099	*
HL_99207.1	2267	968	42.700	1.171	*
HL_52116.1	1013	434	42.843	1.072	*
HL_94578.1	518	222	42.857	1.021	*
HL_00090.1	925	397	42.919	1.174	*
HL_78361.1	799	343	42.929	1.108	*
HL_30731.1	2040	885	43.382	1.075	*
HL_53015.1	732	318	43.443	1.075	*

Continued on next page

Table F – continued from previous page

**p-score > 0.9, 0.25 ≤ GC content ≤ 0.75**					
ModelID	# original	# shuffled	FDR (%)	Odds ratio	Stat.
$\hat{F}_{rnaz}$	142 475	66 891	46.9%		
HL_06997.2	23	10	43.478	0.903	-
HL_48254.1	1735	755	43.516	1.071	*
HL_65347.1	666	290	43.544	1.035	*
HL_50312.1	5522	2414	43.716	1.059	*
HL_17008.1	32	14	43.750	1.397	*
HL_90579.3	575	252	43.826	1.078	*
HL_91872.1	586	257	43.857	1.108	*
HL_21675.3	9587	4214	43.955	1.055	*
HL_12706.1	2097	923	44.015	1.038	*
HL_75579.2	20137	8899	44.192	1.071	*
HL_82288.1	733	324	44.202	1.029	*
HL_42077.2	18816	8321	44.223	1.064	*
HL_38138.1	1116	494	44.265	1.092	*
HL_93567.1	2377	1053	44.300	1.047	*
HL_74465.5	1135	504	44.405	1.037	*
HL_57514.2	11751	5219	44.413	1.087	*
HL_34108.2	12515	5559	44.419	1.050	*
HL_74686.1	339	151	44.543	1.078	*
HL_58601.1	4785	2132	44.556	1.056	*
HL_94697.1	193	86	44.560	0.930	-
HL_30128.1	1097	490	44.667	1.089	*
HL_48116.2	11297	5047	44.676	1.066	*
HL_42094.1	841	376	44.709	1.040	*
HL_97270.3	3481	1558	44.757	1.046	*
HL_36842.1	2507	1123	44.795	1.067	*
HL_93530.1	1251	561	44.844	1.062	*
HL_57217.1	564	253	44.858	1.017	*
HL_66174.1	1239	556	44.875	1.057	*
HL_78507.1	2591	1163	44.886	1.060	*
HL_68081.3	1987	892	44.892	1.023	*
HL_23182.1	1488	668	44.892	1.070	*
HL_42345.1	1338	601	44.918	1.084	*
HL_33875.1	842	379	45.012	1.062	*
HL_62970.1	306	138	45.098	1.064	*
HL_98523.2	6055	2731	45.103	1.069	*
HL_19905.3	10049	4537	45.149	1.067	*
HL_13786.1	3652	1652	45.235	1.005	*
HL_48039.2	23198	10524	45.366	1.049	*
HL_29196.1	784	356	45.408	0.967	-
HL_65249.1	11230	5100	45.414	1.019	*
HL_84888.1	568	258	45.423	1.114	*
HL_96915.1	774	353	45.607	1.014	*
HL_95716.1	7160	3269	45.656	1.061	*
HL_11376.1	30689	14026	45.704	1.029	*

Continued on next page

Table F – continued from previous page

p-score > 0.9, 0.25 ≤ GC content ≤ 0.75					
ModelID	# original	# shuffled	FDR (%)	Odds ratio	Stat.
$\hat{F}_{rnaz}$	142 475	66 891	46.9%		
HL_82538.1	27251	12472	45.767	1.018	*
HL_58083.2	369	169	45.799	0.935	-
HL_18156.2	334	153	45.808	1.086	*
HL_62967.1	12214	5598	45.833	1.024	*
HL_85044.1	26	12	46.154	0.920	-
HL_68697.2	15592	7222	46.319	0.999	-
HL_62880.1	3960	1849	46.692	1.000	*
HL_76679.1	4629	2163	46.727	0.992	-
HL_78731.1	1119	523	46.738	1.027	*
HL_87844.2	2985	1399	46.868	0.987	-
HL_93771.1	3620	1699	46.934	1.011	*
HL_08382.2	19516	9160	46.936	0.989	-
HL_55718.1	10559	4960	46.974	1.020	*
HL_80459.3	18948	8913	47.039	0.991	-
HL_72543.2	389	183	47.044	1.012	*
HL_49036.3	30402	14309	47.066	0.992	-
HL_11509.2	537	253	47.114	0.978	-
HL_83865.1	6670	3147	47.181	1.024	*
HL_21545.1	2818	1334	47.339	1.003	*
HL_39895.6	7142	3382	47.354	0.986	-
HL_19226.1	665	315	47.368	0.982	-
HL_28676.1	1748	828	47.368	1.005	*
HL_34440.1	2932	1389	47.374	0.911	-
HL_66467.1	3124	1480	47.375	0.946	-
HL_82294.3	3672	1741	47.413	1.028	*
HL_66948.1	615	292	47.480	1.032	*
HL_61547.5	20837	9907	47.545	0.988	-
HL_50059.1	830	395	47.590	0.987	-
HL_67761.1	5294	2523	47.658	0.995	-
HL_27271.2	28887	13779	47.700	0.973	-
HL_01926.3	1333	636	47.712	1.000	-
HL_95049.1	884	422	47.738	0.968	-
HL_56824.2	15047	7184	47.744	0.957	-
HL_91693.1	14404	6889	47.827	0.952	-
HL_86398.1	96	46	47.917	0.964	-
HL_39486.1	5464	2627	48.078	0.977	-
HL_77692.1	785	378	48.153	0.922	-
HL_23290.1	1474	711	48.236	0.944	-
HL_07915.1	692	334	48.266	0.952	-
HL_35200.1	2031	981	48.301	0.957	-
HL_62228.3	8344	4033	48.334	0.954	-
HL_58223.1	246	119	48.374	0.909	-
HL_47337.1	5687	2763	48.584	0.960	-
HL_46570.1	1215	591	48.642	0.975	-

Continued on next page

Table F – continued from previous page

p-score > 0.9, 0.25 ≤ GC content ≤ 0.75					
ModelID	# original	# shuffled	FDR (%)	Odds ratio	Stat.
$\hat{F}_{rnaz}$	142 475	66 891	46.9%		
HL_45411.1	881	429	48.695	0.922	-
HL_19528.1	662	325	49.094	0.956	-
HL_30366.1	342	168	49.123	0.863	-
HL_18781.4	2395	1179	49.228	0.949	-
HL_46175.2	605	298	49.256	0.975	-
HL_11547.2	404	199	49.257	1.030	*
HL_24544.2	2607	1289	49.444	0.998	-
HL_67000.1	8058	3988	49.491	0.936	-
HL_59610.1	2212	1098	49.638	0.888	-
HL_67042.12	18764	9327	49.707	0.917	-
HL_79902.1	247	123	49.798	0.959	-
HL_91226.3	1769	881	49.802	0.941	-
HL_64371.2	36	18	50.000	0.749	-
HL_96994.1	14	7	50.000	0.757	-
HL_98577.1	2	1	50.000	Inf	*
HL_32644.2	424	212	50.000	0.950	-
HL_62881.1	625	313	50.080	0.921	-
HL_76585.1	605	304	50.248	0.976	-
HL_60200.1	1354	681	50.295	0.972	-
HL_84353.1	11802	5938	50.314	0.899	-
HL_88311.2	699	352	50.358	0.920	-
HL_52574.3	5627	2834	50.364	0.942	-
HL_26495.1	466	235	50.429	0.941	-
HL_33402.3	2048	1033	50.439	0.941	-
HL_72498.12	979	494	50.460	0.940	-
HL_52540.1	5911	2989	50.567	0.921	-
HL_88960.1	502	254	50.598	0.987	-
HL_47784.2	792	402	50.758	0.920	-
HL_38898.1	451	229	50.776	0.893	-
HL_68733.1	248	126	50.806	0.944	-
HL_84768.1	742	377	50.809	0.902	-
HL_85534.1	407	207	50.860	0.975	-
HL_39872.2	823	419	50.911	0.894	-
HL_64543.2	1905	971	50.971	0.897	-
HL_76766.4	11087	5666	51.105	0.893	-
HL_82243.1	1432	732	51.117	0.932	-
HL_79038.1	13061	6687	51.198	0.899	-
HL_70912.1	771	395	51.232	0.896	-
HL_76036.3	14994	7690	51.287	0.891	-
HL_17537.1	224	115	51.339	0.932	-
HL_23129.1	70	36	51.429	0.864	-
HL_25124.2	5786	2977	51.452	0.875	-
HL_29831.1	8885	4586	51.615	0.887	-
HL_39942.2	1116	577	51.703	0.891	-

Continued on next page

Table F – continued from previous page

**p-score > 0.9, 0.25 ≤ GC content ≤ 0.75**					
ModelID	# original	# shuffled	FDR (%)	Odds ratio	Stat.
$\hat{F}_{rnaz}$	142 475	66 891	46.9%		
HL_06122.1	2125	1103	51.906	0.885	-
HL_04194.1	383	199	51.958	0.927	-
HL_99779.1	5660	2949	52.102	0.923	-
HL_86123.2	1253	654	52.195	0.881	-
HL_93263.1	400	209	52.250	0.991	-
HL_56775.1	752	393	52.261	0.890	-
HL_38897.1	168	88	52.381	0.964	-
HL_19221.2	8263	4344	52.572	0.868	-
HL_97971.1	8262	4363	52.808	0.865	-
HL_39422.1	682	366	53.666	0.853	-
HL_48480.1	809	435	53.770	0.852	-
HL_65802.1	321	174	54.206	0.833	-
HL_84289.1	35	19	54.286	0.822	-
HL_60203.1	226	124	54.867	0.753	-
HL_49210.1	951	522	54.890	0.823	-
HL_57843.1	4281	2356	55.034	0.869	-
HL_78228.1	829	465	56.092	0.877	-
HL_55272.1	2796	1579	56.474	0.858	-
HL_24108.1	79	45	56.962	0.922	-
HL_95720.1	1328	764	57.530	0.798	-
HL_85018.1	100	58	58.000	0.851	-
HL_80492.2	93	54	58.065	0.746	-
HL_37962.1	306	180	58.824	0.742	-
HL_54751.2	78	46	58.974	0.876	-
HL_16770.1	217	128	58.986	0.777	-
HL_68579.1	464	275	59.267	0.767	-
HL_69403.1	209	125	59.809	0.836	-
HL_99633.1	652	391	59.969	0.809	-
HL_38130.2	37	23	62.162	0.551	-
HL_87136.2	1584	990	62.500	0.739	-
HL_19132.1	365	229	62.740	0.838	-
HL_41833.2	67	45	67.164	0.614	-
HL_98233.1	124	91	73.387	0.600	-
HL_65924.1	4	3	75.000	0.321	-
HL_91613.1	13	10	76.923	0.578	-
HL_42553.1	17	14	82.353	0.562	-
HL_59182.1	3	3	100.000	0.964	-
HL_20806.1	1	1	100.000	Inf	*
HL_25195.1	3	3	100.000	0.482	-
HL_49492.1	3	5	166.667	0.289	-
HL_97784.1	0	3	Inf	0.000	-
HL_25197.1	0	1	Inf	0.000	-
HL_19452.1	0	2	Inf	0.000	-
